# Genome-wide association study implicates novel loci and reveals candidate effector genes for longitudinal pediatric bone accrual

**DOI:** 10.1186/s13059-020-02207-9

**Published:** 2021-01-04

**Authors:** Diana L. Cousminer, Yadav Wagley, James A. Pippin, Ahmed Elhakeem, Gregory P. Way, Matthew C. Pahl, Shana E. McCormack, Alessandra Chesi, Jonathan A. Mitchell, Joseph M. Kindler, Denis Baird, April Hartley, Laura Howe, Heidi J. Kalkwarf, Joan M. Lappe, Sumei Lu, Michelle E. Leonard, Matthew E. Johnson, Hakon Hakonarson, Vicente Gilsanz, John A. Shepherd, Sharon E. Oberfield, Casey S. Greene, Andrea Kelly, Deborah A. Lawlor, Benjamin F. Voight, Andrew D. Wells, Babette S. Zemel, Kurt D. Hankenson, Struan F. A. Grant

**Affiliations:** 1 Division of Human Genetics, Children’s Hospital of Philadelphia, Philadelphia, PA USA; 2 Department of Genetics, University of Pennsylvania, Philadelphia, PA USA; 3 Center for Spatial and Functional Genomics, Children’s Hospital of Philadelphia, Philadelphia, PA USA; 4 Department of Orthopedic Surgery, University of Michigan Medical School, Ann Arbor, MI USA; 5 MRC Integrative Epidemiology Unit, Population Health Science, Bristol Medical School, University of Bristol, Bristol, UK; 6 Genomics and Computational Biology Graduate Group, University of Pennsylvania, Philadelphia, PA USA; 7 Imaging Platform, Broad Institute of MIT and Harvard, Cambridge, MA 02140 USA; 8 Division of Endocrinology and Diabetes, Children’s Hospital of Philadelphia, Philadelphia, PA USA; 9 Department of Pediatrics, Perelman School of Medicine, University of Pennsylvania, Philadelphia, PA USA; 10 Department of Pediatrics, University of Pennsylvania Perelman School of Medicine, Philadelphia, PA USA; 11 Division of Gastroenterology, Hepatology and Nutrition, Children’s Hospital of Philadelphia, Philadelphia, PA USA; 12 Department of Pediatrics, Cincinnati Children’s Hospital, University of Cincinnati, Cincinnati, OH USA; 13 Department of Medicine and College of Nursing, Creighton University School of Medicine, Omaha, NB USA; 14 Division of Pulmonary Medicine, Children’s Hospital of Philadelphia, Philadelphia, PA USA; 15 Center for Endocrinology, Diabetes & Metabolism, Children’s Hospital Los Angeles, Los Angeles, CA USA; 16 Department of Epidemiology and Population Science, University of Hawaii Cancer Center, Honolulu, HI USA; 17 Division of Pediatric Endocrinology, Columbia University Medical Center, New York, NY USA; 18 Department of Systems Pharmacology and Translational Therapeutics, University of Pennsylvania, Philadelphia, PA USA; 19 Childhood Cancer Data Lab, Alex’s Lemonade Stand Foundation, Philadelphia, PA USA; 20 Institute of Translational Medicine and Therapeutics, Perelman School of Medicine, University of Pennsylvania, Philadelphia, PA USA; 21 Department of Pathology and Laboratory Medicine, University of Pennsylvania Perelman School of Medicine, Philadelphia, PA USA

**Keywords:** Genome-wide association study, Osteoblasts, Osteogenesis, Gene mapping, Chromatin, CRISPR, Longitudinal analysis, Bone development, Skeletal development

## Abstract

**Background:**

Bone accrual impacts lifelong skeletal health, but genetic discovery has been primarily limited to cross-sectional study designs and hampered by uncertainty about target effector genes. Here, we capture this dynamic phenotype by modeling longitudinal bone accrual across 11,000 bone scans in a cohort of healthy children and adolescents, followed by genome-wide association studies (GWAS) and variant-to-gene mapping with functional follow-up.

**Results:**

We identify 40 loci, 35 not previously reported, with various degrees of supportive evidence, half residing in topological associated domains harboring known bone genes. Of several loci potentially associated with later-life fracture risk, a candidate SNP lookup provides the most compelling evidence for rs11195210 (SMC3). Variant-to-gene mapping combining ATAC-seq to assay open chromatin with high-resolution promoter-focused Capture C identifies contacts between GWAS loci and nearby gene promoters. siRNA knockdown of gene expression supports the putative effector gene at three specific loci in two osteoblast cell models. Finally, using CRISPR-Cas9 genome editing, we confirm that the immediate genomic region harboring the putative causal SNP influences PRPF38A expression, a location which is predicted to coincide with a set of binding sites for relevant transcription factors.

**Conclusions:**

Using a new longitudinal approach, we expand the number of genetic loci putatively associated with pediatric bone gain. Functional follow-up in appropriate cell models finds novel candidate genes impacting bone accrual. Our data also raise the possibility that the cell fate decision between osteogenic and adipogenic lineages is important in normal bone accrual.

## Introduction

Osteoporosis is a chronic disease characterized by low bone mineral density (BMD) and strength, which subsequently increase risk of fracture. Bone acquisition during growth is critical for achieving optimal peak bone mass in early adulthood and influences how bone density tracks throughout life [1]; individuals with higher peak bone mass ultimately have lower risk of later-life fracture [2]. Thus, understanding the factors that contribute to bone accrual has fundamental implications for optimizing skeletal health throughout life [3, 4].

Skeletal growth is a dynamic process involving bone formation driven by osteoblasts and resorption by osteoclasts. During growth, the accrual rate of areal BMD (aBMD), a measure often used to assess bone development clinically, varies by skeletal site and maturational stage [5]. BMD is highly heritable, and while > 1000 genetic variants are associated with aBMD or estimated BMD (eBMD) in adults [6–8], much less progress has been made in identifying genetic determinants of BMD during growth [9–11]. Although many adult-identified loci also associate with pediatric aBMD [12], the influences of some genetic factors are principally limited to periods of high bone-turnover, such as during bone accrual in childhood [13]. However, given that pediatric genetic studies of bone accrual to date have mainly employed cross-sectional study designs, intrinsic limits are placed on the discovery of genetic variants that influence dynamic changes in bone accrual during development.

Furthermore, because the causal effector genes at many loci identified by GWAS have not yet been identified, these signals have offered limited insight without extensive follow-up. Typically, GWAS signals have been assigned to the nearest gene, but given improvements in our understanding of the spatial organization of the human genome [14], proximity may not imply causality. As a result, variant-to-gene mapping has become an increasingly popular, evidence-based approach across a range of complex traits to link association signals to target gene(s). Chromatin conformation-based techniques that detect contacts between distant regions of the genome provide one piece of evidence connecting non-coding putative regulatory sequences harboring phenotypically associated variants to a nearby gene of interest; indeed, such data are particularly powerful when there is a paucity of eQTL data for trait-relevant cell types.

Recognizing the importance of understanding the factors influencing bone accrual to maximize lifelong bone health, we leveraged ~ 11,000 bone density measurements in the Bone Mineral Density in Childhood Study (BMDCS). By longitudinally modeling bone accrual in this cohort, we were subsequently well-placed to perform a series of genetic discovery analyses. Our approach implicated both putative causal variants and corresponding effector genes through the use of our variant-to-gene mapping pipeline [15]. We then further investigated specific loci to characterize their impact on osteoblast function in two relevant human cell models. Throughout the text, we describe loci based on the typical nearest gene nomenclature in order to orientate the reader, but we do not intend to imply that this gene is necessarily causal unless experimental evidence is found for that gene.

## Results

### Longitudinal modeling of aBMD and bone mineral content (BMC)

**Fig. 1:**
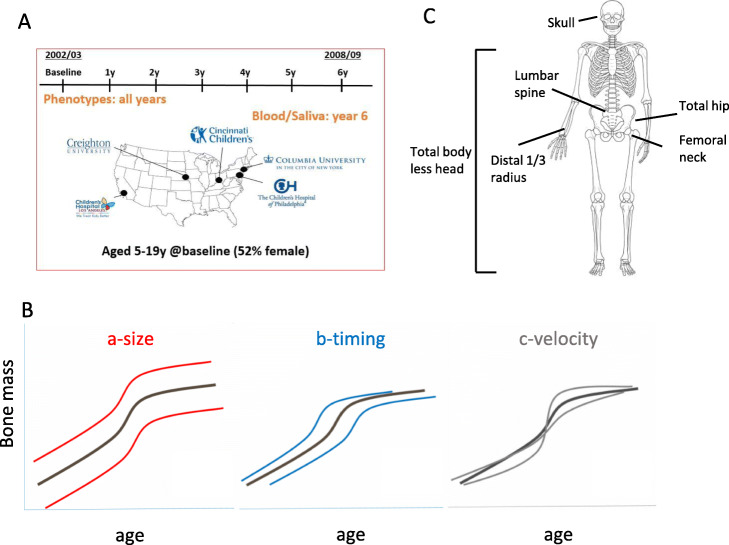
Longitudinal modeling of aBMD and BMC. **a** Bone Mineral Density in Childhood Study (BMDCS) was a multi-ethnic longitudinal prospective study of healthy children and adolescents collected over 7 years at 5 clinical sites across the USA to establish national reference curves for bone density (study visit details are given in Additional file 1: Table S1). **b** The three SITAR model parameters are a-size, representing an up-down shift on the *y*-axis for an individual compared to the population mean; b-timing, representing an earlier-later shift on the *x*-axis compared to the population mean; and c-velocity, corresponding to differences in the rate of bone accrual. **c** Six skeletal sites were assessed (total body less head, 1/3 distal radius, lumbar spine, femoral neck, total hip, and skull) for bone mineral density (g/cm^2^) and content (g). Mean SITAR curves for aBMD and BMC by sex and ancestry are shown in Additional file 2: Fig. S1

We modeled aBMD (g/cm^2^) and BMC (g/cm) from age 5 to 20 years in the BMDCS, a mixed longitudinal, multiethnic cohort of healthy children and adolescents with up to seven annual measurements (*n* = 1885). Participants were recruited to create national reference curves [16] from five sites across the USA (Fig. 1a; Additional file 1: Table S1). We modeled sex- and ancestry-specific bone accrual with “Super Imposition by Translation and Rotation” (SITAR) [17], a shape invariant model that generates a population mean curve based on all measurements. The resulting individual growth curves were then defined relative to the population mean curve by shifting in three dimensions, resulting in three random effects for each individual: *a-size:* up-down on the *y*-axis, representing differences in mean aBMD or BMC; *b-timing:* left-right on the *x*-axis, measuring differences in age when the accrual rate increases; and *c-velocity*: stretched-compressed on the age scale to measure differences in the bone accrual rate (Fig. 1b). We accessed previously derived SITAR models of BMC at the lumbar spine, total hip, femoral neck, and distal $$ \raisebox{1ex}{$1$}\!\left/ \!\raisebox{-1ex}{$3$}\right. $$ radius [18] and performed additional modeling for aBMD at these sites. We also modeled BMC and aBMD at the total body less head (TBLH) and skull (Fig. 1c). Mean curves by sex and ancestry for aBMD and BMC at the six skeletal sites are shown in Additional file 2: Fig. S1.

### Heritability of longitudinal pediatric bone density varies by skeletal site

**Fig. 2:**
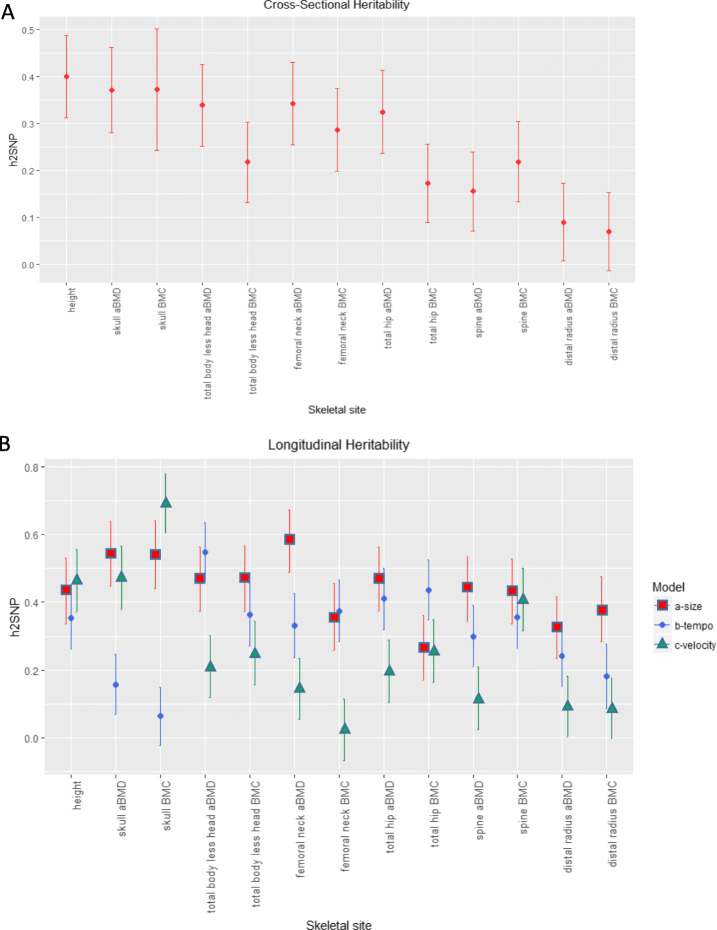
Heritability of pediatric bone density varies by skeletal site. **a** Estimates of heritability for cross-sectional baseline data. **b** Heritability estimates derived from longitudinal growth modeling with SITAR. Heritability estimates, standard errors, and *P* values are given in Additional file 1: Tables S3 & S5

To improve and extend heritability estimates of bone traits, we leveraged both directly genotyped and imputed variants to calculate SNP-heritability (*h*^*2*^_*SNP*_) for aBMD and BMC at the six skeletal sites (see Additional file 1: Table S2 for heritability power calculations). In both cross-sectional and longitudinal analyses for the *a-size* parameter, *h*^*2*^_*SNP*_ was highest for the skull and lowest for the 1/3 distal radius (Fig. 2; Additional file 1: Table S3 & S5), and the results remained largely unchanged when modeling Black or African American (AA) and non-AA participants together or separately (Additional file 1: Table S4). Therefore, for subsequent genetic analyses, we used ancestry- and sex-specific modeling results. aBMD and BMC estimates for *b-timing* varied, sometimes being substantially lower (as for the skull) or higher (as for total hip BMC) than their *a-size* counterparts. Finally, heritability estimates were overall lowest for *c-velocity*, but had a larger range, from *h*^*2*^_*SNP*_ (SE) = 0.092 (0.089), *P* = 0.15 for distal radius aBMD to *h*^*2*^_*SNP*_ (SE) = 0.69 (0.088), *P* = 2.51 × 10^−13^ for skull BMC. These results show that *h*^*2*^_*SNP*_ was robust across ancestry groups, encouraging us to consider AA and non-AA participants jointly for genetic discovery efforts, and that each of the three SITAR parameters displayed a significant genetic component across skeletal sites.

### Phenotypic and genetic correlation among skeletal sites

Phenotypically, we noted that aBMD and BMC were highly correlated with each other at each of the skeletal sites, with the TBLH, femoral neck, total hip, and spine being highest across sites (Additional file 2: Fig. S2A). In contrast, the 1/3 distal radius and skull were less correlated with the other skeletal sites, a difference which became even clearer when we performed genetic correlation analyses on the baseline and longitudinal data (Additional file 1: Table S6 & S7; Additional file 2: Fig. S2B-C). In the genetic correlation analyses, the 1/3 distal radius only showed a high correlation with TBLH. Clustering analysis of the longitudinal genetic correlations grouped most *a-size*, *b-timing*, and *c-velocity* parameters together regardless of skeletal site, with the notable exception of radius aBMD *a-size* and *c-velocity*, and skull aBMD and BMC *a-size* and *c-velocity*, which clustered into two distinct groups by skeletal site.

### GWAS reveals novel loci associated with pediatric bone accrual

Next, to identify loci associated with bone accrual, we performed 36 parallel GWAS on the three SITAR parameters (*a-size*, *b-timing*, *c-velocity*) for aBMD and BMC at the six skeletal sites (Additional file 2: Fig. S3) (*n* = 1399, 51% female, 25% Black or African American). Twenty-seven association signals achieved the traditional genome-wide significance threshold of *P* < 5 × 10^−8^, with many associated with more than one skeletal site or parameter (designated as signals 1–27, ordered by chromosome and position; Table 1). Acknowledging the large number of statistical tests performed, we used several strategies to prioritize loci for further analyses. First, given the high correlation between aBMD and BMC and among different skeletal sites (Additional file 2: Fig. S2; Additional file 1: Table S6 & S7), we used PhenoSpD [19, 20] to calculate the number of independent tests. This revealed an equivalent of 16 independent tests, resulting in a corrected significance threshold of *P* < 3.1 × 10^−9^, which yielded one locus (signal 26, rs201392388, nearest gene *FGF16*) surpassing the corrected genome-wide significance threshold accounting for multiple testing. Second, ten loci achieved a suggestive significance level (*P* = 5 × 10^−8^–1 × 10^−6^) and were supported by more than one of our phenotypes (designated signals S1-S10). Finally, we set aside three loci that reached suggestive significance in one phenotype but also gained support (*P* < 10^−4^) from a recent GWAS of adult heel eBMD in the UK Biobank [6] (designated signals S11-S13). This brought the total number of prioritized loci for follow-up assessment to 40. Overall, most loci yielded similar effect sizes in males and females and in both ancestry groups (Additional file 1: Table S8). Only one of these loci was previously associated with pediatric aBMD (signal 16, rs17140801 [9], nearest gene *RBFOX1*). In addition to the suggestive signals S11-S13, three other signals were associated with adult heel eBMD (Additional file 1: Table S10), and one signal was previously associated with adult lumbar spine aBMD [7]. In total, 35/40 (87.5%) of our signals were novel.

**Table 1:** Genome-wide significant loci (signals 1–27) and suggestive loci (signals S1-S13) supported by multiple phenotypes or skeletal sites

Signal	SNP	Phenotype	Chr	Position	A1	A2	AF^**a**^	Beta	SE	P	***r***^**2b**^	Info^**c**^	Nearest gene^**f**^	Novel^**g**^/ known	Suggestive in ALSPAC^**h**^
1	rs2762826	TBLH BMC a-size	1	52,785,140	C	T	0.071	0.425	0.075	**1.48 × 10**^**–8**^		1	*ZFYVE9* [P]	Novel	
	rs74944906	TBLH BMD a-size	1	52,779,069	G	T	0.948	− 0.438	0.089	8.91 × 10^–7^	1	1			
2	rs13407288	Femoral neck BMD a-size	2	28,611,855	G	T	0.805	0.271	0.049	**3.76 × 10**^**–8**^		0.849	*FOSL2*	Novel	
	rs13407288	Femoral neck BMD b-timing	2	28,611,855	G	T	0.805	0.243	0.049	8.08 × 10^–7^		0.849			
3	rs78996674	Total hip BMC c-velocity	3	71,412,084	T	C	0.941	− 0.469	0.082	**1.36 × 10**^**–8**^		0.988	*FOXP1*	Novel	
4	rs10804749	Femoral neck BMC a-size	3	99,179,665	T	G	0.808	− 0.267	0.049	**4.57 × 10**^**–8**^		0.998	*MIR548G,*	Novel	
	rs73138342	Femoral neck BMC c-velocity	3	99,157,560	C	T	0.847	− 0.271	0.053	3.43 × 10^–7^	0.92	0.987	*COL8A1* [P]		
	rs4453878	Femoral neck BMD a-size	3	99,170,409	C	T	0.819	− 0.251	0.050	6.64 × 10^–7^	1	0.998			
5	rs4591452	Total hip BMC c-velocity	3	149,208,719	C	T	0.893	0.352	0.060	**6.73 × 10**^**–9**^		0.99	*TM4SF4*	Novel	
	rs72615628	Total hip BMD c-velocity	3	149,210,256	A	G	0.893	0.328	0.060	5.29 × 10^–8^	1	0.99			
6	rs78543504	Total hip BMD b-timing	4	3,699,405	A	T	0.911	0.385	0.068	**2.29 × 10**^**–8**^		0.881	*ADRA2C*	Novel	
	rs78543504	Total hip BMD c-velocity	4	3,699,405	A	T	0.911	0.385	0.068	**2.29 × 10**^**–8**^		0.881			
	rs4916624	Total hip BMC c-velocity	4	3,691,543	A	T	0.927	0.368	0.072	3.84 × 10^–7^	0.97	0.882			
7	rs202099172	Distal 1/3 radius BMC b-timing	5	28,308,653	ATAT	A	0.926	0.420	0.074	**1.44 × 10**^**–8**^		0.865	*CDH9*	Novel	
	rs202099172	Distal 1/3 radius BMD b-timing	5	28,308,653	ATAT	A	0.926	0.348	0.071	9.39 × 10^–7^		0.865			
8	rs11751016	Distal 1/3 radius BMD c-velocity	6	52,017,180	G	A	0.708	− 0.228	0.042	**4.76 × 10**^**–8**^		0.99	*MIR133B, IL17A* [P]	Novel	
9	rs77977968	TBLH BMC c-velocity	7	139,335,659	C	T	0.948	0.510	0.088	**9.34 × 10**^**–9**^		0.952	*HIPK2*	Novel	
10	rs60380629	TBLH BMD c-velocity	7	151,694,110	C	T	0.935	0.454	0.078	**8.18 × 10**^**–9**^		0.979	*GALNTL5*	Novel	
	rs60380629	Total hip BMD b-timing	7	151,694,110	C	T	0.935	0.430	0.077	**2.64 × 10–**^**8**^		0.979			
	rs79289671	Total hip BMD c-velocity	7	151,692,941	C	T	0.930	0.400	0.075	9.93 × 10^–8^	0.99	0.97			
11	rs7860141	Spine BMC c-velocity	9	32,137,832	G	C	0.816	− 0.282	0.051	**4.87 × 10**^**–8**^		0.999	*ACO1*	Novel	y
12	rs1331428	TBLH BMD c-velocity	9	137,835,202	C	T	0.483	− 0.221	0.039	**2.49 × 10**^**–8**^		0.988	*FCN1*	Novel	
	rs4841948	Total hip BMD c-velocity	9	137,831,521	T	C	0.560	− 0.204	0.039	1.30 × 10^–7^	0.82	0.997			
13	rs56015205	Spine BMD b-timing	10	3,142,278	A	T	0.573	− 0.245	0.044	**3.24 × 10**^**–8**^		0.868	*PFKP*	Novel	
	rs56015205	Spine BMC b-timing	10	3,142,278	A	T	0.573	− 0.225	0.044	4.36 × 10^–7^		0.868			
14	rs11002218	Total hip BMC c-velocity	10	79,365,309	T	C	0.939	− 0.491	0.088	**3.34 × 10**^**–8**^		0.997	*KCNMA1*	Known	
	rs11002218	TBLH BMC c-velocity	10	79,365,309	T	C	0.939	− 0.451	0.089	4.94 × 10^–7^		0.997			
15	rs67416120	Femoral neck BMD b-timing	10	98,010,202	AAGAG	A	0.292	− 0.271	0.048	**2.22 × 10**^**–8**^		0.955	*BLNK*	Novel	
16	rs17140801	Femoral neck BMC c-velocity	16	6,680,114	C	T	0.868	0.319	0.055	**9.99 × 10**^**–9**^		0.964	*RBFOX1*	Known	
17	16:75655176	Distal 1/3 radius BMD b-timing	16	75,655,176	C	I	0.912	0.386	0.069	**3.19 × 10**^**–8**^		0.849	*TERF2IP* [P]	Novel	y
	rs78580335	Femoral neck BMC b-timing	16	75,787,356	T	C	0.880	0.305	0.054	**1.59 × 10**^**–8**^	0.48	0.971			
18	rs1719484	Spine BMC a-size	17	72,547,434	C	G	0.853	− 0.297	0.054	**3.80 × 10**^**–8**^		0.993	*CD300C*	Novel	
19	rs72631380	Total hip BMC a-size	17	73,102,584	G	T	0.948	0.428	0.077	**3.91 × 10**^**–8**^		0.952	*SLC16A5*	Novel	
	rs72631380	Femoral neck BMC a-size	17	73,102,584	G	T	0.948	0.420	0.077	7.24 × 10^–8^		0.952			
20	rs10415015	Distal 1/3 radius BMC b-timing	19	4,818,488	C	G	0.897	− 0.351	0.063	**2.70 × 10**^**–8**^		0.993	*TICAM1*	Novel	
21	rs116463118	Skull BMD b-timing	19	41,648,588	C	T	0.939	− 0.527	0.095	**3.47 × 10**^**–8**^		0.825	*CYP2F1*	Known	
22^e^	rs8130725	TBLH BMD b-timing	21	16,458,656	G	C	0.929	0.397	0.071	**3.17 × 10**^**–8**^		0.939	*NRIP1*	Novel	
23^e^	rs9974832	Distal 1/3 radius BMD a-size	21	16,524,340	C	T	0.902	− 0.359	0.064	**1.98 × 10**^**–8**^		0.997	*NRIP1*	Novel	
24	rs72630065	Femoral neck BMC b-timing	23	72,080,371	C	G	0.914	0.292	0.053	**4.15 × 10**^**–8**^		0.98	*DMRTC1*	Novel	
25	X:75657132	Femoral neck BMC b-timing	23	75,657,132	C	T	0.939	0.521	0.088	**4.74 × 10**^**–9**^		0.631	*SMARCA2*	Novel	
	X:75657132	Femoral neck BMC a-size	23	75,657,132	C	T	0.939	0.504	0.087	**8.47 × 10**^**–9**^		0.631			
	X:75657132	Femoral neck BMD a-size	23	75,657,132	C	T	0.939	0.475	0.083	**1.46 × 10**^**–8**^		0.631			
26	X:76607583	Femoral neck BMC a-size	23	76,607,583	A	G	0.073	− 0.353	0.057	**1.01 × 10**^**–9**^		0.952	*FGF16*	Novel	
	rs201392388	Femoral neck BMC a-size	23	76,607,584	G	A	0.073	− 0.353	0.057	**1.01 × 10**^**–9**^		0.952			
	X:76607583	Total hip BMD a-size	23	76,607,583	A	G	0.073	− 0.325	0.058	**2.06 × 10**^**–8**^		0.952			
	rs201392388	Total hip BMD a-size	23	76,607,584	G	A	0.073	− 0.325	0.058	**2.06 × 10**^**–8**^		0.952			
27	rs144238538	Femoral neck BMC a-size	23	78,179,955	C	T	0.911	0.291	0.052	**3.03 × 10**^**–8**^		0.981	*P2RY10,*	Novel	
	rs144238538	Total hip BMD a-size	23	78,179,955	C	T	0.911	0.287	0.052	**4.75 × 10**^**–8**^		0.981	*MIR4328*		
	rs7050494	Spine BMC b-timing	23	78,094,413	G	T	0.897	0.271	0.049	**4.97 × 10**^**–8**^	0.55	0.978			
S1	rs17336942	TBLH BMD a-size	2	212,861,073	T	C	0.581	0.195	0.038	3.74 × 10^–7^		0.996	*ERBB4*	Novel	
	rs17336942	Total hip BMD a-size	2	212,861,073	T	C	0.581	0.190	0.038	8.38 × 10^–7^		0.996			
	rs17336942	Total hip BMC a-size	2	212,861,073	T	C	0.581	0.191	0.038	6.64 × 10^–7^		0.996			
S2	rs77349060	Spine BMC c-velocity	2	240,273,229	C	T	0.871	0.275	0.052	1.93 × 10^–7^		0.996	*HDAC4*	Novel	
	rs4852055	Total hip BMC c-velocity	2	240,281,878	A	G	0.848	0.250	0.048	2.10 × 10^–7^	0.83	0.997			
S3	rs56883672	Femoral neck BMC c-velocity	4	106,026,989	C	G	0.595	0.208	0.038	7.12 × 10^–8^		0.999	*TET2* [P]	Known	
	rs6533178	Total hip BMC c-velocity	4	106,039,826	T	C	0.592	− 0.203	0.040	3.19 × 10^–7^	0.43	0.965			
S4	rs12641626	Femoral neck BMD a-size	4	169,760,004	G	A	0.912	0.357	0.067	1.27 × 10^–7^		0.953	*PALLD*	Novel	y
	rs12641626	Femoral neck BMC a-size	4	169,760,004	G	A	0.912	0.348	0.067	2.36 × 10^–7^		0.953			
S5	rs79651589	TBLH BMC a-size	6	143,130,718	C	CTGTTTGTT	0.313	− 0.212	0.043	7.53 × 10^–7^		0.79	*HIVEP2*	Novel	
	rs79651589	Total hip BMC a-size	6	143,130,718	C	CTGTTTGTT	0.313	− 0.213	0.043	9.26 × 10^–7^		0.79			
S6	rs142924645	Total hip BMD b-timing	12	3,162,566	C	CT	0.943	− 0.425	0.085	7.02 × 10^–7^		0.964	*TEAD4*	Novel	
	rs185315462	Spine BMD b-timing	12	3,163,396	T	G	0.945	− 0.440	0.087	4.67 × 10^–7^	1	0.964			
S7	rs45554832	TBLH BMC a-size	14	30,375,005	C	T	0.930	− 0.400	0.075	1.09 × 10^–7^		0.922	*PRKD1*	Novel	
	rs45554832	Total hip BMD a-size	14	30,375,005	C	T	0.930	− 0.394	0.076	2.22 × 10^–7^		0.922			
	rs45554832	Femoral neck BMD a-size	14	30,375,005	C	T	0.930	− 0.413	0.076	6.07 × 10^–8^		0.922			
	rs45554832	Femoral neck BMC a-size	14	30,375,005	C	T	0.930	− 0.393	0.076	2.39 × 10^–7^		0.922			
	rs74040635	Total hip BMC a-size	14	30,388,192	T	C	0.943	− 0.449	0.085	1.36 × 10^–7^	0.6	0.922			
S8	rs9962766	TBLH BMC b-timing	18	71,009,151	G	A	0.894	− 0.306	0.062	7.92 × 10^–7^		0.986	*NETO1*	Novel	
	rs10622698	TBLH BMC a-size	18	71,025,487	A	ATT	0.862	− 0.290	0.056	2.58 × 10^–7^	0.8	0.995			
S9	rs77154151	TBLH BMC c-velocity	19	3,072,434	G	A	0.877	0.332	0.065	4.08 × 10^–7^		0.795	*AES*	Novel	y
	rs12461372	Total hip BMC c-velocity	19	3,072,859	G	A	0.880	0.331	0.064	2.13 × 10^–7^	0.99	0.796			
S10	rs4283509	Femoral neck BMD c-velocity	21	23,031,423	C	A	0.882	0.292	0.057	3.79 × 10^–7^		0.995	*NCAM2*	Novel	
	21:23052938	Total hip BMC c-velocity	21	23,052,938	T	TG	0.268	− 0.237	0.047	5.62 × 10^–7^	0.36	0.841			
S11	rs2564086	Spine BMC a-size	2	5,944,498	C	T	0.274	0.225	0.045	5.21 × 10^–7^		0.98	*SOX11*	Novel^d^	y
S12	rs10866634	Distal 1/3 radius BMC a-size	5	168,748,968	T	G	0.145	0.268	0.054	9.14 × 10^–7^		0.991	*SLIT3*	Novel^d^	
S13	rs11195210	Total hip BMC a-size	10	112,357,813	G	A	0.931	0.364	0.074	9.37 × 10^–7^		0.979	*SMC3*	Known^d^	

^a^Allele frequency in all samples (see ancestry-specific frequencies in Supplementary Table 6)

^b^For loci with more than one sentinel SNP, *r*^2^ values are given between this SNP and that in the row above

^c^Info represents imputation quality

^d^Suggestive association with heel eBMD (*P* < 10^−4^) in Morris et al. 2018

^e^Despite their close physical proximity, signals 22 and 23 are not in LD (*r*^2^ = 0.05)

^f^[P] Genes implicated by promoter-interaction map

^g^Not previously reported in GWAS of bone density

^h^Full results presented in Supplementary Table 9. Y, nominal support

In line with expectations based on the phenotypic and genetic correlation results, we observed the highest number of associated loci for the total hip, femoral neck, and TBLH (Additional file 2: Fig. S4A), followed by the spine. Additionally, the majority of shared associations were observed across more than one of these four traits. Also in line with expectations based on the clustering of genetic correlation results within *a-size*, *b-timing,* and *c-velocity* for different skeletal sites, the majority of loci were associated with only one of these longitudinal parameters (Additional file 2: Fig. S4B).

### Follow-up in ALSPAC

We attempted replication of loci in the ALSPAC cohort (*n* = 6382), but were limited by the skeletal sites available. TBLH and skull BMC were modeled with SITAR, after which the *a-size*, *b-timing*, and *c-velocity* random effects were subjected to GWAS using a linear mixed model. Given the differences between the BMDCS and ALSPAC data (including different DXA machines used, populations (mixed ancestry US vs. British white), ages (beginning at age 5 years in BMDCS and 10 years in ALSPAC), cohort-specific covariates applied, and genotyping arrays employed), we opted to take a broad approach and extracted all SNPs in LD with our 40 lead signals (*r*^2^ > 0.8 in Europeans). Five of our loci replicated at a nominal significance level (Additional file 1: Table S9), one of which also showed suggestive support in the heel eBMD lookup (signal S11, rs2564086, nearest gene *SOX11*).

### Association with later-life fracture in adults

Recently, we found that the postmenopausal bone loss and fracture-associated Sp1 variant within the *COLIA1* gene^1,2^ was implicated in delayed bone gain across puberty in girls [13]. Given that both bone gain and loss are periods of relatively high bone turnover, we assessed the converse possibility: that bone accrual-associated variants might also influence later-life fracture risk. We queried our signals in a published UK Biobank GWAS of adult fracture [6], a GWAS meta-analysis of fracture [21], and a range of fracture phenotypes in the PheWEB browser. These three approaches converged on one of our suggestive signals for total hip BMC *a-size*, associated with both heel eBMD and fracture in Morris et al (signal S13, rs11195210, nearest gene *SMC3*; heel eBMD beta = − 0.02, *P* = 2.3 × 10^−8^; fracture OR = 1.04, *P* = 0.0024; Additional file 1: Table S10). In Trajanoska et al, this same signal showed suggestive association with fracture (*P* = 0.0099), and the PheWEB lookup showed associations with fracture in the last 5 years (*P* = 1.4 × 10^−3^) and leg fracture (*P* = 3.6 × 10^−3^). The PheWEB lookup also provided support for signal 22 (rs8130725, nearest gene *NRIP1*), which was associated with self-reported fracture of the radius (*P* = 8.9 × 10^−5^). Thus, we identified loci putatively active in bone metabolism both early and later in life.

### Functionally relevant candidate genes and pathways at associated loci

We next sought to identify credible candidate effector genes near the 40 prioritized loci. Given that the nearest gene to a GWAS signal is often not the causal gene, we considered all genes within the signals’ surrounding topological associated domains (TADs), regions of the genome previously defined as most likely to set the bounds where the causal effector gene resides [22]. This resulted in 319 protein-coding genes (all TAD genes are listed in Additional file 1: Table S12). We then assessed the extent of evidence supporting that this set of genes is involved in skeletal development. At 21 loci (with two harboring two distinct signals each), we observed genes known to be involved in bone biology (Table 2; Additional file 1: Table S13). Many of these genes are well-established as key players in osteogenesis or skeletal development, such as *FOSL2*, which controls osteoclast size and survival [23]; *WWTR1* (*TAZ*), encoding a key member of the Hippo pathway that interacts with RUNX2 to induce osteogenesis [24]; *SLC9A3R1* (*NHERF*), a member of the Wnt signaling pathway associated with hypophosphatemic nephrolithiasis/osteoporosis-2 (OMIM 612287) as well as low bone mineral density [25]; and *TGFB1*, mutations in which lead to Camurati-Engelmann disease (OMIM 131300) and bone density alterations [26]. We also observed important skeletal biology-related genes at suggestive loci, with these genes including *TWIST2* (Ablepharon-macrostomia syndrome and Barber-Say syndrome [OMIM 200110 and 209885, respectively], which show premature osteoblast differentiation and growth retardation [27–29]), *HDAC4* (“osteoblast differentiation and development”) [30, 31], *PRKD1* (“positive regulation of osteoclast development”) [32], *HMG20B* (“osteoblast differentiation and development”), and *SOX11* (Coffin-Siris syndrome 9 [OMIM 615866] in which there is abnormal skeletal morphology) [33]. Although these are known genes, we note that genetic associations have not been previously implicated nearby these genes in GWAS of aBMD and BMC (with the exception of the *TGFB1* locus [6]). This analysis revealed plausible candidate effector genes at half of the association signals, although direct evidence linking our signals to these genes remains to be established.

**Table 2:** Candidate genes with evidence for functional involvement in skeletal biology

Signal	Gene^**a**^	Gene Ontology or KEGG term	Mendelian disease (OMIM ID)^**b**^	Human phenotype	Mouse phenotype^**c**^	Selected references
2	*FOSL2*	Osteoclast differentiation	NA	NA	Abnormal osteoblast and osteoclast morphology and physiology; abnormal bone morphology; decreased bone mineralization; etc.	Bozec (*Nature* 2008)
3	*FOXP1*	Osteoclast differentiation and development	NA	NA	Abnormal osteoclastogenesis and bone resorption	Zhao (*Dev Biol* 2015)
4	*COL8A1*			Downregulated in aged osteoblasts	Slightly elevated bone mineral content	Zhang (*Biochem Biophys Res Comm* 2018)
5	*WWTR1 (TAZ)*	Osteoclast differentiation, mesenchymal cell differentiation	NA	NA	Abnormal skeleton morphology and bone ossification; decreased body size	Hong (*Science* 2005)
8	*IL17A* [P]	Positive regulation of osteoclast development	NA	NA	NA	Kotake (*Clin Invest* 1999)
9	*TBXAS1*	NA	Ghosal heamtodiaphyseal dysplasia (231095)	Increased bone density	NA	Genevieve (*Nat Genet* 2008)
15	*BLNK*	Osteoclast differentiation	NA	NA	NA	Shinohara (*Cell* 2008)
18	*SLC9A3R1 (NHERF)*	Wnt signaling pathway	Hypophosphatemic nephrolithiasis/osteoporosis-2 (612287)	Low bone mineral density	Decreased bone mineral content and density	Karim (*NEJM* 2008)
19	*GRB2* [P]	Osteoclast differentiation	NA	NA	Abnormal mandible morphology	Levy-Apter (*J Biol Chem* 2014)
21	*TGFB1*	Osteoclast differentiation	Camurati-Engelmann disease (131300)	Cortical thickening of the diaphyses of the long bones; some patients also show involvement of the skull; increased bone mineral density; osteopenia	Abnormal bone ossification; decreased osteoblast cell number; etc.	Kinoshita (*Nat Genet* 2000); Gao (*Proc Natl Acad Sci* 2004)
22–23	*NRIP1*	NA	NA	Differentially expressed in low and high BMD samples; may interact with ESR1	NA	Morón (*Bone* 2006); Li (*Bone Joint Res* 2016)
24	*CITED1*	Negative regulation of osteoblast differentiation	NA	NA	NA	Yang (*Endocrinology* 2008)
25–26	*ATP7A*	NA	Menkes disease (309400), Occipital horn syndrome (304150)	Defective collagen cross-linking resulting in osteoporosis and pathological fracture	Abnormal skeleton morphology; osteoarthritis	Kim (*Stem Cell Res Ther* 2015)
S2	*HDAC4*	Osteoblast differentiation and development	NA	NA	Abnormal bone ossification; abnormal osteoblast cell number; skeletal phenotype; etc.	Vega (*Cell* 2004)
	*TWIST2*	Negative regulation of osteoblast differentiation	Ablepharon-macrostomia syndrome (200110); Barber-Say syndrome (209885)	Premature osteoblast differentiation, growth retardation	Abnormal osteoblast differentiation	Bialek (*Dev Cell* 2004)
S3	*TET2* [P]	NA	Myelodysplastic syndrome, somatic (614286)	NA	Osteopetrosis, reduced osteoclast number	Chu (*Genomics, Proteomics & Bioinf.* 2018); Yang (*Nat Comm* 2018)
S6	*TEAD4*	Skeletal system development	NA	NA	NA	Matsumoto (*J Clin Invest* 2016);
	*TULP3* [P]	Bone development	NA	NA	Abnormal vertebrae morphology and development	Patterson (*HMG* 2009)
S7	*PRKD1*	Positive regulation of osteoclast development	NA	NA	Reduced bone mineral density; decreased trabecular thickness	Bollag (*Mol Cell Endocrinol* 2018)
S9	*GNA11*	Skeletal system development	Hypocalcemia (615361); hypocalciuric hypercalcemia (145981)	Altered calcium levels; short stature	Decreased bone mineral content and density	Nesbit (*NEJM* 2013)
S9	*AES (TLE5)*	Skeletal system development	NA	NA	NA	Zhao (*BBRC* 2017)
S11	*SOX11*	Positive regulation of osteoblast differentiation	Coffin-Siris syndrome 9 (615866)	Abnormal skeletal morphology	Abnormal bone mineralization and ossification; abnormal lumbar vertebrae morphology	Tsurusaki (*Nat Commun* 2014)
S12	*SLIT3*	NA	NA	Higher circulating levels associated with higher bone mass in postmenopausal women	Low bone mass	Kim (*J Clin Invest* 2018)
S13	*SMC3*	NA	Cornelia de Lange syndrome 3 (610759)	Growth retardation	Decreased bone mineral content	Andrade (*Horm Res Paediatr* 2017)

^a^[P] Genes implicated by promoter-interaction map

^b^Mendelian disease genes from the Online Mendelian Inheritance in Man (OMIM) database (https://www.ncbi.nlm.nih.gov/omim)

^c^Mouse phenotypes from the Mouse Genome Informatics database (http://www.informatics.jax.org/)

Next, we performed pathway analysis for all transcripts in the TADs corresponding to the 40 prioritized signals [34], which revealed several pathways of interest, including “long-chain fatty acid metabolic process,” “negative regulation of toll-like receptor signaling pathway,” “calcium signaling pathway,” “FoxO signaling pathway,” and “Hippo signaling pathway” (Additional file 1: Tables S14 & S15).

### Variant-to-gene mapping identifies high-confidence SNP-to-gene promoter contacts

**Fig. 3:**
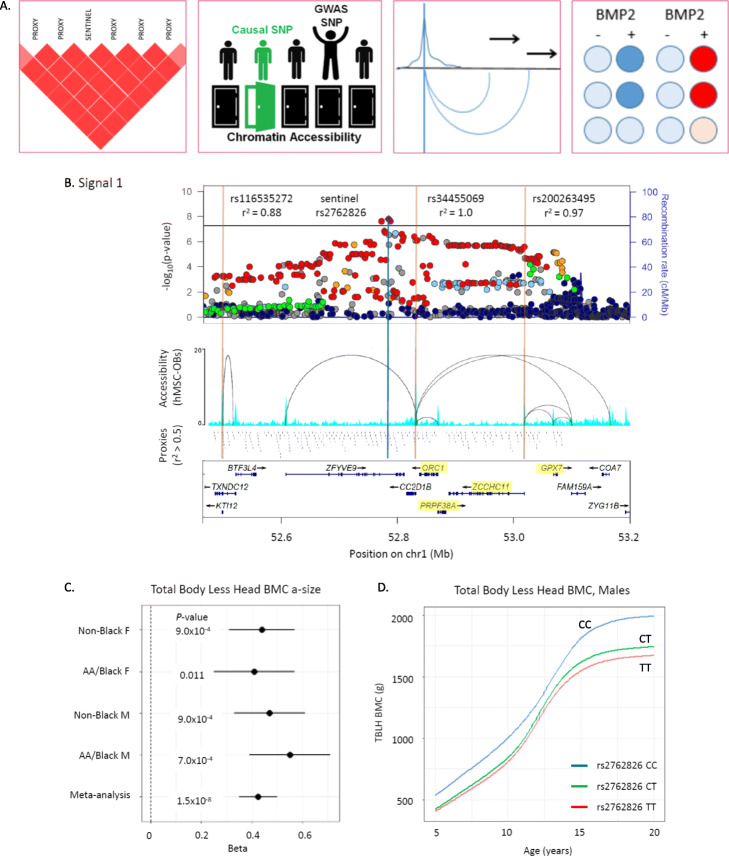
Genome-wide association and variant-to-gene mapping highlight three loci associated with pediatric bone accrual. **a** Overview of variant-to-gene mapping pipeline. We first identify all SNPs in high LD with our sentinel associated variant. These are then filtered by residing in open chromatin as assessed by ATAC-seq in hMSC-derived osteoblasts. The open chromatin variants are subsequently filtered by being in direct physical contact with gene promoter baits. Finally, siRNA knockdown experiments are performed for a subset of these contacted genes to assess the impact on osteogenesis. **b** Locus plot for signal 1, near *CC2D1B*, showing the association landscape with key SNPs marked, chromatin accessibility as assessed by ATAC-seq and Promoter CaptureC interaction loops, the locations of all proxy SNPs in the region, and the locations of genes at this locus, with the four genes followed up with in vitro functional analysis highlighted in yellow. **c** Forest plots showing the association results in each subsample (Black and Non-Black males and females). **d** Representative mean SITAR curves by genotype for rs2762826. Complete variant-to-gene mapping results are given in Additional file 1: Table S16

We then performed variant-to-gene mapping to physically connect our signals with their putative target effector genes (overview of analytical pipeline provided in Fig. 3a). In order to implicate effector genes in an appropriate tissue context, we leveraged data from human mesenchymal stem cell (hMSC)-derived osteoblasts [15]. We first extracted all proxy SNPs in LD with our lead SNPs (liberal *r*^2^ ≥ 0.5) that resided in accessible chromatin [15]. Next, we queried accessible SNP-to-gene interactions in high-resolution promoter-focused Capture C data from the same cell line. Six loci (15% of the 40 loci identified) exhibited *cis* interactions with gene promoters (Additional file 1: Table S16), with a total of 22 genes targeted by these interactions. These target genes included several prioritized by our functional candidate search, such as *GRB2* (signal 19), involved in osteoclast differentiation [35] and *TULP3* (signal S6), associated with abnormal vertebrae morphology and development [36].

Our promoter-focused Capture C data also pointed to the nearest gene at signal S3, *TET2*, a known promoter of osteogenesis [37] (Additional file 2: Fig. S5A). Interestingly, we observed two association signals at this locus, which despite being in moderate LD (*r*^2^ = 0.43) showed opposite directional effects (Table 1). These two signals were both genome-wide significant in the published heel eBMD GWAS [6] with opposite effect directions (Additional file 1: Table S10). One of our signals, rs56883672-C (FN BMC *c-velocity*, beta (SE) = 0.21 (0.038), *P* = 7.1 × 10^−8^) was in LD with an accessible proxy SNP (a SNP falling in an open ATAC-seq peak in high LD with the sentinel variant), rs2301718, which showed a *cis* interaction with *TET2* and falls in a binding site for RBPj, a primary nuclear mediator of Notch and an osteogenic driver [38] (Additional file 2: Fig. S5B). Thus, rs2301718 is a putative causal variant falling in a potential novel distal enhancer for *TET2*. Additionally, a conserved binding site for FOXO3 (determined by the Transfac Matrix Database (v7.0) in the UCSC Genome Browser), a transcription factor regulated by RBPj [39] and a member of the FOXO family which play critical roles in skeletal homeostasis [40], lies immediately upstream (Additional file 2: Fig. S5C). Thus, this may be a regulatory region for osteogenesis with dynamic effects at different skeletal sites.

Although GTEx data was not generated for bone, we searched for pan-tissue eQTLs that would provide another line of evidence linking our loci to effector genes. We identified SNPs in high LD (*r*^2^ > 0.8) with the 40 sentinel SNPs, queried significant eQTLs in all available GTEx tissues [41], and observed LD-eQTLs for 28 genes (Additional file 1: Table S17). None of the genes highlighted by our functional search overlapped with LD-eQTL genes, suggesting tissue-specific or temporal-specific regulation that is not reflected in this broad pan-tissue context. However, our promoter Capture C approach did support several genes with LD-eQTL evidence, namely *ADAT1*, *GPX7*, *ORC1*, *PRPF38A*, and *ZCCHC11*. Colocalization evidence would be needed to definitively link our GWAS signals with these genes in the developing skeleton, but due to the lack of relevant eQTL datasets in growing bone, we instead used this LD-eQTL pan-tissue evidence to help prioritize loci for functional in vitro follow-up.

### Functional assays in bone cell lines implicates one novel gene each at three loci

About half of our prioritized loci lacked clear functional candidate effector genes, with three signals showing evidence of target gene promoter interactions in our hMSC-derived osteoblasts (Additional file 2: Fig. S6). Two of these loci (signals 1 and 17, near *CC2D1B* and *TERF2IP*, respectively) proved more challenging to resolve given they both had multiple gene contacts in our hMSC-derived osteoblast atlas (Fig. 3b–d; Additional file 2: Fig. S7A) as well as pan-tissue LD-eQTL support. To identify novel genes involved in bone mineralization, we followed up putative candidate effector genes identified by variant-to-gene mapping at these two loci. Another locus had promoter interaction and LD-eQTL support for more than one plausible candidate effector gene, so we also aimed to clarify this observation (signal S6; Additional file 2: Fig. S7B). We performed siRNA knockdown of four genes at each locus (for a total of 12 genes) in primary hMSCs and assessed osteoblast differentiation. qPCR analysis revealed that each siRNA resulted in specific, significant knockdown of its corresponding target under unstimulated conditions (Additional file 2: Fig. S8).

**Fig. 4:**
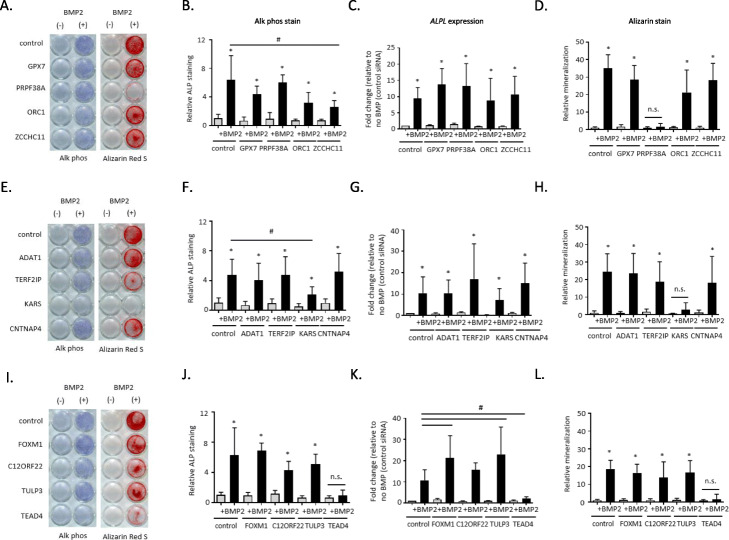
Functional assays in human mesenchymal stem cell-induced osteoblasts following siRNA knockdown of four genes each at three loci implicated by GWAS and variant-to-gene mapping. **a**, **e**, **i** Representative alkaline phosphatase (blue) and Alizarin Red S (red) stains for osteoblastic activity and calcium deposition, respectively, for the three tested loci. Experiments were performed twice in three unique donor lines. **b**, **f**, **j** Quantification of alkaline phosphatase staining using quantitative image analysis was repeated twice with three different independent hMSC donor cell lines. **c**, **g**, **k** *ALPL* gene expression. **d**, **h**, **l** Quantification of Alizarin Red S staining. *p < 0.05 comparing no treatment to BMP treatment for each siRNA, ^#^*p* < 0.05 comparing control siRNA to siRNA for gene of interest, n.s = not significant. Error bars represent standard deviation. hMSC donor line, siRNA, and qPCR details are given in Additional file 1: Tables S19-S21

To identify which contacted genes have roles in osteoblast function, we examined osteoblast activity with histochemical alkaline phosphatase (ALP) and mineralization with Alizarin red S staining. We found that disruption of just one gene per locus among each group of four candidates showed a significant reduction in terminal osteoblast differentiation. Although additional candidate genes are present at the signal 1 locus, including three with nonsynonymous variants in LD (*r*^2^ > 0.8) with the sentinel SNP (the nearest gene *CC2D1B*, *ZFYVE9*, and *KTI12*), we opted to target genes with Capture C and LD-eQTL evidence. While targeting *GPX7*, *PRPF38A*, *ORC1*, or *ZCCHC11* at signal 1 produced somewhat variable ALP staining across donor lines, the staining levels (Fig. 4a, b) and corresponding *ALPL* gene expression levels (Fig. 4c) were not significantly different from non-targeted cells. On the other hand, there was a marked reduction in Alizarin red S staining after *PRPF38A* knockdown (Fig. 4a, d).

At the signal 17 locus (targeting *ADAT1*, *TERF2IP*, *KARS*, and *CNTNAP4*), downregulation of *KARS* produced a significant reduction in ALP staining and extracellular calcium deposition, but we did not observe a significant reduction in *ALPL* gene expression itself (Fig. 4e–h). To further understand the discrepancy between ALP staining and gene expression patterns, we individually examined the ALP expression and staining pattern for each donor line. A consistent reduction in *ALPL* gene expression and ALP staining was clearly evident in two out of the three donor lines, but despite a reduction of ALP staining in the third line, *ALPL* gene expression was increased (data not shown). Despite the donor variability seen in our experiments, male mice with a heterozygous *KARS* knockout have a significant increase in BMD excluding skull (male *P* = 6.61 × 10^−5^; significance threshold 1 × 10^−4^; https://www.mousephenotype.org/), providing orthogonal evidence for the importance of this gene in osteogenesis.

At signal S6 (targeting *FOXM1*, *C12ORF22*, *TULP3*, and *TEAD4*), although there are several plausible functional candidate genes (Table 2), targeting *TEAD4* significantly reduced both ALP staining and *ALPL* gene expression as well as extracellular calcium deposition (Fig. 4i–l). In contrast, gene knockdowns for the other candidates had no impact on these readouts. These results were subsequently replicated in siRNA knockdown experiments in an immortalized human fetal osteoblast (hFOB) cell line (Additional file 2: Fig. S9).

Activation of canonical BMP signaling leads to the phosphorylation of SMAD proteins and upregulation of the *ID1* family of genes. Thus, we assessed BMP2 signaling by measuring *ID1* gene expression and assessed expression of pro-osteoblastic transcription factors *RUNX2* and *SP7*. After *PRPF38A* and *KARS* knockdown, BMP2 signaling was intact and the expression of *RUNX2* and *SP7* were preserved (Additional file 2: Fig. S10A-F). For cells lacking *TEAD4*, *ID1* expression was unchanged, although *RUNX2* and *SP7* expression levels were lower than observed in controls (Additional file 2: Fig. S10G-I). These results suggest that these three genes impact osteogenesis via distinct molecular mechanisms.

### PRPF38A knockdown induces morphological changes

**Fig. 5:**
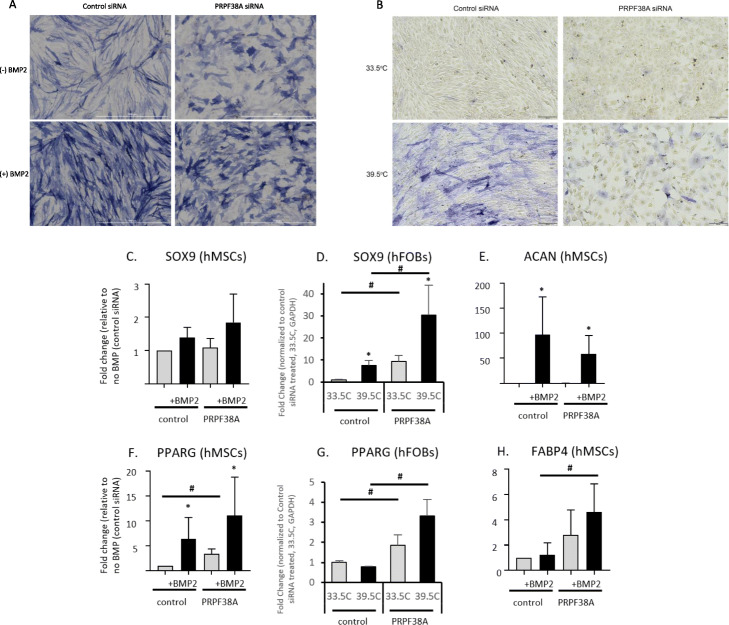
*PRPF38A* knockdown induced a morphological change in two osteoblast cell models. **a** Cell morphology in hMSCs before (top) and after BMP2 treatment (bottom) with control siRNA (left) and *PRPF38A* knockdown (right). Representative color bright-field images of a typical alkaline phosphatase stained plate from *PRPF38A* silenced cells is shown. Similar morphological changes were observed for all three donor lines used in the study. Scale bar, 1000 μm. **b** *PRPF38A* knockdown-induced morphological changes were recapitulated in human fetal osteoblasts under permissive growth (33.5 °C; top) and differentiation (39.5 °C; bottom) conditions. Scale bar, 200 μm. **c**–**e** Quantitative gene expression of chondrocytic genes *SOX9* and *ACAN* and **f**–**h** adipocyte-specific genes *PPARG* and *FABP4*. For hMSCs, data is from two technical replicates from three unique donor lines were averaged. For hFOBs, three technical replicates were averaged. Levels of *ACAN* and *FABP4* were undetectable in hFOBs, even in reactions with up to 600 ng of cDNA (twice the amount used for qPCR of other targets). *p < 0.05 comparing non-treated to treated cells (BMP2 or 39.5 °C for hMSCs and hFOBs, respectively) for each siRNA, ^#^*p* < 0.05 comparing control siRNA to siRNA for gene of interest. Error bars represent standard deviation

At signal 1, *PRPF38A* silencing resulted in a morphological change and reduction of extracellular calcium deposition that was evident in both hMSC-derived osteoblasts (Fig. 5a) and hFOBs (Fig. 5b). Thus, we examined whether *PRPF38A* silencing affected the expression of chondrocyte specific genes *ACAN*, *COMP*, and *SOX9* or the expression levels of later osteoblastic genes *IBSP* and *OMD* in hMSCs. Despite some variability in our observations, our results largely showed that neither chondrocyte lineage genes nor later osteoblast specific genes were greatly altered in *PRPF38A* silenced cells (Fig. 5c–e; Additional file 2: Fig. S11A-C). In contrast, our results for *PRPF38A* silencing in the context of the expression of adipogenic-specific genes were more striking. *PRPF38A* silencing was sufficient to increase expression of *PPARG*, a critical transcription factor for adipocyte differentiation, and its expression increased further upon stimulation with BMP2 in hMSC-derived osteoblasts and recapitulated in hFOBs (Fig. 5f, g). Likewise, *FABP4* significantly increased in *PRPF38A* silenced cells (Fig. 5h). However, *C/EBPA* expression did not change dramatically (Additional file 2: Fig. S11D). We did not observe morphological differences in the *KARS* or *TEAD4* silenced donor lines (data not shown).

### CRISPR-Cas9 deletion of putative enhancer element for PRPF38A expression

**Fig. 6:**
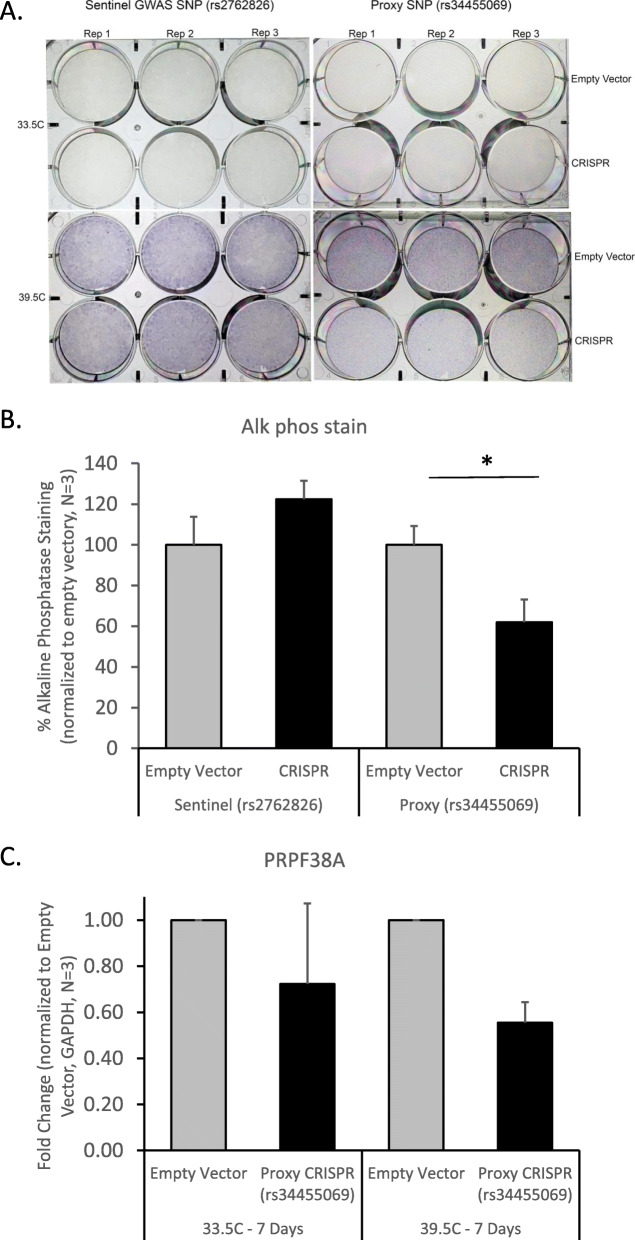
CRISPR-Cas9 deletion of sentinel and proxy SNPs at *PRPF38A* locus in hFOB cells. Only modulation of the proxy SNP impacts alkaline phosphatase level and expression of the gene. **a** Alkaline phosphatase staining was performed in triplicate after excision of the sentinel GWAS SNP (rs2762826; left) and the proxy SNP (rs34455069; right). **b** Quantification of alkaline phosphatase staining using quantitative image analysis showed that staining was reduced after excision of the region surrounding the proxy SNP, but not the sentinel SNP. **c** Gene expression of *PRPF38A* was reduced after excision of the proxy SNP. *p < 0.01, ^#^*p* < 0.001, comparing empty vector to CRISPR cells (averaged across three technical replicates). Error bars represent standard deviation. Transduction efficiency and sequencing results of the CRISPR cells are shown in Additional file 2: Fig. S11

Given the evidence for *PRPF38A* as a novel factor influencing osteogenesis in the two bone cell models, we next performed CRISPR-Cas9 deletions in hFOBs to confirm the accessible proxy SNP (rs34455069) resides within an enhancer impacting the expression of *PRPF38A*. Despite only having ~ 60% transduction efficiency in the proxy SNP-deleted cells (7–37% wild type cells; Additional file 2: Fig. S12A-B), deletion of 123-533 bp encompassing rs34455069 resulted in a 38% decrease in ALP staining (*P* = 0.005, compared to empty vector control; Fig. 6a, b) and a 45% decrease in *PRPF38A* expression (*P* = 0.0009, compared to empty vector control; Fig. 6c) as measured by qPCR. No morphological changes were observed in the proxy-SNP edited cells. We also deleted a 733-1823 bp region around the sentinel GWAS SNP (rs2762826); as expected, perturbing the immediate region harboring the sentinel GWAS SNP had no effect on ALP staining (Fig. 6a, b) or cell morphology. Intriguingly, rs34455069 is predicted as “likely to affect binding” (RegulomeDB; regulomedb.org) of two transcription factors with known regulatory effects in osteogenesis, KROX [42], and SP1:SP3 [43, 44] (position weight matrices for these binding sites highlighting this SNP are given in Additional file 2: Fig. S13). Further work is warranted to confirm that this genetic variant, a single base-pair indel, results in differential binding of these transcription factors and altered gene expression of *PRPF38A*.

## Discussion

To complement cross-sectional genetic studies of bone trait measurements and our previous work implementing linear mixed models to integrate longitudinal measures [12], as well as to target the most dynamic changes in bone density, i.e., during childhood, we performed longitudinal modeling. To assess the genetic contribution to these traits, we performed heritability analyses and GWAS, identifying 40 loci, which triples the number of identified pediatric aBMD and BMC-associated loci. Our efforts to map target genes via Capture-C data implicated leads for several putative effector genes at associated loci, and we functionally characterized selected genes in two osteoblast cell models, revealing three key candidate effector genes for further study. Thus, our longitudinal approach not only revealed novel associations with pediatric bone accrual, but also the most likely functional target genes.

We noted differences in heritability among skeletal sites, and both phenotypic and genetic correlation estimates were in line with expectations based on bone composition, degree of load bearing, and timing of development. For instance, the total hip and femoral neck, followed by the spine, were the most correlated with each other and resulted in the most shared genetic associations, together with the TBLH. Indeed, these three skeletal sites are weight-bearing and are made up of a mix of trabecular and cortical bone [45]. Additionally, they collectively make up a large proportion of the TBLH measure, and the total hip includes the femoral neck. In contrast, the 1/3 distal radius is less load-bearing and is composed nearly entirely of cortical bone, and the skull is distinct both in its bone structure and timing of growth; the majority of skull growth is completed by age 5 and is relatively stable until puberty [46].

Focusing on bioinformatic characterization of the 40 prioritized loci, at half of these, we identified known bone-related genes residing within the corresponding TADs. Among these, WWTR1/TAZ, HDAC4, TWIST2, and PRKD1 are known to interact with RUNX2, an essential osteoblastic differentiation factor. Several genes harbor Mendelian mutations resulting in abnormal bone density or skeletal phenotypes [23–27, 35, 36, 47–62], and many also show abnormal mouse skeletal phenotypes (Table 2). Although further work is needed to concretely link many of these GWAS-implicated variants to their corresponding target effector genes, our promoter Capture-C approach did corroborate some of these genes as putative functional effector genes acting in bone accrual.

In previous work, we noted a genetic variant principally active during periods of high turnover at *COLIA1*, with implications for both delayed bone accrual in girls during puberty [13] and post-menopausal bone loss and fracture [63, 64]. Three of our signals showed evidence of association in a GWAS of adult fracture [6], four with a GWAS meta-analysis of fracture, and eight with other fracture traits on the PheWEB browser. At signals S13 and 22, we also noted candidate genes with literature support: the nearest gene at signal S13 (notably supported by all three fracture look-ups) is *SMC3*, underlying Cornelia de Lange syndrome 3 (OMIM 610759) [65] and decreased BMC in mice, and the nearest gene to signal 22 is *NRIP1*, which is differentially expressed in patients with low vs. high BMD [52]. Thus, the *COLIA1* locus is unlikely to be the only factor influencing both bone gain and loss, and further investigation of the gene targets at these loci may provide leads to maximizing lifelong bone health.

Including genes at signals associated with various skeletal sites and parameters, pathway analysis pointed toward pathways known to be involved in bone metabolism. Several pathways were involved in long-chain fatty acid metabolism, largely driven by a cluster of cytochrome P450 (CYP) genes at a single locus (signal 21), which also harbors *TGFB1*. Although *TGFB1* is a plausible candidate gene, CYP genes metabolize eicosanoids (long-chain fatty acids) including arachidonic acid and affect metabolite levels [66], and genetic variation in related genes (*CYP-17* and *19*) was associated with serum sex steroid concentrations and BMD, osteoporosis, and fracture in post-menopausal women [67]. Studies have shown that osteoblasts take up and metabolize fatty acids for matrix production and mineralization [68], and long-chain fatty acids were associated with peak aBMD and bone accrual in late-adolescent males [69].

Three loci harboring five genes (*PTPRS*, *CD300A*, *CACTIN*, *CD300LF*, *TICAM1*), were annotated with the GO term “negative regulation of toll-like receptor signaling pathway.” The toll-like receptor pathway has multifaceted roles in osteoblast function, including mediating bone inflammatory responses and regulating cell viability, proliferation, and osteoblast-mediated osteoclastogenesis [70]. Finally, the “calcium signaling pathway,” the “FoxO signaling pathway” [71, 72], and the “Hippo signaling pathway” [73] are fundamental in normal skeletal development.

Notably, several candidate genes are involved in TH17 cell differentiation (*IL17F*, *IL17A*, *RXRA*, and *TGFB1*). *IL17A* is a T cell derived growth factor for MSCs [74, 75], and we observed an open proxy variant contact with the *IL17A* promoter in TH17 cells but not in osteoblasts (Additional file 1: Table S18), supporting *IL17A* as the effector gene at this locus. Expression of *IL17A* inhibits the osteogenic differentiation of MSCs [76, 77], and T cell-derived IL17A is involved in bone loss and postmenopausal osteoporosis [78, 79]. Our data shows that the well-established osteo-immune link [80, 81] could play a role in normal variation of skeletal mineralization.

Next, we performed physical variant-to-gene mapping in hMSC-derived osteoblasts, particularly important in the context of bone given that publically available genomic resources typically lack bone data. Using a previously successful approach for identifying target genes at known aBMD-associated loci [15], we identified three loci for functional follow-up that each had several potential target effector genes. After siRNA knockdown of 12 genes (4 at each locus), we observed reduced osteoblastic activity and/or reduced mineralization for one gene at each locus (*PRPF38A*, *KARS* and *TEAD4*, each among the top 70% of expressed genes in hMSC-derived osteoblasts). Two of these genes, *PRPF38A* and *KARS*, are novel in the context of bone. *KARS* encodes the multifunctional protein lysyl-tRNA synthetase, which catalyzes the attachment of amino acids to their cognate tRNAs, but also acts as a signaling molecule when secreted and induces dendritic cell maturation via the MAPK and NFkB pathways [82, 83]. On the other hand, *TEAD4* interacts with WWTR1/TAZ transcription co-activators that allow cells to escape negative regulation by the Hippo signaling pathway and undergo increased cell proliferation, the epithelial-mesenchymal transition, and expression of proteins that directly regulate cell adhesion [84]. Osteoblast differentiation is a multi-step process involving the integration of multiple signaling factors, each with its own critical role, and future studies are warranted to dissect which signals are affected by *PRPF38A*, *KARS*, and *TEAD4* silencing.

Knockdown of *PRPF38A* induced a dramatic morphological change in both hMSC-derived osteoblasts and hFOBs, concurrent with increased expression of adipogenic transcription factors *PPARG* and *FABP4*, suggesting that gene-targeted cells may favor adipogenic differentiation. Little is known about *PRPF38A* or its encoded protein, likely a member of the spliceosome complex that contains a multi-interface protein-protein interaction domain [85]. We recently reported that knockdown of two pediatric aBMD-associated genes, *ING3* and *EPDR1*, resulted in reduced mineralization and also favored adipogenesis [15]. The tightly controlled MSC lineage commitment to adipocytes or osteoblasts is critical for maintaining bone homeostasis [86] and has been implicated in conditions with abnormal bone remodeling (with increased bone marrow adiposity in osteoporosis [87, 88] and bone loss conditions [89]). In addition to *PRPF38A*, *ING3*, and *EPDR1*, previous studies suggested that *TEAD4* may promote adipogenesis [90] in conjunction with *WWTR1*/*TAZ* (signal 5) and that *TGFB* (signal 21) induces a switch from adipogenic to osteogenic differentiation in hMSCs [91]. Further studies are warranted to fully explore the hypothesis that adipogenic vs. osteogenic differentiation is a key feature of pediatric bone accrual.

Overall, we used several strategies to prioritize loci for further analysis, which in turn led to a number of validated leads. In previous studies, we did not correct for testing of multiple skeletal sites, given the high correlation between aBMD and BMC and among these different skeletal sites (Additional file 2: Fig. S2; Additional file 1: Table S6 & S7). Had we used an extremely strict correction (PhenoSpD [19, 20] was used to determine that the corrected significance threshold would be *P* < 3.1 × 10^−9^), only one locus (signal 26, rs201392388, nearest gene *FGF16*) would have surpassed this bar, and we would have missed many of our key leads. Indeed, given that subsequent bioinformatic and in vitro follow-up further supported at least half of these loci as true positives, using a more standard significance threshold initially allowed us to “rule in” novel candidate loci that ultimately led to novel candidate genes. This approach thus advocates for a more inclusive initial approach followed by multiple lines of orthogonal evidence to build functional support for specific loci [92], especially for phenotypes where it is difficult to collect large numbers of samples to have adequate statistical power to meet the traditional genome-wide significance threshold. Still, there is potential for false positives among our results, and there is the possibility that variants in moderate pairwise LD do not reflect the same underlying signal. Additionally, we have tested selected candidate genes to “rule in” their effects on bone biology; this does not exclude other potential target genes as functionally relevant. Therefore, additional replication by other studies is required.

In conclusion, we leveraged a longitudinal modeling approach to both maximize the data available in our cohort and to investigate the genetic determinants of pediatric bone accrual. Our findings suggest that differences in bone accrual attributable to genetic variation are a mechanism linking several of our loci with established associations with later-life fracture risk [6]. Finally, we identified two novel candidate effector genes at two associated loci with no obvious leads and resolved a functional candidate gene among several possible genes at a third locus. At *PRPF38A*, our data strongly supports a putative causal candidate variant, which falls into binding motifs for two relevant transcription factors. Our findings implicate multiple biological pathways involved in variation in bone accrual, and highlight the switch between osteogenesis and adipogenesis as potentially critical in pediatric bone accrual. In conclusion, in-depth longitudinal phenotyping plus appropriate functional follow-up of GWAS loci can yield greater insight into dynamic, complex traits such as bone accrual.

## Methods

### Study cohorts

The BMDCS was a longitudinal, multiethnic cohort of healthy children and adolescents who were recruited from five clinical sites across the USA (Philadelphia, PA; Cincinnati, OH; Omaha, NB; Los Angeles, CA; and New York, NY) to establish aBMD and BMC reference curves [16]. Briefly, the participants were aged 6–16 years at baseline (2002–2003) and were followed for up to 6 additional annual visits (for a maximum total of 7 visits). Older (age 19 years) and younger (age 5 years) participants were subsequently recruited in 2006–2007 and were followed annually for 2 years (maximum 3 visits). One thousand eight hundred eighty-five participants (52% female) had both phenotypes and genetic data and were thus eligible for inclusion in the present study. Participants 18 years and older gave written informed consent. Parental or guardian consent plus participant assent were obtained for subjects younger than 18 years old. The study was approved by the Institutional Review Board of each respective clinical center. This study was performed in compliance with the Helsinki Declaration.

ALSPAC [93, 94] is a prospective birth cohort study that recruited all pregnant women residing within the catchment area of 3 National Health Service authorities in southwest England with an expected date of delivery between April 1991 and December 1992. In total, 15,454 eligible pregnancies were enrolled in ALSPAC (75% response), with 14,901 live births alive at age 1 year. Detailed information has been collected from offspring and parents using questionnaires, data extraction from medical records, linkage to health records, and dedicated clinic assessments up to the last completed contact in 2018. Details of all available data can be found in the ALSPAC study website (http://www.bristol.ac.uk/alspac/researchers/our-data/), which includes a fully searchable data dictionary and variable search tool. Ethics approval was obtained from the ALSPAC law and ethics committee and local research ethics committees. Written informed consent was obtained from all participants. For this study, analysis was performed in white participants (> 98% of the sample).

### aBMD and BMC measurement

In the BMDCS, DXA scans of the whole body, lumbar spine, hip, and 1/3 distal radius were obtained using bone densitometers (Hologic, Bedford, MA, USA) following the manufacturer’s guidelines by trained research technicians. The scans were analyzed by the DXA Core Laboratory (University of San Francisco, San Francisco, CA, USA) using Hologic software (v.12.3) for baseline scans and Apex 2.1 for follow-up scans using the “compare” feature. Scans were adjusted for calibration differences among clinical centers and longitudinal drift. aBMD and BMC *Z*-scores for age, sex, and population ancestry were calculated and adjusted for height *Z*-scores [95]. For growth modeling, unadjusted aBMD or BMC values were used.

In ALSPAC, all participants were invited to undergo up to 6 whole-body DXA scans as part of research clinic assessments at mean ages 9.8, 11.7, 13.8, 15.4, 17.8, and 24.5 years. Scans were performed using a Lunar Prodigy scanner (Lunar Radiation Corp) and were analyzed according to the manufacturer’s standard scanning software and positioning protocols. Scans were reanalyzed as necessary to ensure optimal placement of borders between adjacent subregions, and scans with anomalies were excluded. From these whole-body DXA scans, we extracted BMD and BMC at each age for TBLH and skull.

### Longitudinal modeling of bone accretion

SITAR was used to model individual growth curves separately by sex and ancestry [17]. SITAR is a shape invariant model that generates a mean curve for all included measurements. Individual curves are then described relative to the mean curve by shifting in three dimensions: up-down on the *y*-axis (differences in mean size, i.e., bone density or content, between subjects relative to the population mean, *a-size*), left-right on the *x*-axis (differences in age when the rate of growth increases, *b-timing*), and stretched-compressed on the age scale to represent distance over time (how quickly growth occurs, or differences in the rate of bone mineralization in the context of the current study, *c-velocity*). These are estimated as random effects that summarize the difference of each individual growth curve relative to the population mean.

In BMDCS, we performed growth modeling on height and aBMD and BMC measured at the spine, total hip, femoral neck, distal 1/3 radius, skull, and total body less head (TBLH), as previously described [18]. Modeling was performed on up to 2014 children and adolescents (50.7% female and 23.8% self-reported as Black or African American) with a mean of 5 annual study visits each, representing ~ 11,000 scans in total (Additional file 1: Table S1; Additional file 2: Fig. S1). Only participants with genetic data and phenotypes were taken forward for heritability and GWAS analyses (max *N* = 1399, 51% female, 25% Black or African American).

In ALSPAC, SITAR models were fitted for individuals with at least 1 measurement and were fitted in males and females separately. Initially, the models were fitted to the ALSPAC data alone, and then again with the BMDCS data added. For the combined analyses, fixed effects were included in the model to distinguish between the two cohorts. We were only able to achieve converged models for both sexes for TBLH BMC and skull BMC while modeling both cohorts together. The random-effects (a, b and c) from the fitted models for TBLH and skull BMC (both sexes) were extracted for the ALSPAC participants, converted to sex-specific *z*-scores, and taken forward for GWAS replication. We included data from 6382 participants (50% female) (Additional file 2: Fig. S14).

### Genotyping and imputation

In BMDCS, genome-wide genotyping was carried out on the Illumina Infinium Omni Express plus Exome BeadChip (Illumina, San Diego, CA) at the Children’s Hospital of Philadelphia Center for Applied Genomics [96]. Quality control was subsequently performed to exclude samples with incorrect or ambiguous gender and with missingness per person > 5%, and to exclude variants with call rate < 95% and minor allele frequency (MAF) < 0.5%. Imputation was performed against the 1000 Genomes Phase 1 v.3 reference panel as previously described [9]. After imputation, variants with MAF < 5% or quality score (INFO) < 0.4 were excluded, yielding 7,238,679 SNPs.

ALSPAC children were genotyped using the Illumina HumanHap550 quad chip genotyping platform (Illumina) by 23andme subcontracting the Wellcome Trust Sanger Institute (Cambridge, UK) and the Laboratory Corporation of America (Burlington, NC, USA). The resulting raw genome-wide data were subjected to standard quality control methods. Individuals were excluded on the basis of gender mismatches, minimal or excessive heterozygosity, disproportionate levels of individual missingness (> 3%), and insufficient sample replication (IBD < 0.8). All individuals with non-European ancestry were removed. SNPs with a minor allele frequency of < 1%, a call rate of < 95%, or evidence for violations of Hardy-Weinberg equilibrium (*P* < 5 × 10^−7^) were removed. Cryptic relatedness was measured as proportion of identity by descent (IBD > 0.1). Related subjects that passed all other quality control thresholds were retained during subsequent phasing and imputation. Nine thousand one hundred fifteen subjects and 500,527 SNPs passed these quality control filters. Of these, we combined 477,482 SNP genotypes in common between the sample of ALSPAC children and ALSPAC mothers. We removed SNPs with genotype missingness above 1% due to poor quality (11,396 SNPs removed) and removed a further 321 subjects due to potential ID mismatches. We estimated haplotypes using ShapeIT (v2.r644) which utilizes relatedness during phasing. The phased haplotypes were then imputed to the Haplotype Reference Consortium (HRCr1.1, 2016) panel. The HRC panel was phased using ShapeIt v2, and the imputation was performed using the Michigan imputation server. This gave 8237 eligible children with available genotype data after exclusion of related subjects using cryptic relatedness measures described previously.

### Heritability analyses

For the SNP heritability analyses, imputed genotypes were converted to “best-guess” genotypes, meaning the genotype call most likely to be true given the imputation dosages, using PLINK with a “hard-call” threshold of 0.499. In PLINK, duplicate SNPs were removed, as well as SNPs with Hardy-Weinberg Equilibrium (HWE) *P* < 1 × 10^−6^ and MAF < 2.5 × 10^−5^. Additionally, PLINK was used to perform a second round of filtering of SNPs with a missingness rate > 5% and individuals missing genotypes at > 5% of SNPs. One of each pair of individuals with an estimated genetic relationship of > 0.025 was removed to reduce bias from cryptic relatedness.

Genetic restricted maximum likelihood (GREML) [97] was used to calculate the amount of trait variance explained by genotyped and imputed SNPs. For the cross-sectional analyses, *Z*-scores for all phenotypes were adjusted for height-for-age *Z*-score, with the exception of height and skull, which were not adjusted for height *Z*-scores. *Z*-scores were further adjusted for age, sex, cohort (longitudinal set or cross-sectional set), collection site (one of five clinical sites), dietary calcium intake, physical activity [98], and the first 10 genetic PCs to adjust for population substructure. For the SITAR parameters *a-size*, *b-timing*, and *c-velocity*, GCTA analysis was performed while adjusting for study site and the first 10 genetic PCs, the only covariates that did not change over time. Sensitivity analyses to examine the effect of ancestry were performed modeling ancestral groups together, as well as with the addition and removal of 10 PCs as covariates.

### Genetic correlation across skeletal sites

We performed GCTA bivariate GREML analysis [97] for cross-sectional phenotypes to estimate the amount of genetic covariance (the genome-wide effect of causal variants that affect multiple traits) between skeletal sites and aBMD and BMC at each individual skeletal site. PhenoSpD [19] was used to run LD Score Regression genetic correlation analysis [99] of longitudinal phenotypes. Power calculations for the cross-sectional, longitudinal, and genetic correlation estimates can be found in Additional file 1: Table S2. Most cross-sectional genetic correlation comparisons resulted in high SE estimates, reflecting a large variation surrounding the point estimates and thus low degree of confidence in their accuracy; however, taking only estimates with relatively small SE (< 0.10), i.e., those that were most precise, all genetic correlations were high (> 0.7) and passed a Bonferroni significance threshold adjusting for the number of comparisons (0.05/78 = 0.00064).

### Genome-wide association analysis

In the BMDCS, GWAS was performed for a total of 36 models: the 6 skeletal sites noted above, for each of the 3 SITAR parameters (*a-size*, *b-timing*, *c-velocity*), for both aBMD and BMC. GEMMA [100] was used to create centered relationship matrices, and mixed effects models (Wald test) were run on sex- and ancestry-standardized SITAR parameters adjusted for collection site (max *N* with phenotypes, genotypes, and covariates = 1362). The results were subsequently filtered for MAF < 0.05, HWE *P* < 1 × 10^−6^, and imputation quality (INFO) > 0.4.

### Replication

In ALSPAC, we performed GWAS using a linear mixed model in BOLT-LMM v.2.3 [101]. This model estimates heritability parameters and the infinitesimal mixed model association statistics. We also included the first 2 principal components. Genotype data were inputted in PLINK binary format. We used a reference map from BOLT-LMM (build hg19) to interpolate genetic map coordinates from SNP physical (base pair) positions. Reference LD scores supplied by BOLT-LMM and appropriate for analyses of European-ancestry samples were used to calibrate the BOLT-LMM statistic. LD scores were matched to SNPs by base pair coordinate.

We extracted all lead SNPs at our 40 prioritized loci and their proxies (*r*^2^ > 0.8 based on a European reference using SNiPA, https://snipa.helmholtz-muenchen.de/snipa3/) from the ALSPAC GWAS results and looked for broad support at *P* < 0.05.

### Functional candidate gene annotation

We extracted all genes and transcripts in the TADs surrounding each sentinel SNP using the TAD pathways pipeline [34]. The protein-coding genes were then functionally annotated for GO terms, KEGG pathways, and OMIM disease association using the Database for Annotation, Visualization and Integrated Discovery (DAVID) v. 6.8 (https://david.ncifcrf.gov/). To search for pan-tissue eQTLs, we extracted all SNPs in LD (*r*^2^ > 0.8) with our sentinel SNPs. These SNPs were then queried for significant variant-gene eQTLs in all tissues in GTEx v.7 [41]. We refer to these as “LD-eQTLs” since no colocalization analysis was performed. To search for enriched pathways, all genes and transcripts were subjected to TAD pathway analysis [34].

### ATAC-seq and high-resolution promoter-focused capture C

The ATAC-seq and capture C methods have been previously published [15]. Briefly, we first identified all proxy SNPs in LD (*r*^2^ = 0.5) with the sentinel GWAS SNPs using raggr (www.raggr.usc.edu) with the following parameters: populations: CEU + FIN+GBR + IBS + TSI; min MAF = 0.001; min *r*^2^ = 0.5; max *r*^2^ = 1.0; max distance = 500 kb; max Mendelian errors = 1; HWP cutoff = 0; min genotype % = 75; genotype database = 1000 Genomes Phase 3; genome build hg19. We then assessed which of these proxy SNPs and which of the gene promoters baited in our capture C library resided in an open chromatin region in hMSC-derived osteoblasts, by intersecting their genomic positions with those of the ATAC-seq peaks (using the BEDTools function intersectBed with 1 bp overlap). Next, we extracted the chromatin loops linking open proxy SNPs and open gene promoters in the hMSC-derived osteoblast capture C dataset (bait-to-bait interactions were excluded). The results were visualized using the UCSC Genome Browser.

### Functional assays in hMSCs

Primary bone-marrow derived hMSCs isolated from healthy donors (age range 22–29 years) were characterized for cell surface expression (CD166 + CD90 + CD105+/CD36-CD34-CD10-CD11b-CD45-) and tri-lineage differentiation (osteoblastic, adipogenic, and chondrogenic) potential at the Institute of Regenerative Medicine, Texas A&M University. Expansion and maintenance of the cells were carried out using alpha-MEM supplemented with 16.5% fetal bovine serum (FBS) in standard culture conditions by plating cells at a density of 3000 cells/cm^2^.

Experimental knockdown of candidate genes was achieved using DharmaFECT1 transfection reagent (Dharmacon Inc., Lafayette, CO) using sets of 4 ON-TARGETplus siRNAs in three temporally separated independent hMSC donor lines. Following siRNA transfection, the cells were allowed to recover for 2 days in maintenance media and stimulated with BMP2 for additional 3 days in serum-free osteogenic media as previously described [15]. To assess the influence of knockdown on gene expression (RT-qPCR) and early osteoblast differentiation (histochemical ALP staining), cells were harvested at 3 days post BMP2 stimulation. For extracellular matrix calcium deposition, cells were stained with 0.1% Alizarin Red S after 8–10 days post-BMP2 stimulation. Details of the siRNA and RT-qPCR primers are provided in Additional file 1: Tables S19-S21.

For quantification of histochemical ALP stain and Alizarin Red staining, multi-well plates were allowed to air-dry and each well was scanned using high-resolution color bright field objective (1.25X) of the Lionheart FX automated microscope (BioTek). For each scanned well, image analysis was performed using Image J software according to the guidelines provided by the National Institute of Health. For histochemical assays, two independent experiments per siRNA per donor line were performed. *P* values for differences between groups were calculated using two-way homoscedastic Student’s *t* tests.

### Functional assays in hFOBs

hFOB1.19 cells were purchased from ATCC (CRL-11372) and grown in a 1:1 mixture of Ham’s F12 Medium and Dulbecco’s Modified Eagle’s Medium containing no phenol red and supplemented with 10% FBS and 0.3 mg/mL G418. Cells were maintained at 33.5 °C using standard culture conditions and differentiated into mature osteoblasts at 39.5 °C for all experiments. Knockdown of candidate genes using siRNA and histochemical ALP staining were performed using the same conditions used for the hMSC donor lines. CRISPR-Cas9 mediated deletion of the *PRPF38A* proxy SNP (rs34455069) and sentinel GWAS SNP (rs2762826) were achieved using a pooled lentiviral mCherry construct (Addgene, 99154) containing three sgRNAs on each side of the target. Lentiviral infection was confirmed using mCherry/Texas Red microscopy and efficiency was calculated using the Countess II FL (Thermo). SNP deletions were confirmed using multiplexed sequencing of PCR products generated from hFOB1.19 DNA across the CRISPR region for both SNPs (Additional file 2: Fig. S12). Quantification of ALP staining in hFOB1.19 cells was similar to that used for hMSC donor lines; however, three technical replicates were used. The plates were photographed, images converted to grayscale, and analyzed using Image J software.

## Supplementary Information

**Additional file 1: Supplementary Tables**. **Table S1**. Cohort and study visit details (BMDCS). **Table S2**. Power calculations for heritability analyses. **Table S3**. Cross-sectional SNP-heritability. **Table S4**. Ancestry-specific sensitivity analyses. **Table S5**. Longitudinal SNP-heritability. **Table S6**. Genetic correlations (baseline). **Table S7**. Genetic correlations (longitudinal). **Table S8**. Association results by sex and ancestry. **Table S9**. Replication in ALSPAC. **Table S10**. Lookup in UK BioBank GWAS. **Table S11**. Fracture outcome lookup in PheWEB. **Table S12**. Topological Associated Domain (TAD) genes. **Table S13**. DAVID functional annotations for protein-coding TAD genes. **Table S14**. TAD pathways: GO terms. **Table S15**. TAD pathways: KEGG terms. **Table S16**. hMSC-derived osteoblast promoter-focused Capture C loops. **Table S17**. Pan-tissue eQTLs. **Table S18**. IL17 Th17 cell promoter-focused Capture C loops. **Table S19**. hMSC donor line information. **Table S20**. siRNA details. Table S21. RT-qPCR primers.

**Additional file 2: Supplementary Figures**. **Fig. S1**. SITAR modeled mean curves for aBMD and BMC by sex and ancestry. **Fig. S2**. Phenotypic and genetic correlation plots. **Fig. S3**. Manhattan and QQ plots. **Fig. S4**. Overlap of signals (A) among skeletal sites and (B) SITAR parameters. **Fig. S5**. TET2 locus. **Fig. S6**. Flow chart showing choice of loci for functional follow-up. **Fig. S7**. Locus zoom regional plots, ancestry and sex-specific association results, and SITAR curves by genotype for (A) signal 17 and (B) signal S6, and (C) Locuszoom plots with African American LD background for the three signals.. **Fig. S8**. Gene expression after siRNA knockdown before and after BMP2 treatment. **Fig. S9**. Staining results in hFOBs. **Fig. S10**. Gene expression of BMP2 signaling markers and osteoblast markers. **Fig. S11**. Gene expression of chondrogenic and adipogenic markers in hMSC-osteoblasts. **Fig. S12**. CRISPR-Cas9 deletion of sentinel SNP at PRPF38A locus. **Fig. S13**. rs34455069 is predicted to disrupt two transcription factor binding sites.. **Fig. S14**. SITAR mean curves in ALSPAC.

**Additional file 3.** Review history.

## Data Availability

BMDCS data can be applied for and downloaded via the NIH Data and Specimen Hub (https://dash.nichd.nih.gov/) [16]. ALSPAC data is also available on request at http://www.bristol.ac.uk/alspac/researchers/access/ [93]. The code used to perform SITAR longitudinal modeling is based on the publicly available R package “sitar,” for which documentation can be found at https://cran.r-project.org/web/packages/sitar/sitar.pdf [102].
