# Supplementary material for: Genome-wide association study implicates novel loci and reveals candidate effector genes for longitudinal pediatric bone accrual
Source: Genome Biol. 2021 Jan 4;22:1. doi: 10.1186/s13059-020-02207-9 (PMC7780623; doi:10.1186/s13059-020-02207-9)
Supplement: Supplementary file 2 — Additional file 2: Supplementary Figures. Fig. S1. SITAR modeled mean curves for aBMD and BMC by sex and ancestry. Fig. S2. Phenotypic and genetic correlation plots. Fig. S3. Manhattan and QQ plots. Fig. S4. Overlap of signals (A) among skeletal sites and (B) SITAR parameters. Fig. S5. TET2 locus. Fig. S6. Flow chart showing choice of loci for functional follow-up. Fig. S7. Locus zoom regional plots, ancestry and sex-specific association results, and SITAR curves by genotype for (A) signal 17 and (B) signal S6, and (C) Locuszoom plots with African American LD background for the three signals.. Fig. S8. Gene expression after siRNA knockdown before and after BMP2 treatment. Fig. S9. Staining results in hFOBs. Fig. S10. Gene expression of BMP2 signaling markers and osteoblast markers. Fig. S11. Gene expression of chondrogenic and adipogenic markers in hMSC-osteoblasts. Fig. S12. CRISPR-Cas9 deletion of sentinel SNP at PRPF38A locus. Fig. S13. rs34455069 is predicted to disrupt two transcription factor binding sites.. Fig. S14. SITAR mean curves in ALSPAC. [file 13059_2020_2207_MOESM2_ESM.docx]

**Genome-wide association study implicates novel loci and reveals candidate effector genes for longitudinal pediatric bone accrual through variant-to-gene mapping**

**Additional File 2**

**Figures**

Fig. S1. SITAR modeled mean curves for aBMD and BMC by sex and ancestry

Fig. S2. Phenotypic and genetic correlation plots

Fig. S3. Manhattan and QQ plots

Fig. S4. Overlap of signals (A) among skeletal sites and (B) SITAR parameters

Fig. S5. TET2 locus

Fig. S6. Flow chart showing choice of loci for functional follow-up

Fig. S7. Locus zoom regional plots, ancestry and sex-specific association results, and SITAR curves by genotype for (A) signal 17 and (B) signal S6, and (C) Locuszoom plots with African American LD background for the three signals.

Fig. S8. Gene expression after siRNA knockdown before and after BMP2 treatment

Fig. S9. Staining results in hFOBs

Fig. S10. Gene expression of BMP2 signaling markers and osteoblast markers

Fig. S11. Gene expression of chondrogenic and adipogenic markers in hMSC-osteoblasts

Fig. S12. CRISPR-Cas9 deletion of sentinel SNP at PRPF38A locus

Fig. S13. rs34455069 is predicted to disrupt two transcription factor binding sites.

Fig. S14. SITAR mean curves in ALSPAC.

**Figure S1. Mean SITAR curves by sex and ancestry for the six skeletal sites.
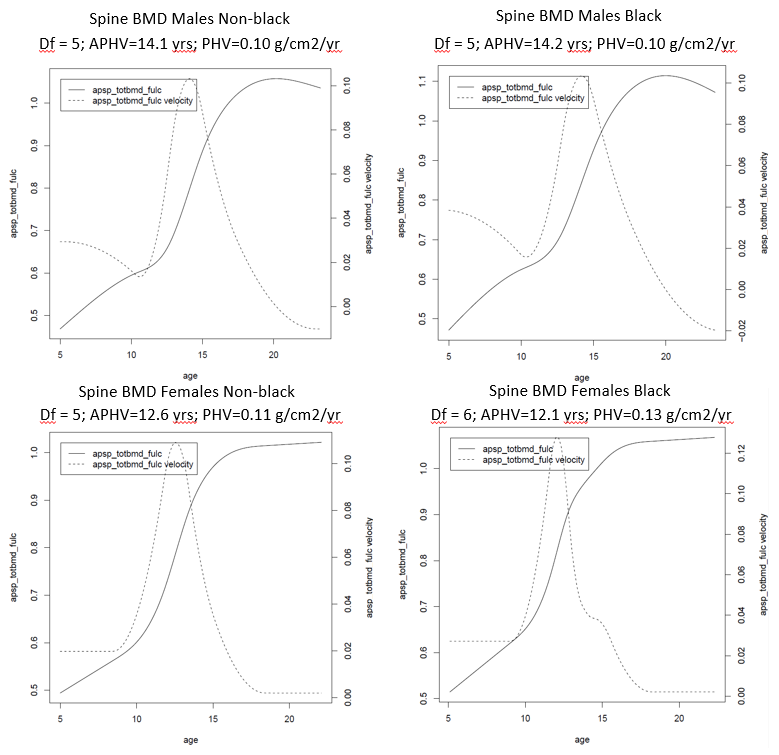

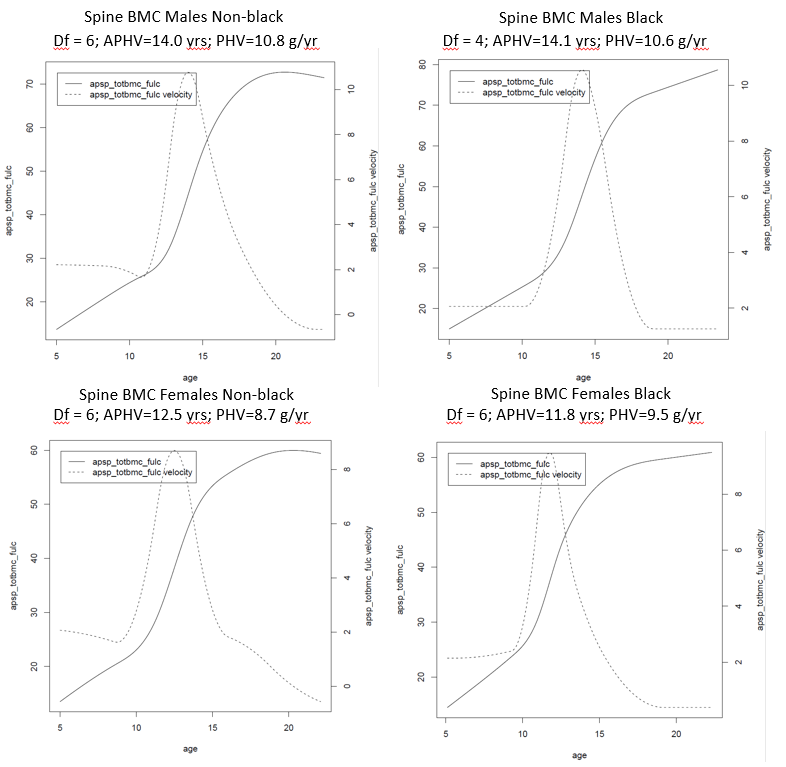
**

**
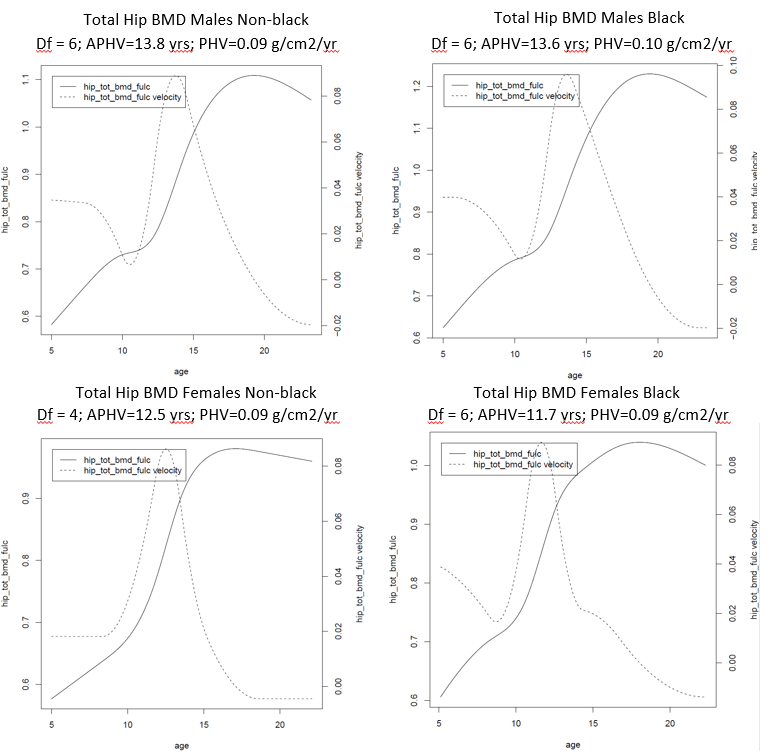
**

**
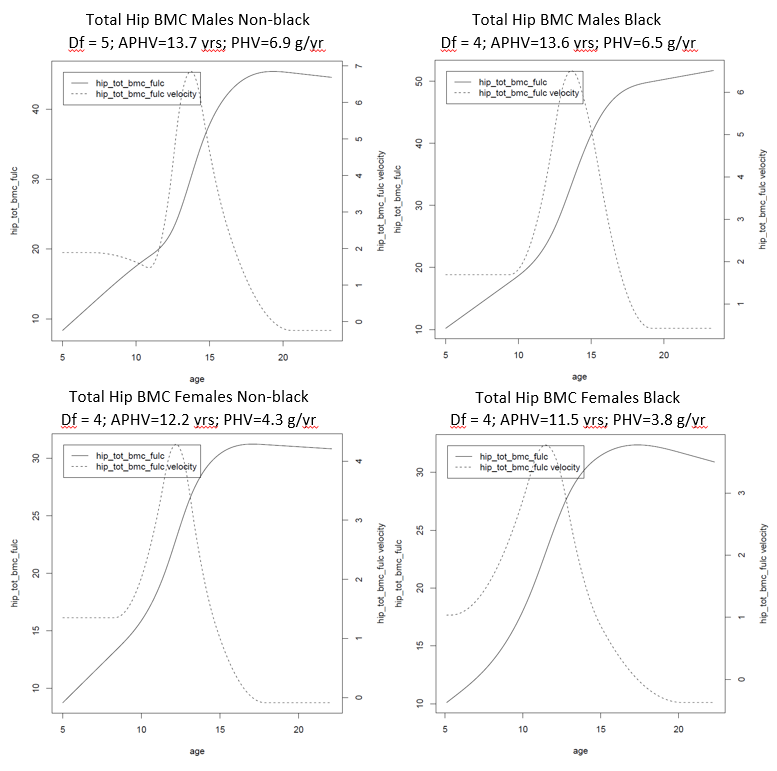
**

**
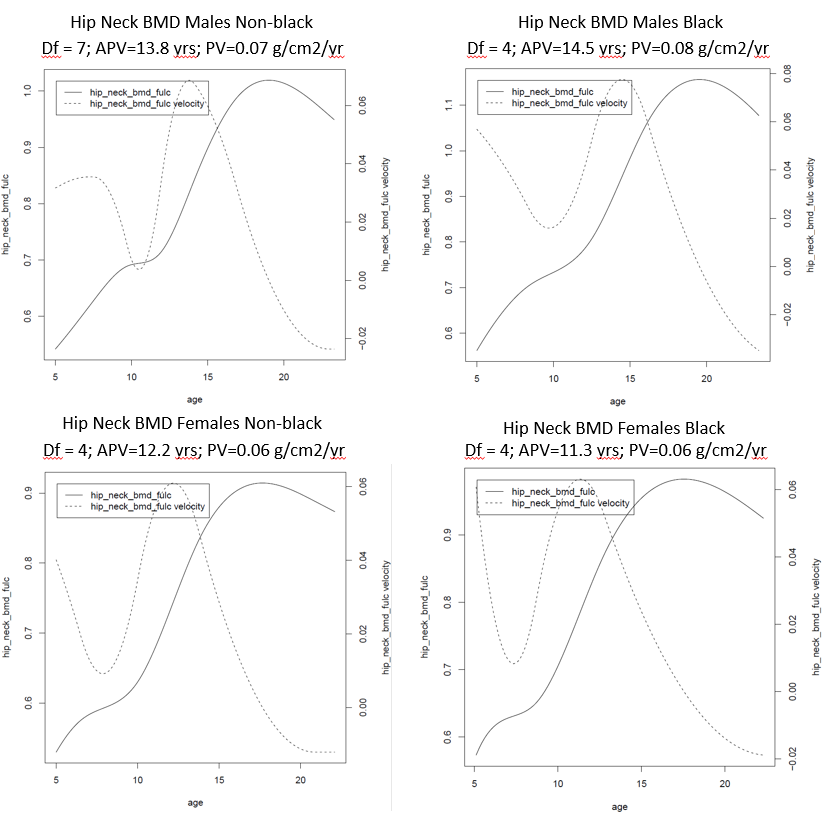
**

**
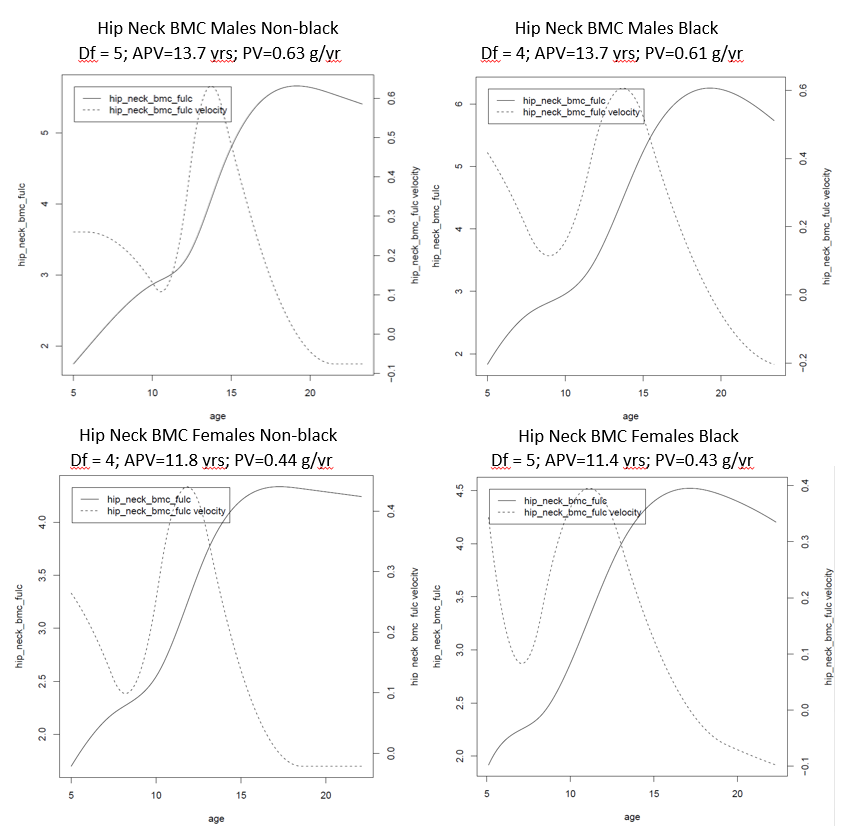
**

**
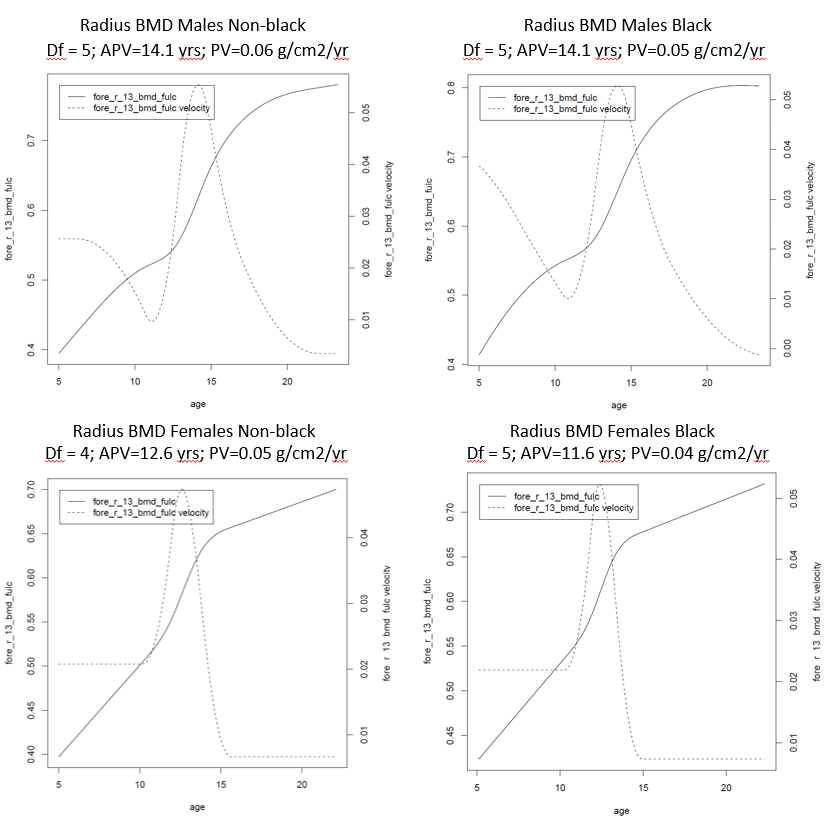
**

**
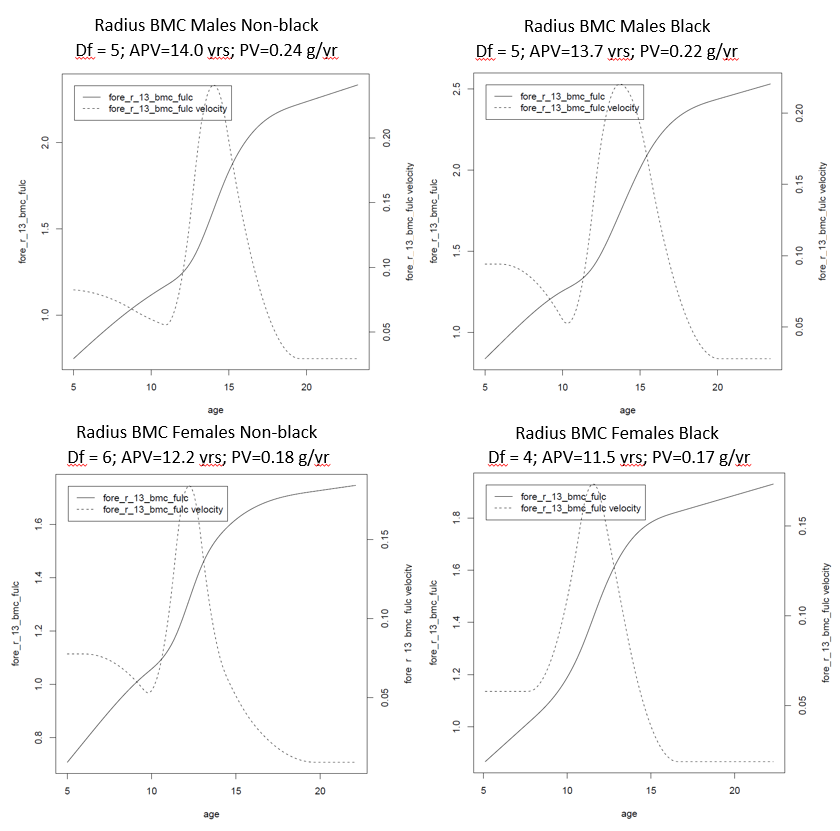
**

**
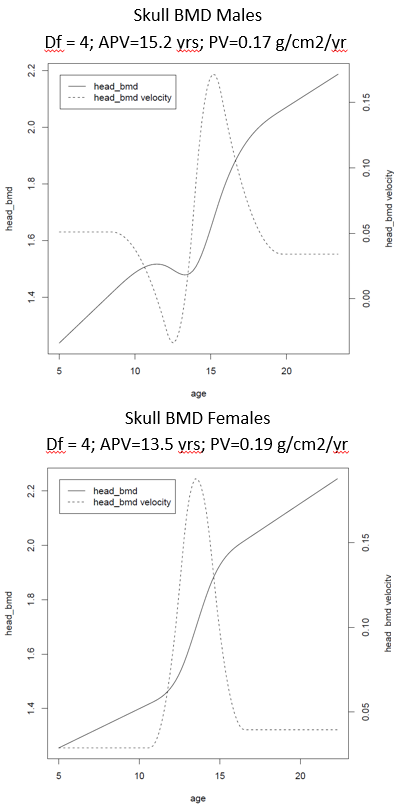
**

**
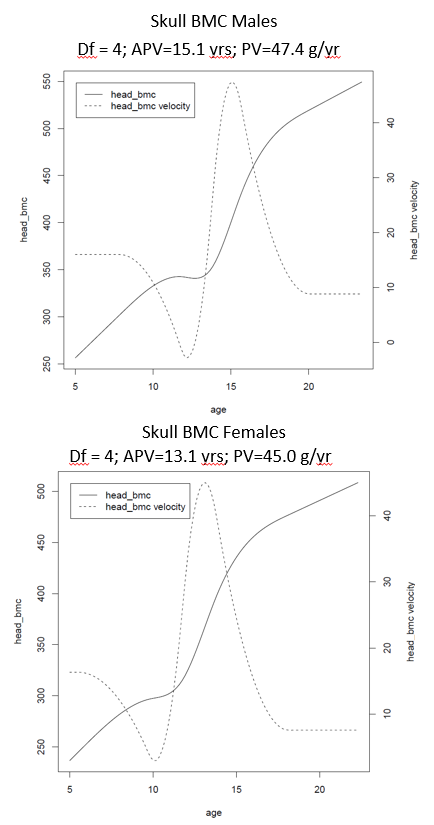
**

**
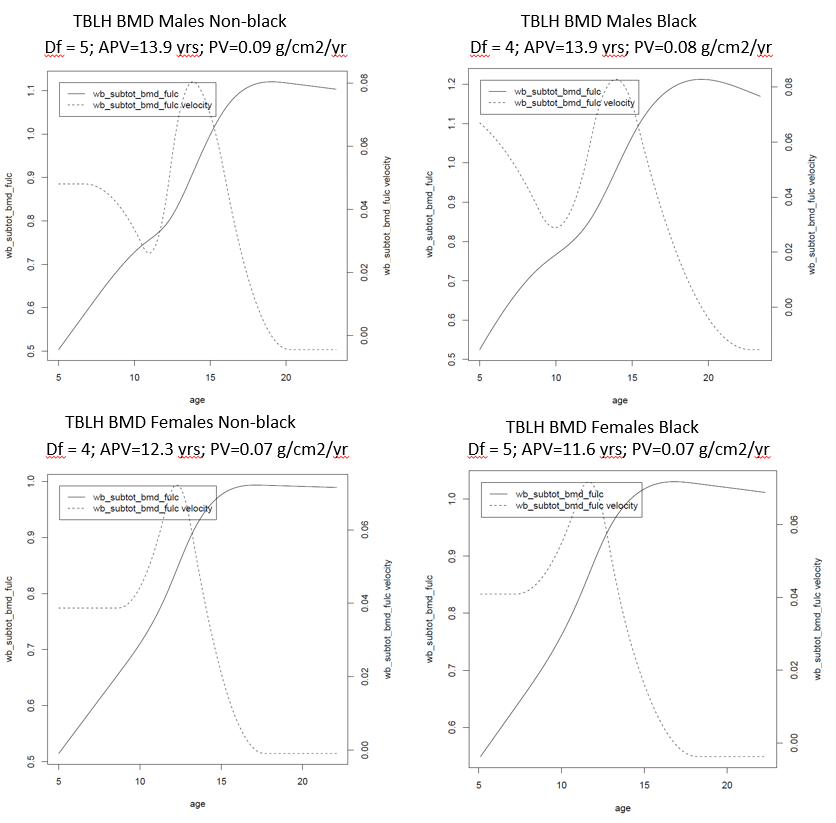
**

**
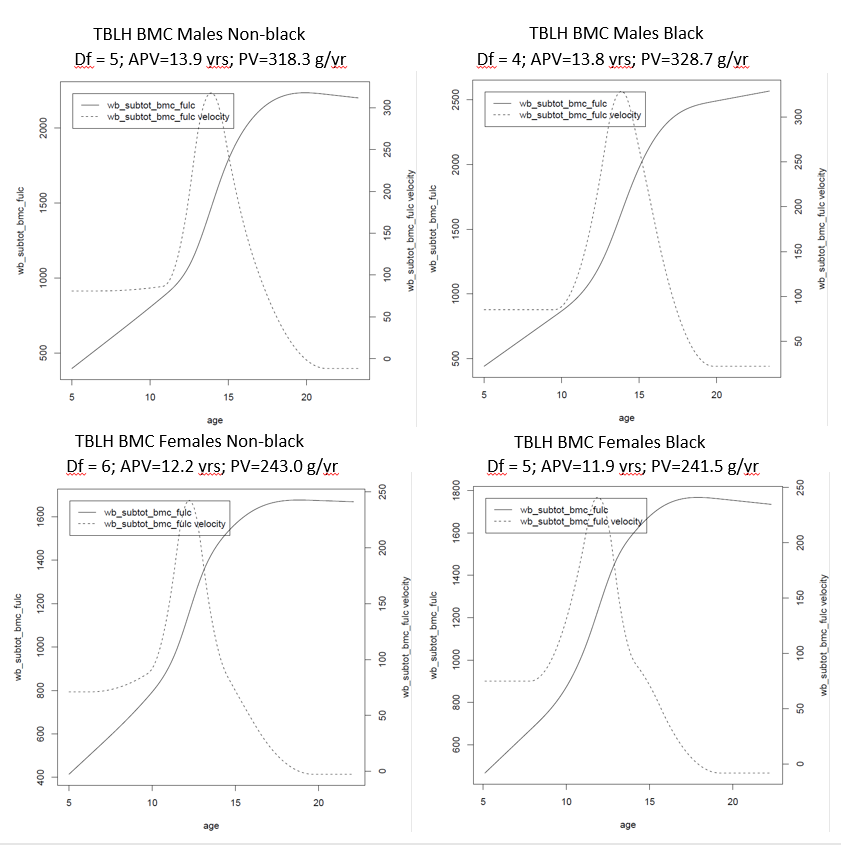
**

**Figure S2. Correlations between skeletal site phenotypes.** (A) Phenotypic correlations, calculated in R. (B) Genetic correlations based on bivariate correlations, calculated in GCTA. (C) Phenotypic correlations between a-size, b-timing, and c-velocity, calculated in R. Genotypic correlations calculated using LD Score Regression in PhenoSpD are shown in Supplementary Table 7.


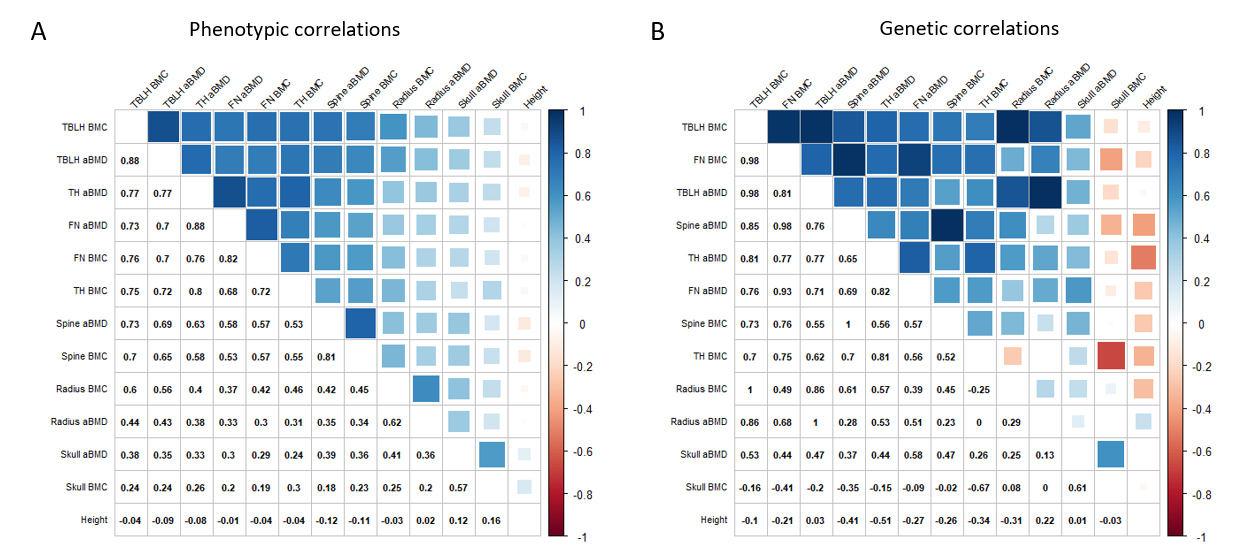

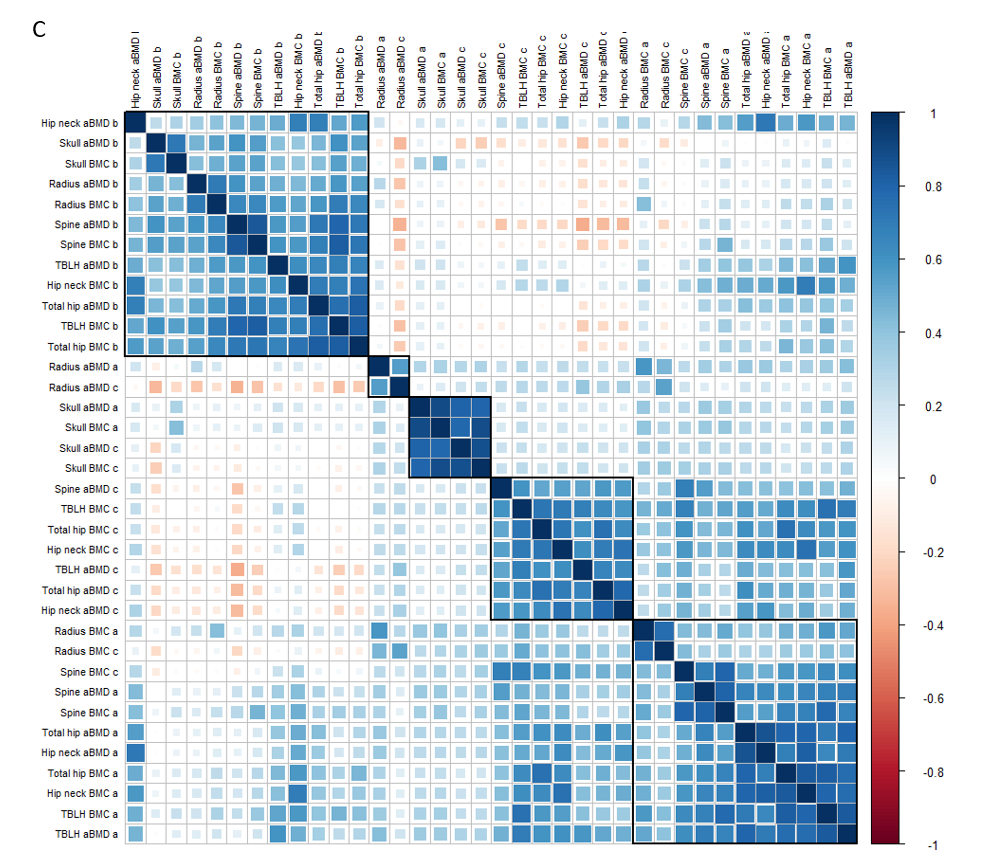


**Figure S3. Manhattan and QQ plots.**

**
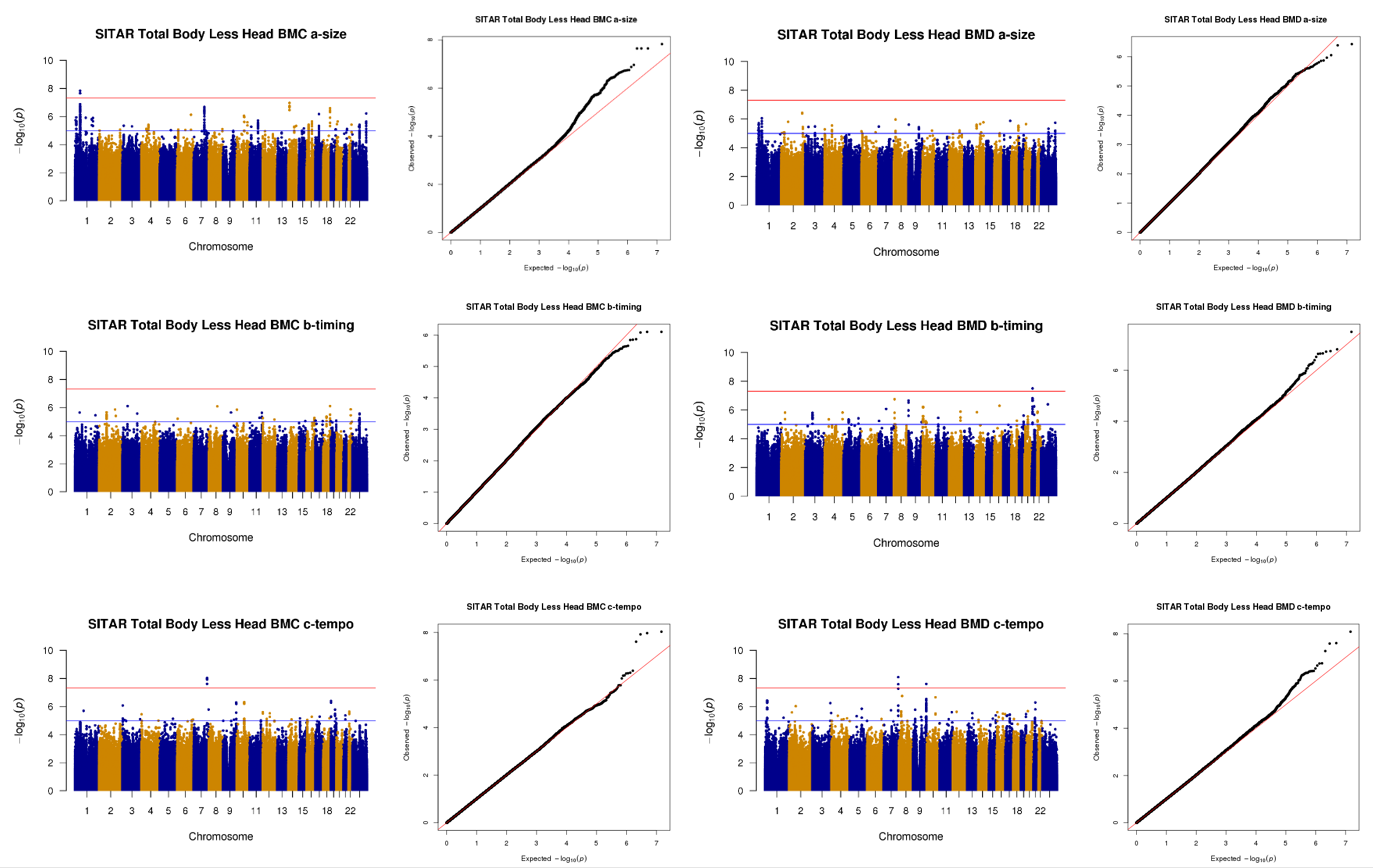


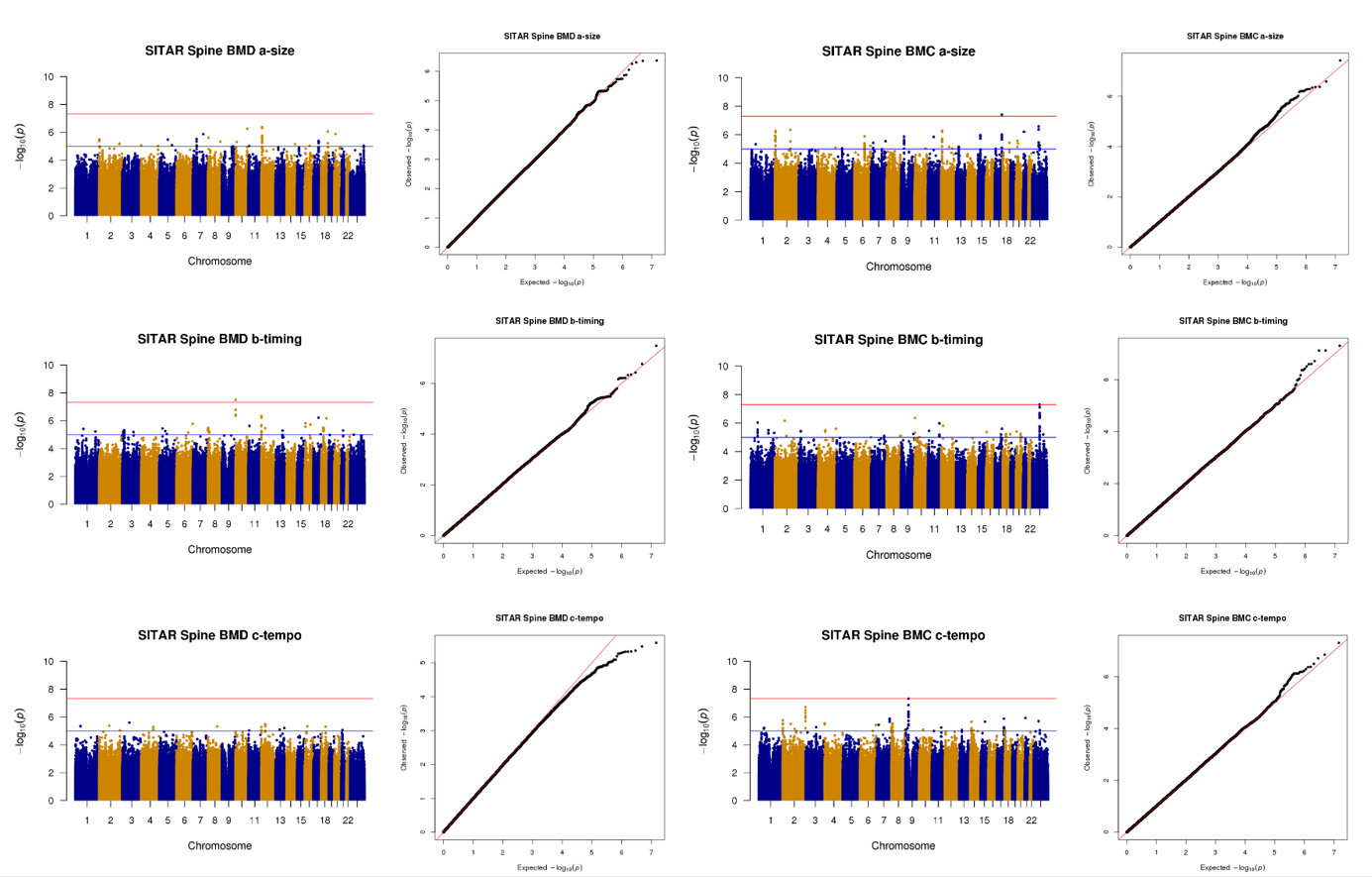
**


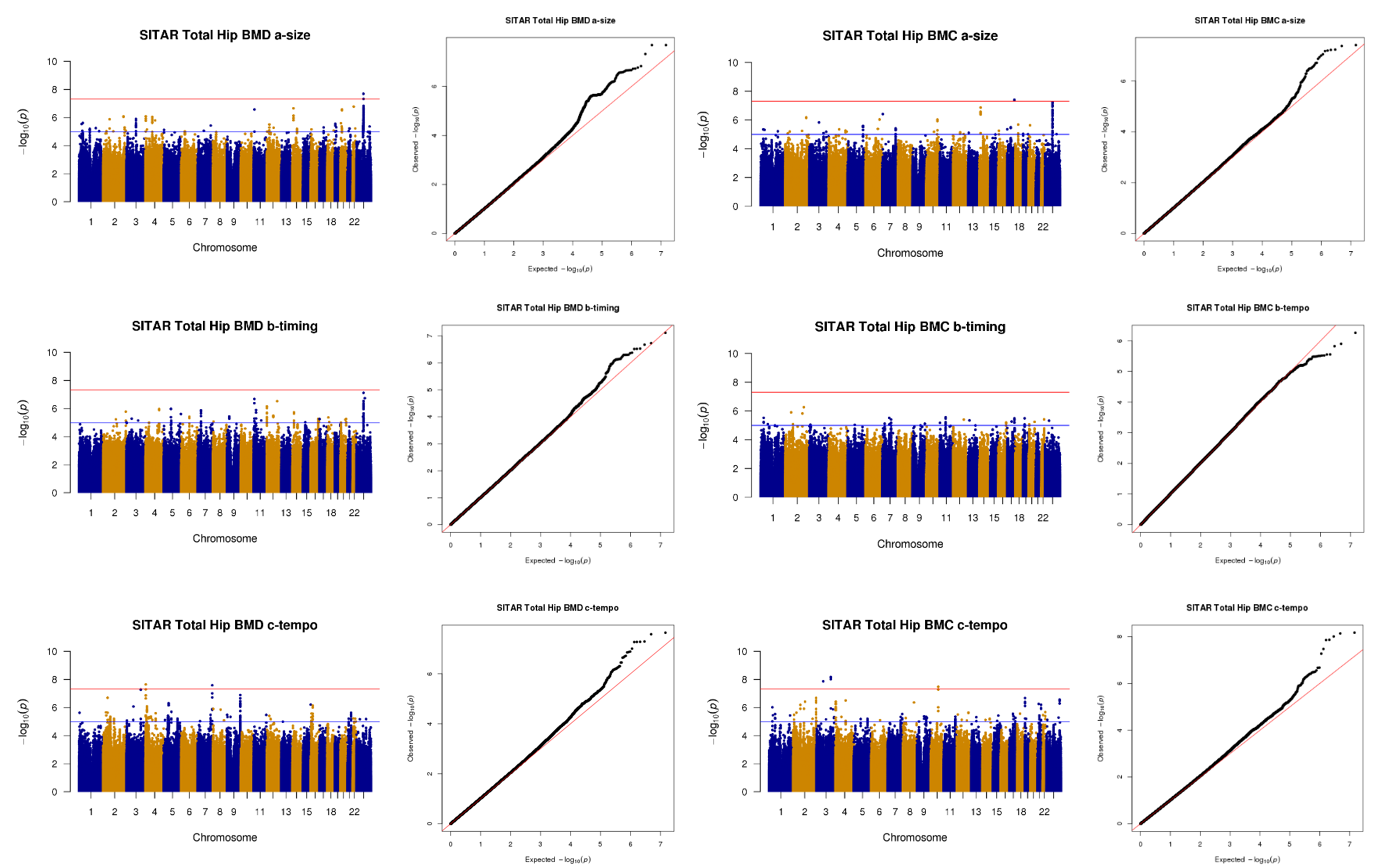

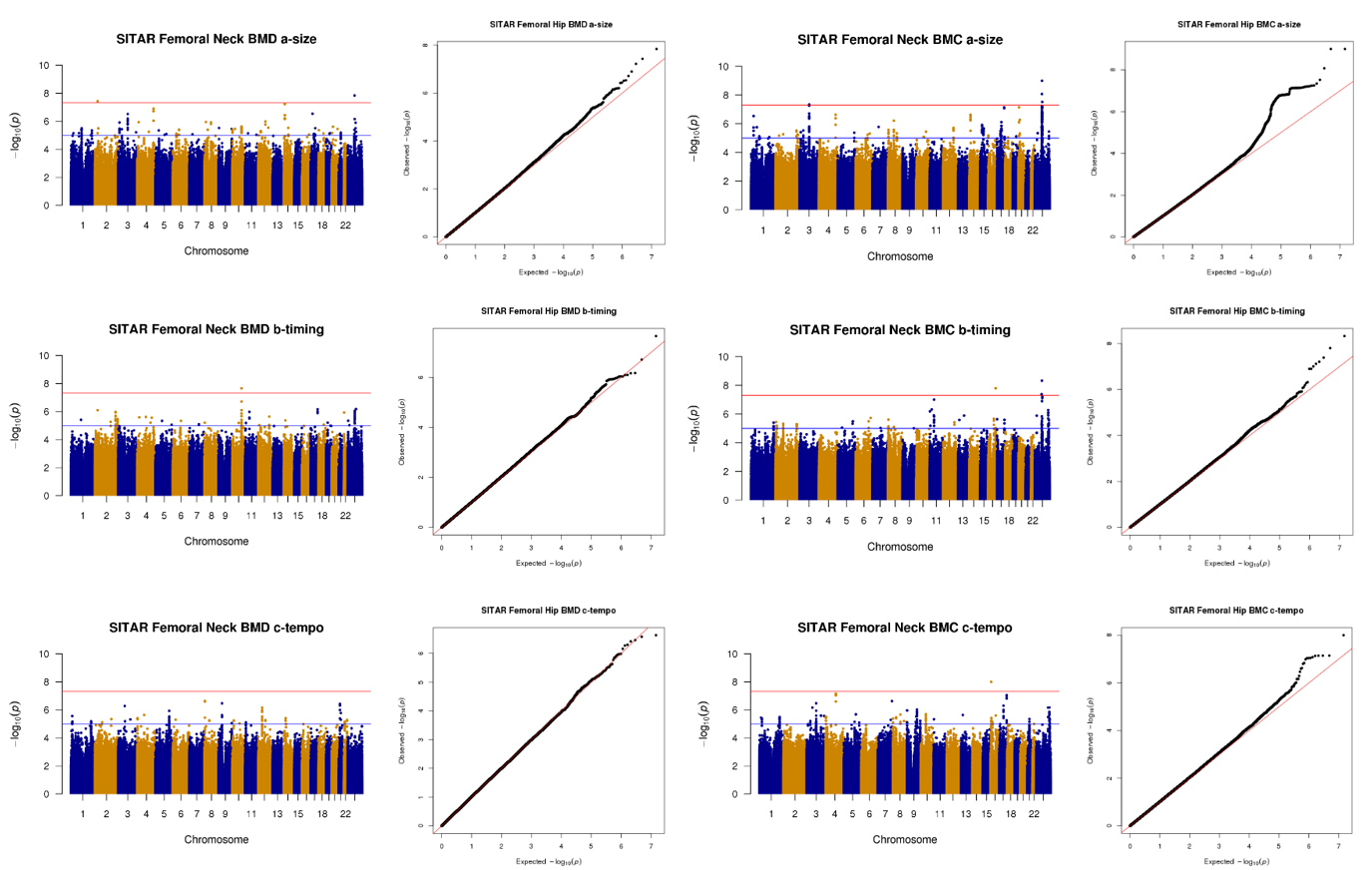

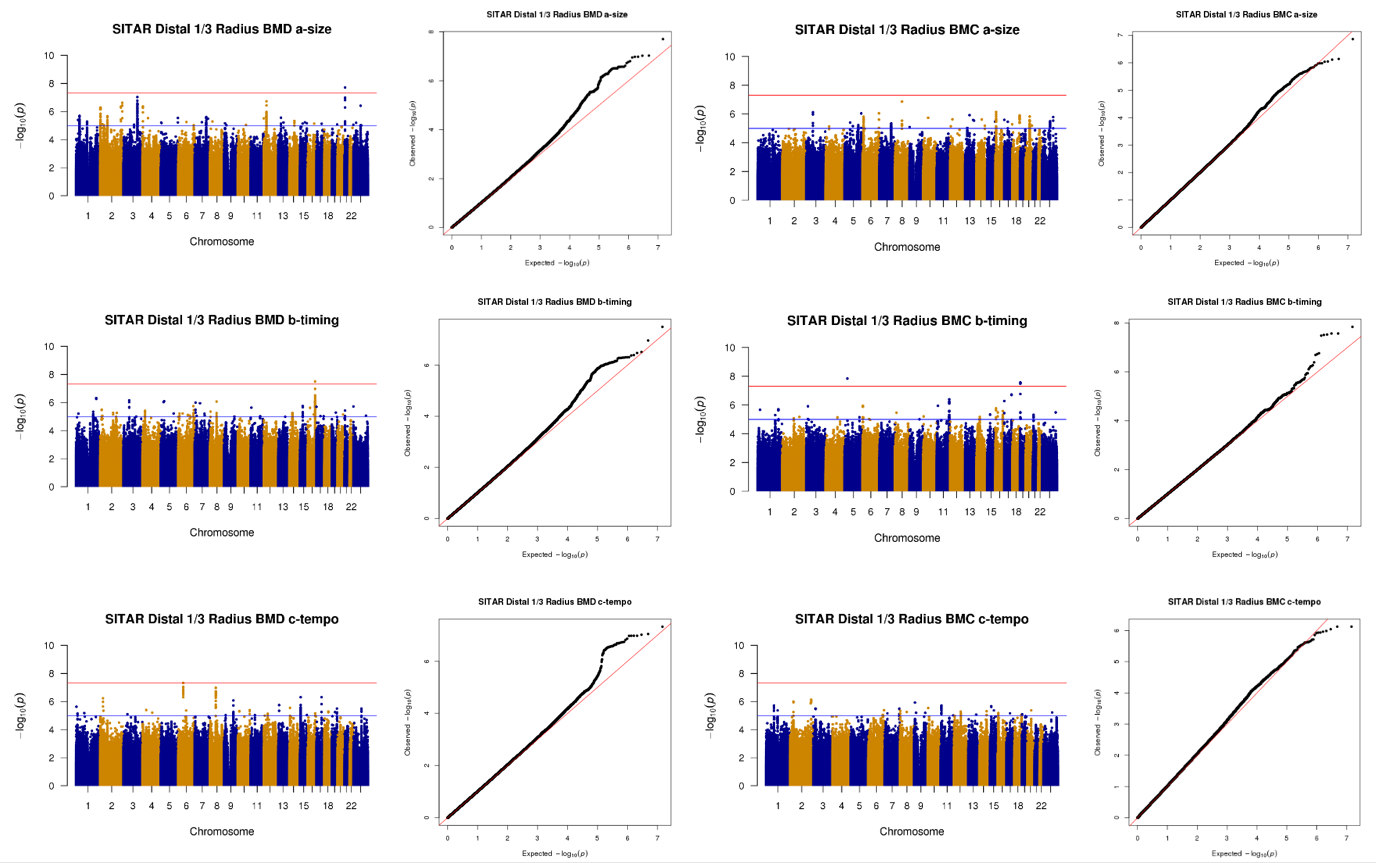

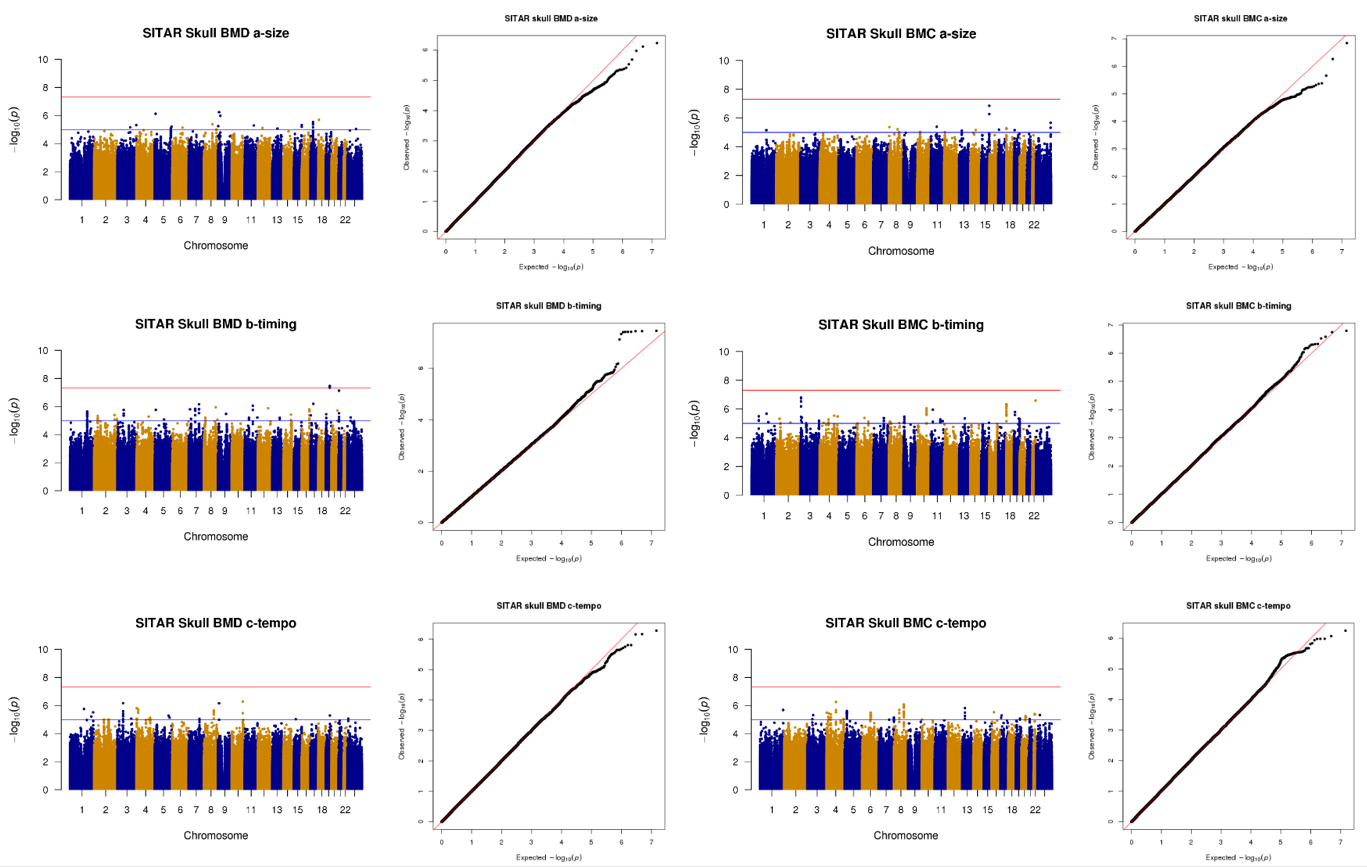


**Figure S4. Overlap of signals (A) among skeletal sites and (B) SITAR parameters.**

**
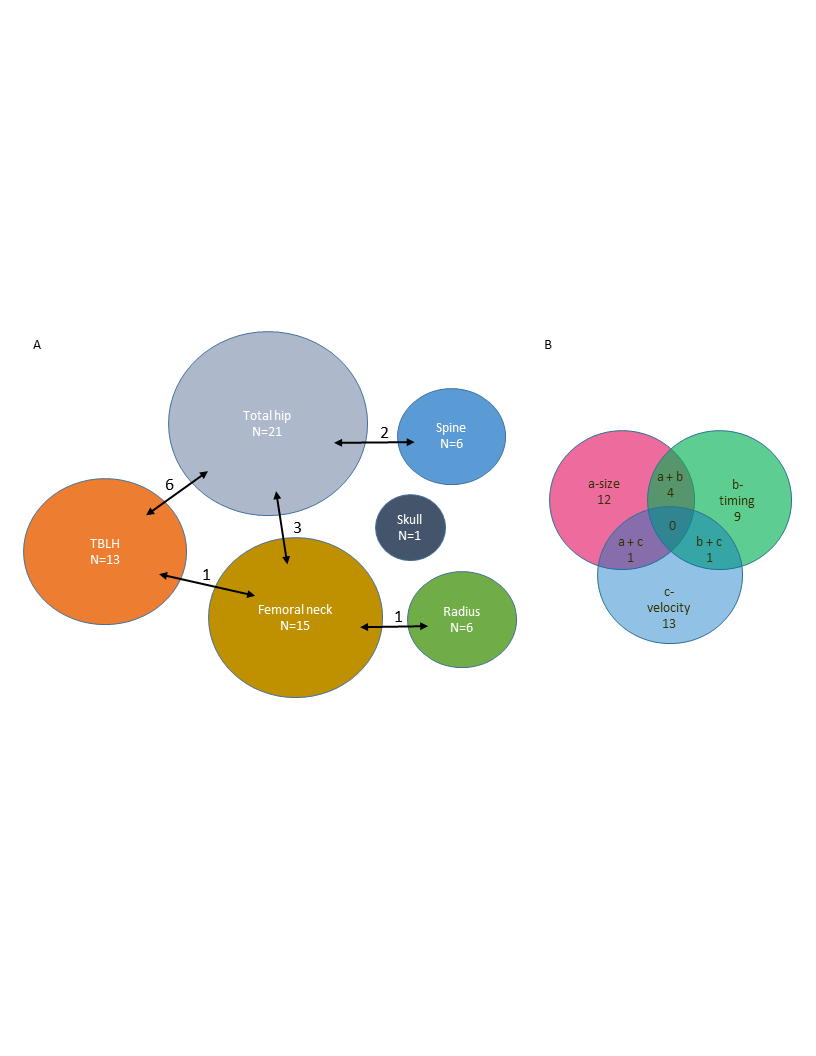
**

**Figure S5. *TET2* locus.** (A) UCSC browser view showing the region upstream of *TET2*. Top track shows open chromatin peaks as assessed by ATAC-seq in hMSC-derived osteoblasts. Yellow lines represent the two sentinel SNPs, rs56883672 and rs6533178, and proxy SNP rs2301718, which shows a *cis* interaction with the promoter of *TET2* in hMSC-derived osteoblasts. (B) Position weight matrix for the RBPj-kappa binding site (below) from RegulomeDB, with rs2301718 in the orange box. (C) UCSC genome browser shot showing the proximity of rs2301718 (blue line) to an upstream FOXO3 binding site.

**
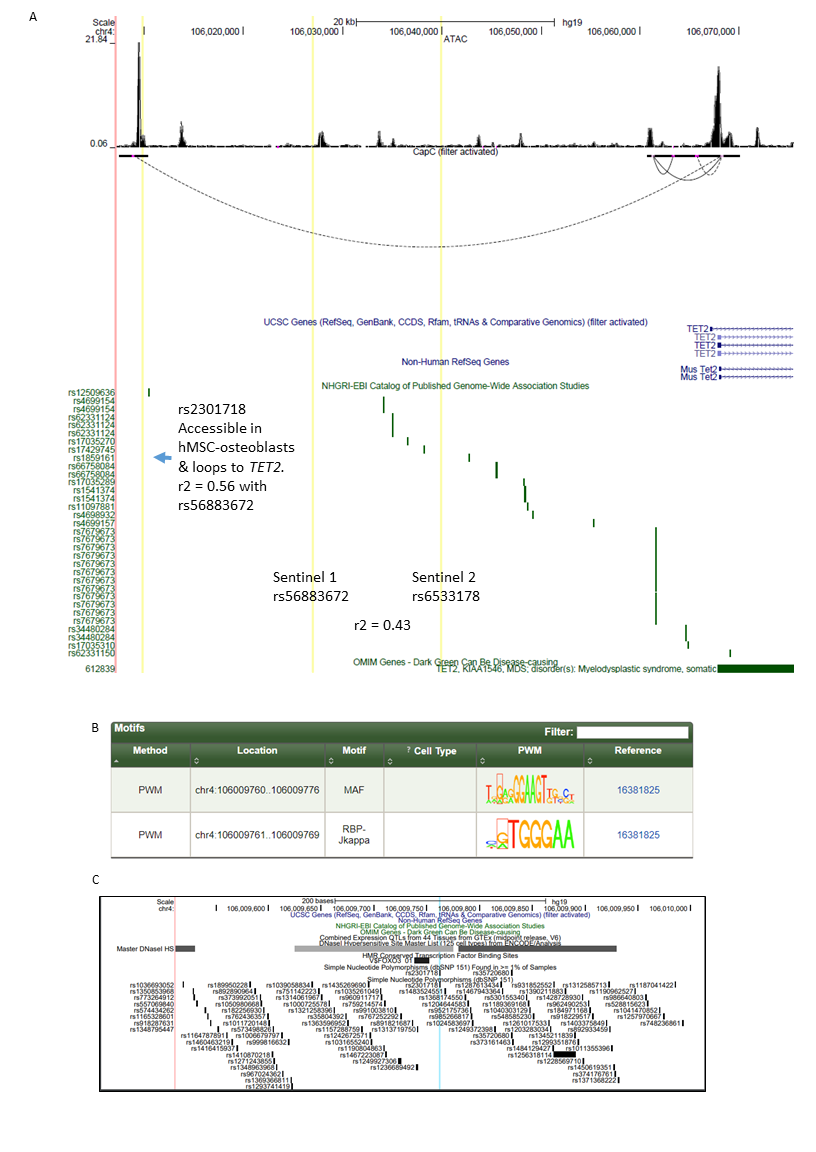
**

**Figure S6. Flow chart showing choice of loci for functional follow-up.**

**
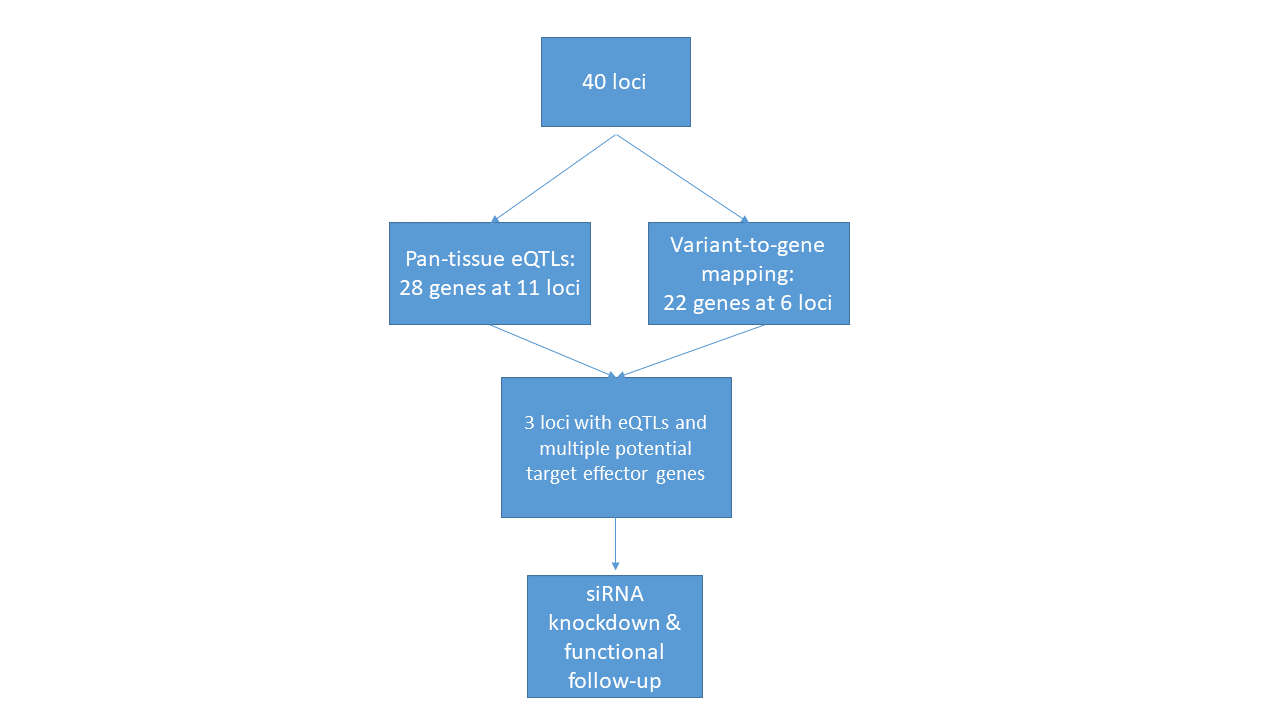
**

**Figure S7. Locus zoom regional plots, ancestry and sex-specific association results, and SITAR curves by genotype for (A) signal 17 and (B) signal S6.**

1. Signal 17, associated with femoral neck BMC b-timing.

**
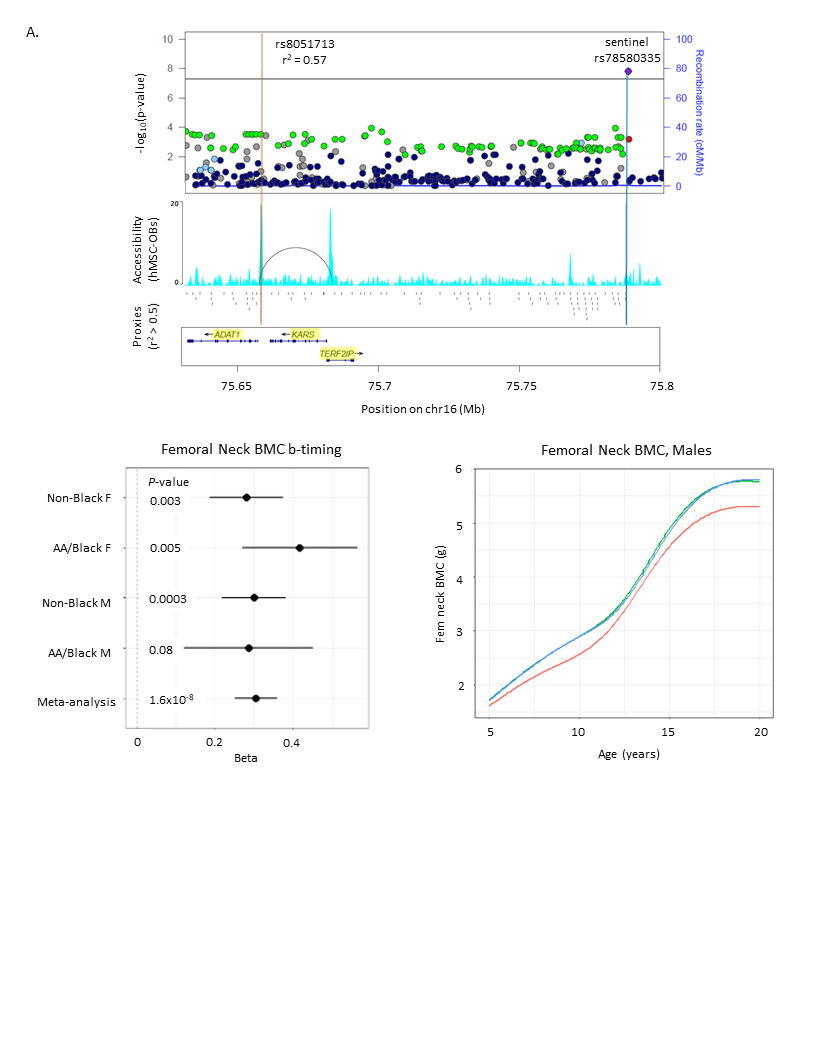
**

1. Signal S6, suggestively associated with total hip BMD b-timing and spine BMD b-timing.

**
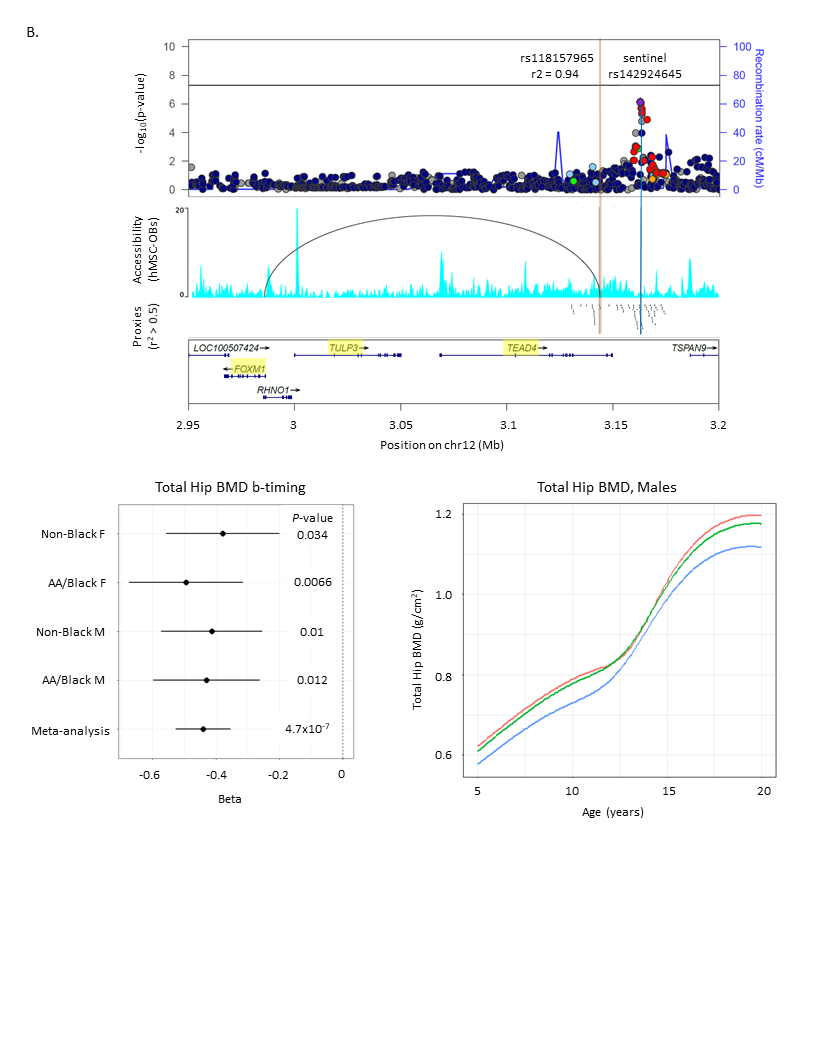
**

1. Locuszoom plots with African American LD background.


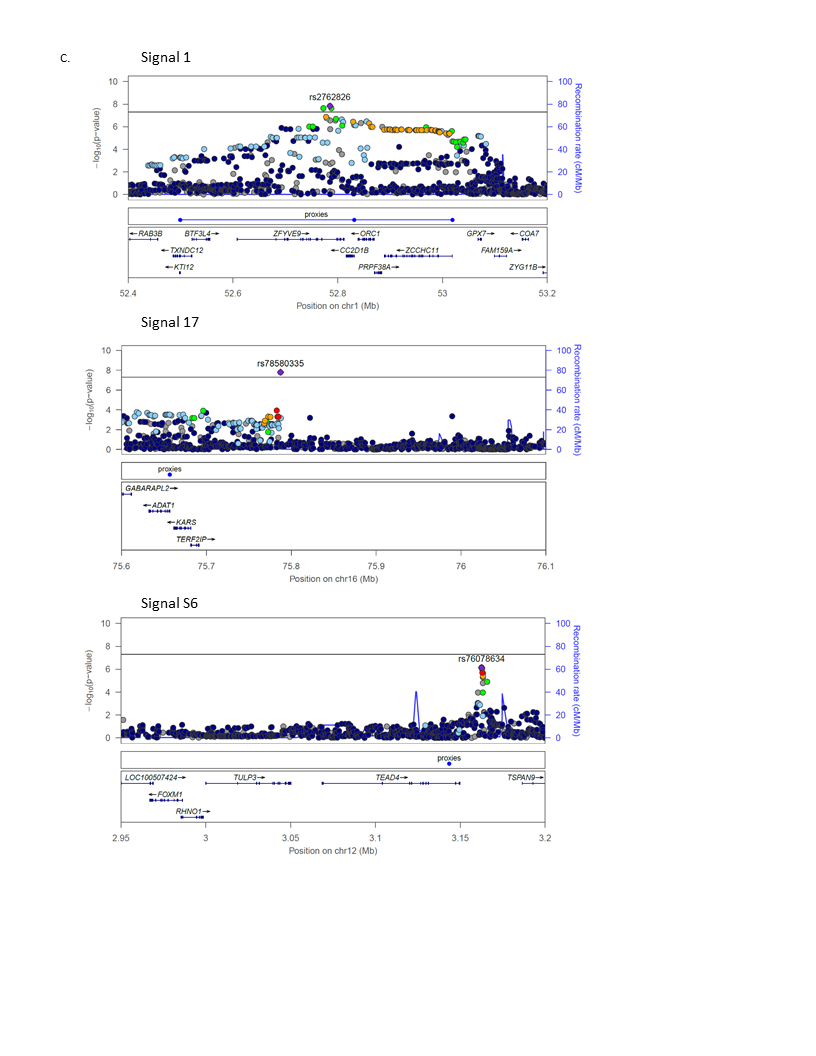


**Figure S8. Gene expression after siRNA knockdown in hMSCs and BMP2-induced osteoblasts as assessed by qPCR.** **p*<0.05 comparing no treatment to BMP2 treatment for each siRNA, *#p*<0.05 comparing control siRNA to siRNA for gene of interest.


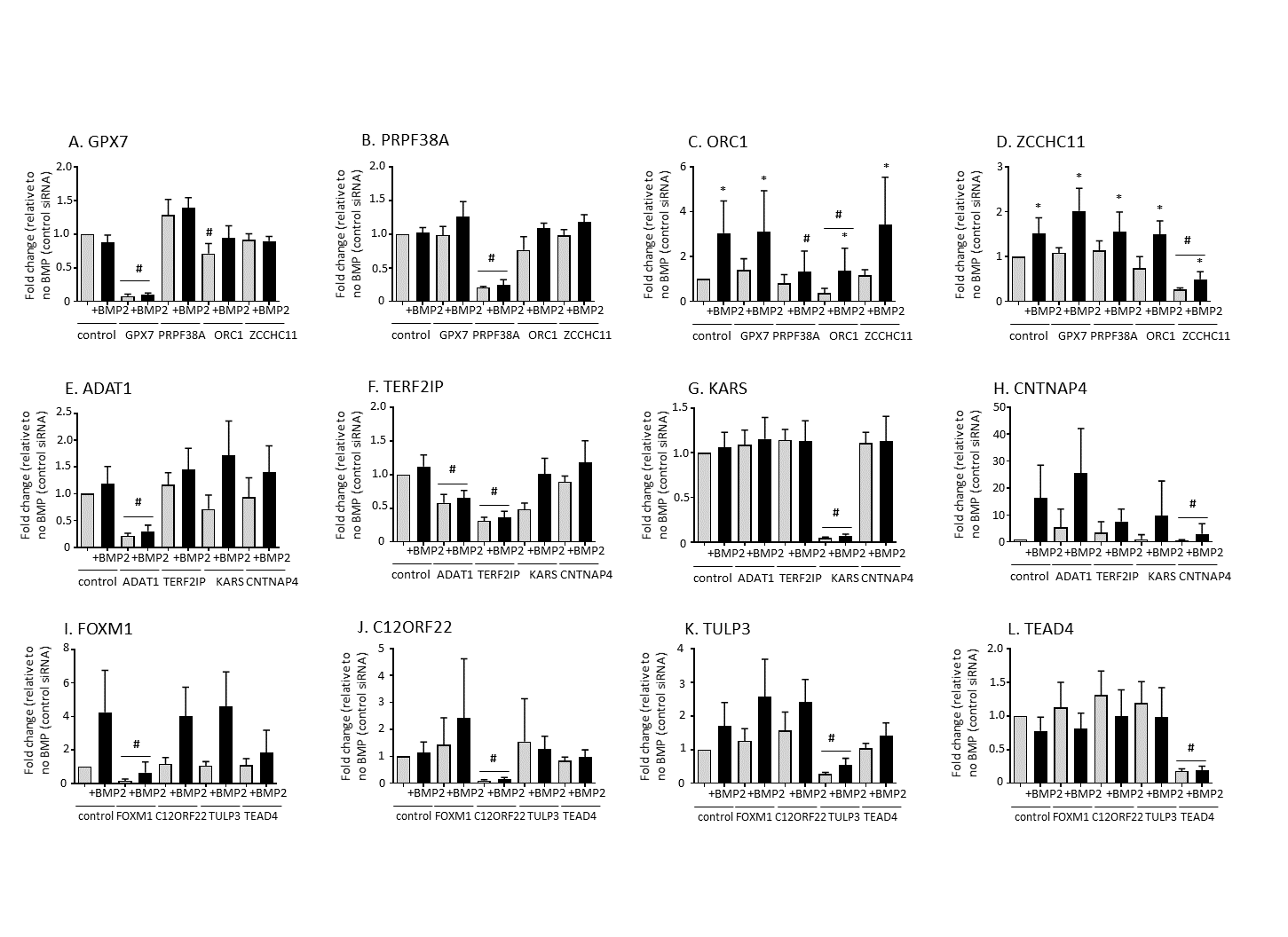


**Figure S9. siRNA knockdown in human fetal osteoblasts (hFOBs).** A) Alkaline phosphatase staining assay in hFOB cells in permissive (33.5°C) and osteoblast differentiation (39.5°C) growth conditions after the addition of *KARS*, *PRPF38A*, and *TEAD4* siRNA. Alkaline phosphatase stain was reduced in all three knockdowns. B) qPCR of gene expression levels in hFOBs under 33.5°C and 39.5°C shows specific knockdown of gene expression by each respective siRNA. **p*<0.05, comparing siRNA treatment between permissive (33.5°C) and differentiation (39.5°C) conditions; #p<0.05, comparing control siRNA to target gene siRNA treatment for *KARS*, *PRPF38A*, and *TEAD4*, respectively.

A.


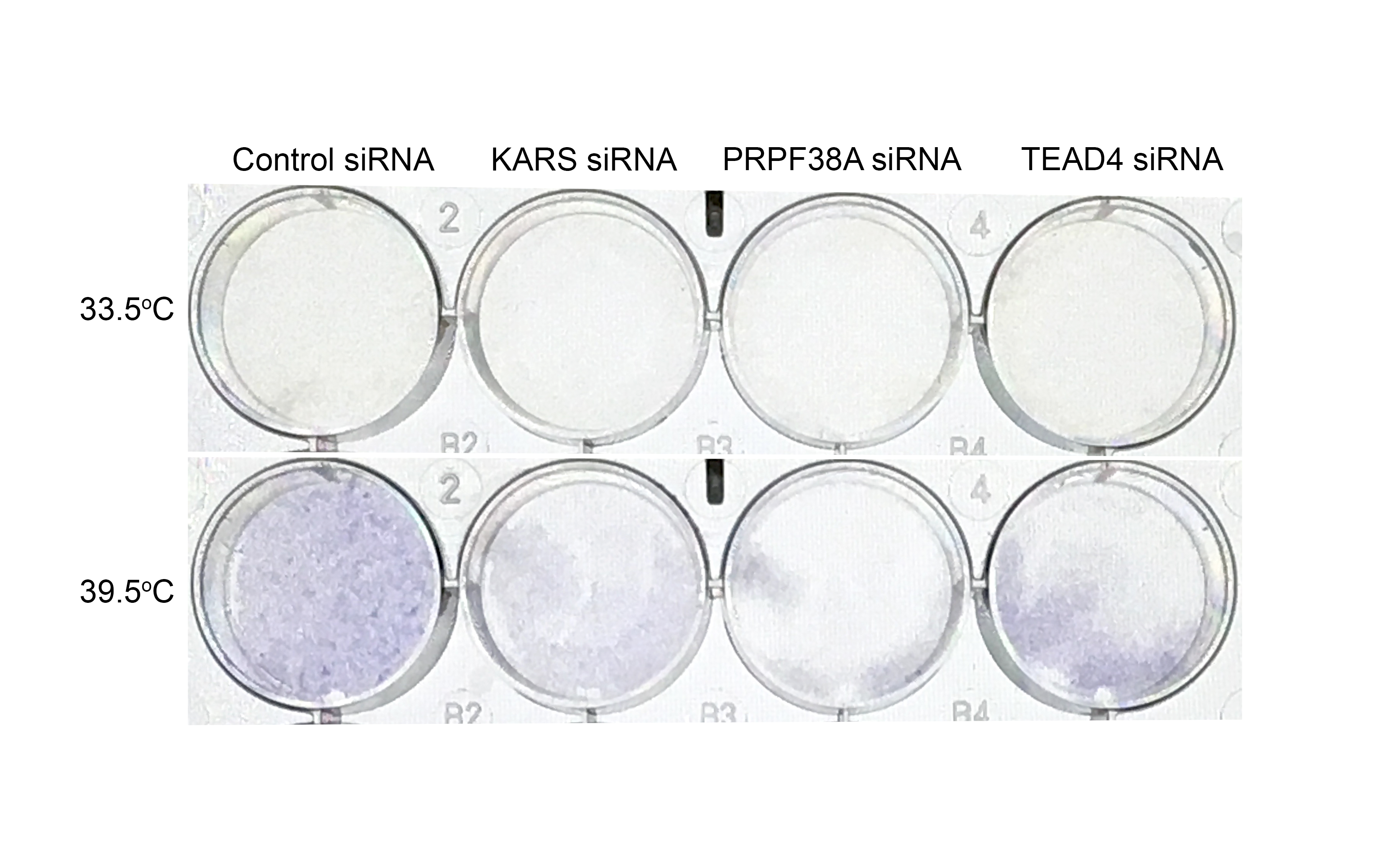


B.

**
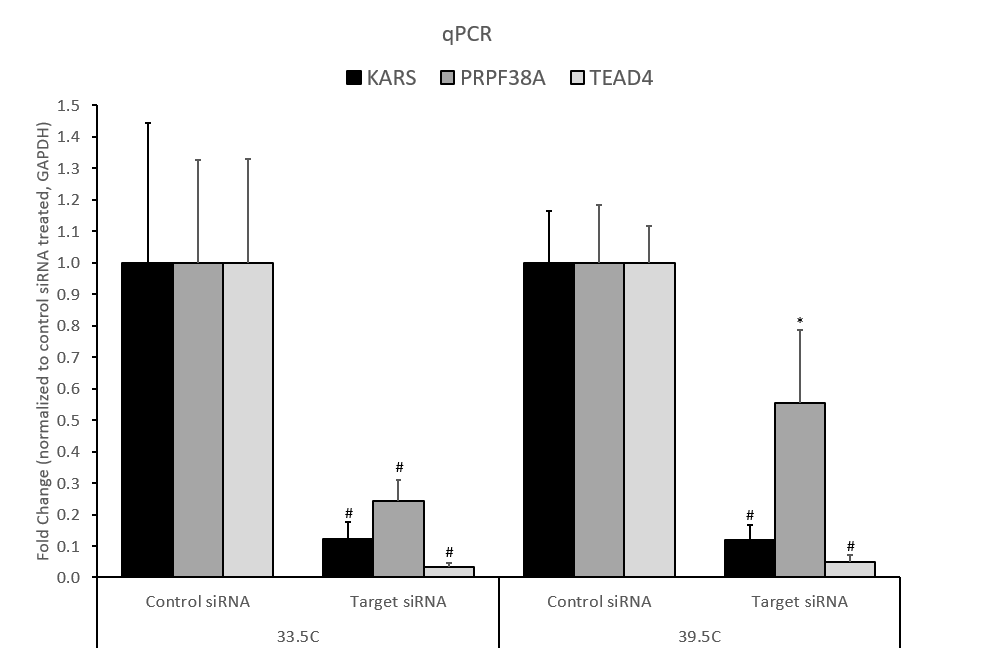
**

**
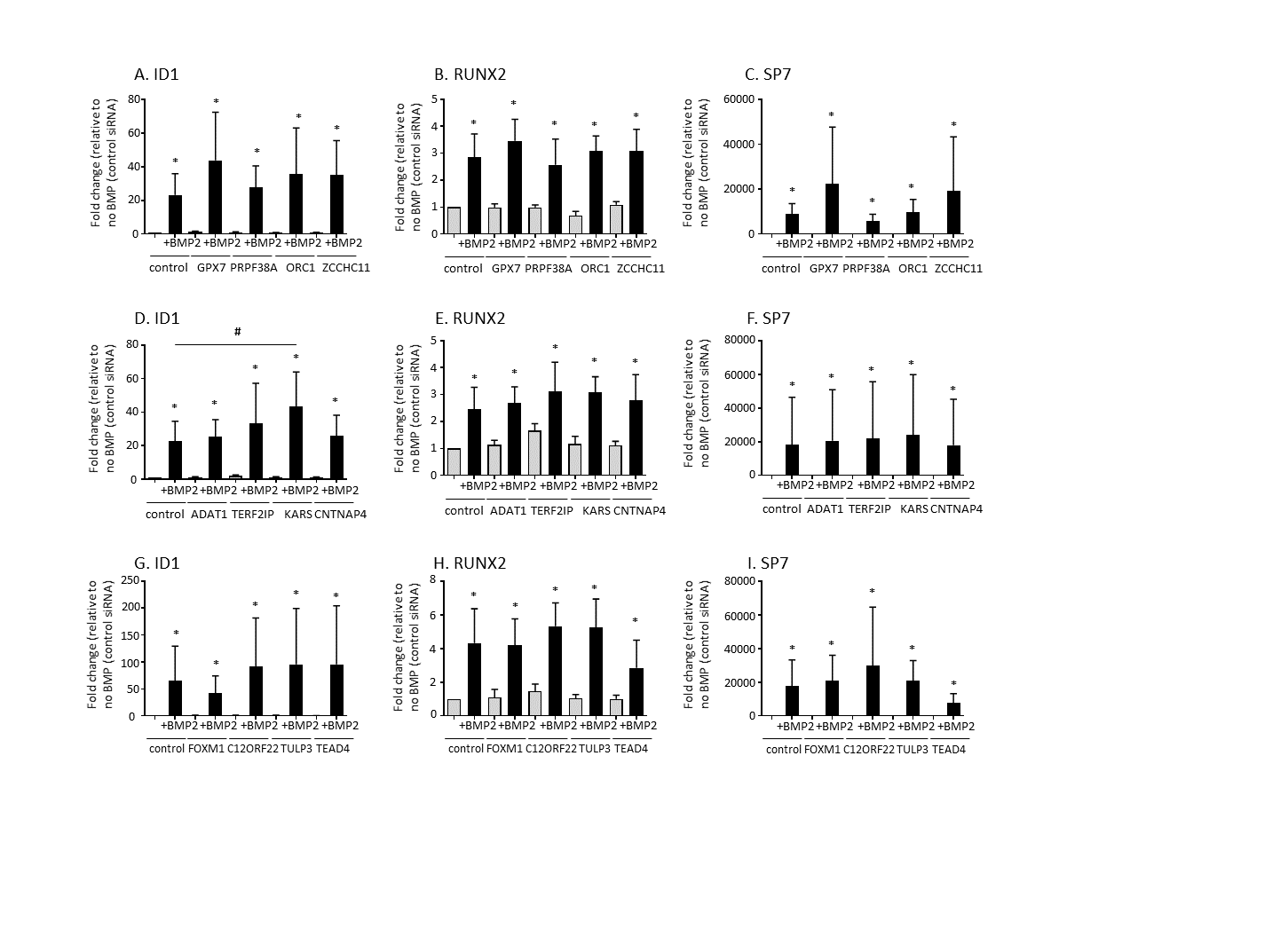
Figure S10. Gene expression of BMP signaling and osteoblast markers in hMSCs and BMP2-induced osteoblasts after siRNA knockdown of genes at the (A-C) *PRPF38A*, (D-F) *KARS*, and (G-I) *TEAD4* loci.** **p*<0.05 comparing no treatment to BMP2 treatment for each siRNA, #*p*<0.05 comparing control siRNA to siRNA for gene of interest.

**Figure S11. Gene expression of chondrogenic and later osteoblastic markers in hMSCs and BMP2-induced osteoblasts after siRNA knockdown of *PRPF38A*.** **p*<0.05 comparing no treatment to BMP2 treatment for each siRNA.

**
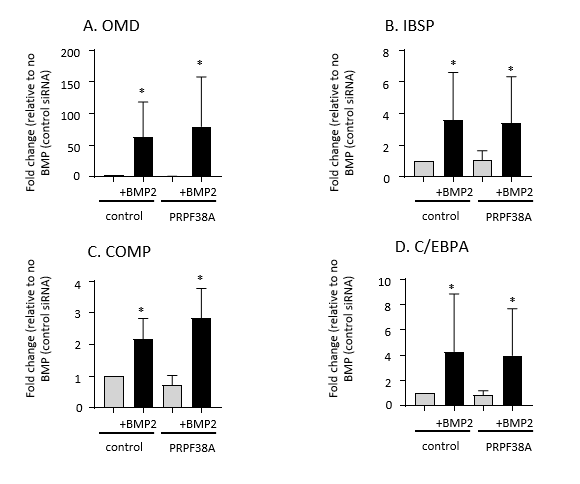
**

**Figure S12. CRISPR-Cas9 deletion at *PRPF38A* locus in hFOBs.** A. Lentiviral mCherry transduction efficiency. B. Sequencing results, showing the sizes, positions, and relative frequency of CRISPR deletions.

**
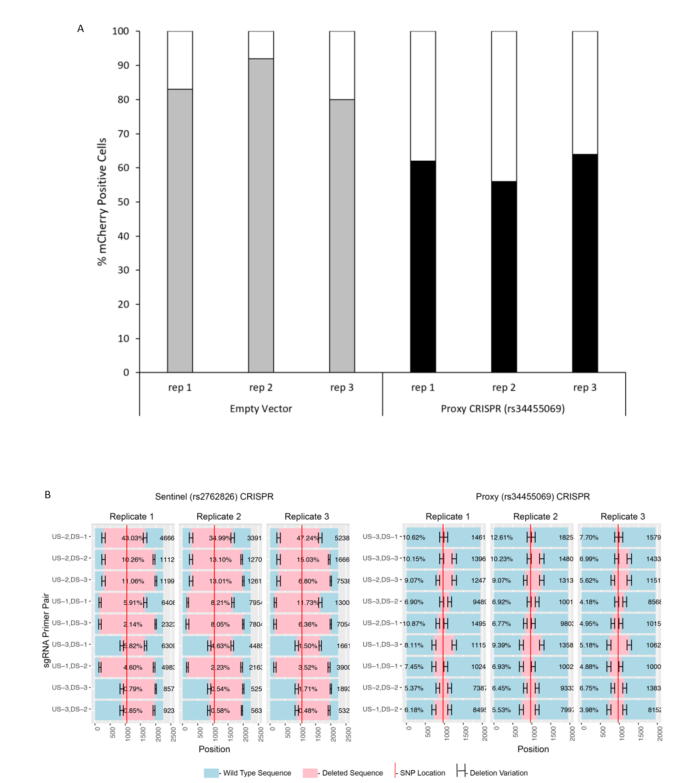
**

**Figure S13. rs34455069 is predicted to disrupt two transcription factor binding sites.** Position weight matrices for (A) KROX and (B) Sp1:Sp3 with rs34455069 boxed in red.

**
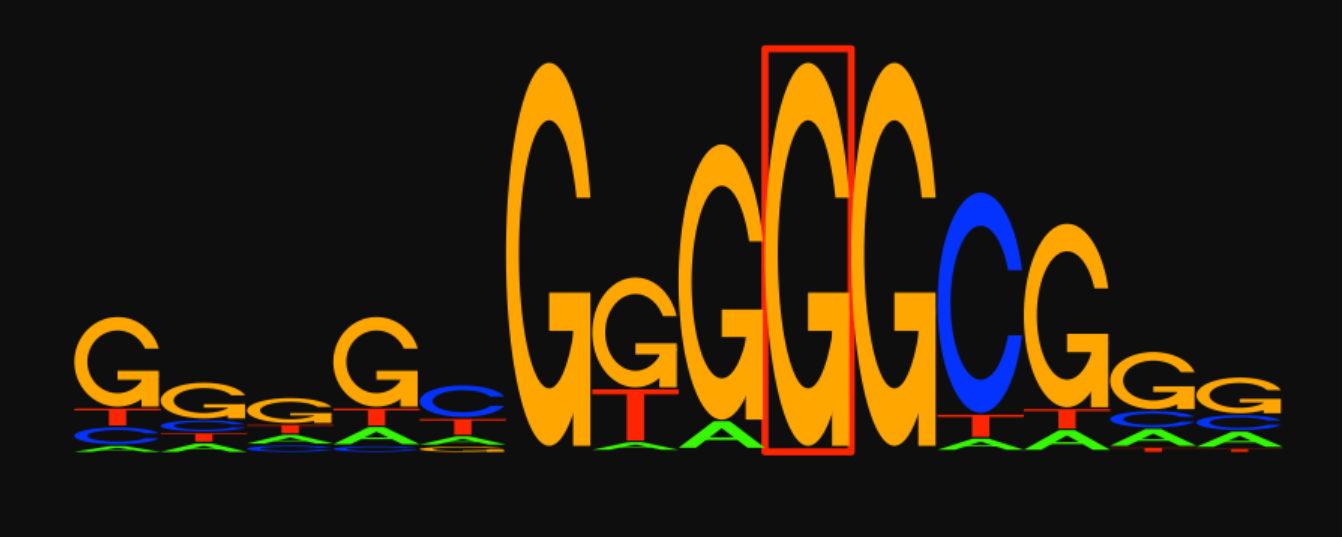
**

**
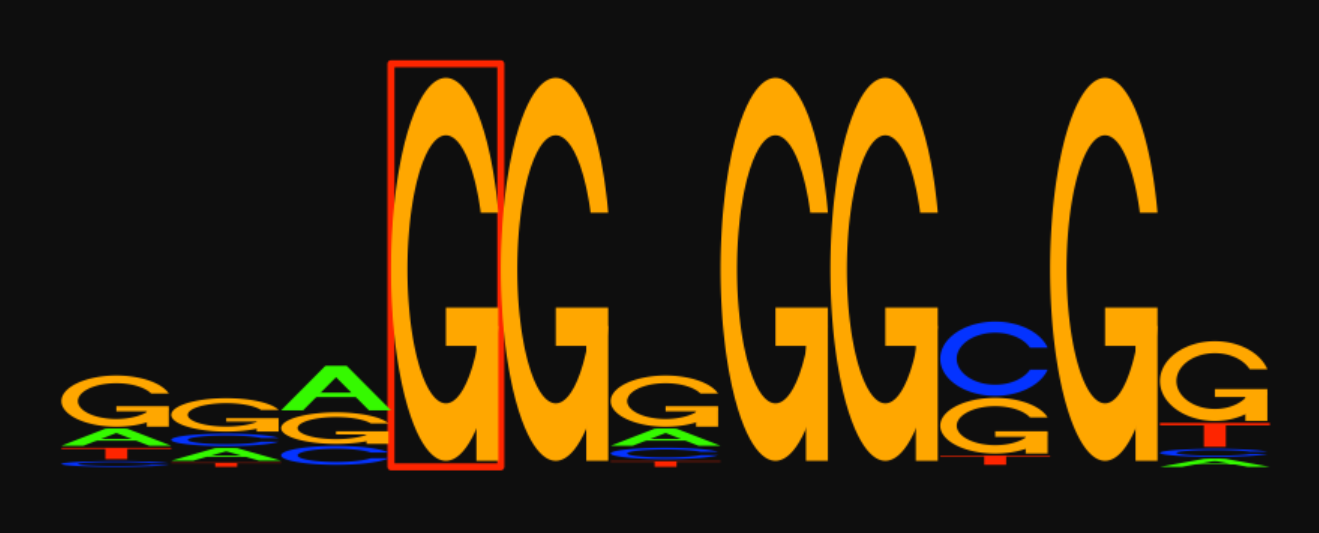
**

**Figure S14. ALSPAC mean SITAR curves for TBLH and skull BMC.**

**
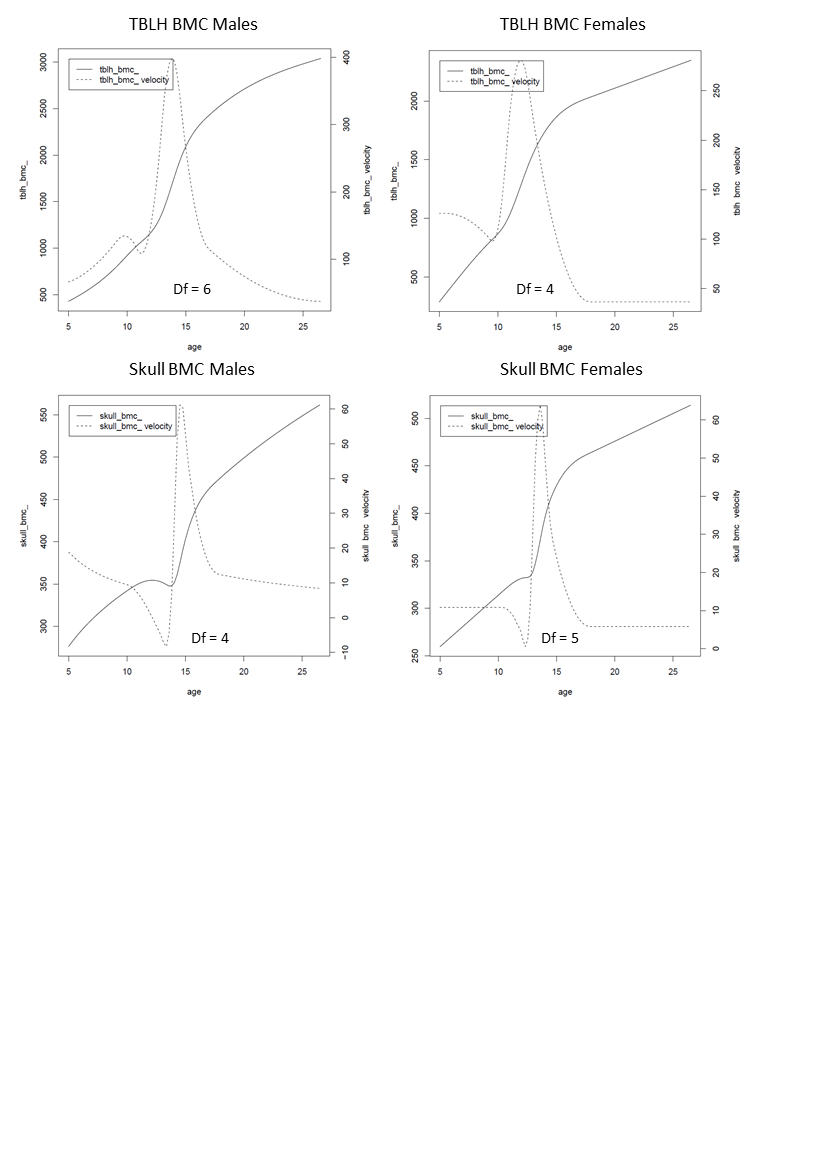
**
