# Supplementary material for: Genome-wide association study implicates novel loci and reveals candidate effector genes for longitudinal pediatric bone accrual
Source: Genome Biol. 2021 Jan 4;22:1. doi: 10.1186/s13059-020-02207-9 (PMC7780623; doi:10.1186/s13059-020-02207-9)
Supplement: Supplementary file 3 — Additional file 3. Review history. [file 13059_2020_2207_MOESM3_ESM.docx]

**Review History**

**First round of review**

**Reviewer 1**

Cousminer et al. have modelled longitudinal BMD (and BMC) trajectories in a pediatric population across distinct skeletal sites, followed by GWAS per model and skeletal site. The authors claim the identification of 40 loci of which 5 are novel. Replication in another pediatric cohort provided some evidence of replication for four of the novel loci and 1 of the known loci. Evidence for association with fracture risk later in life was sought on a recent UKBioBank publication (Morris et al. Nature Genetics) and an online resource (PheWeb), providing evidence for 5 and 8 loci, respectively. Functional follow-up was performed in-silico and on cell assays, implicating three novel genes in distinct loci.

I congratulate the authors for the large amount of work and effort placed on producing this publication. The idea is original and the longitudinal analysis scarce in the literature. Combining GWAS findings and functional work is outstanding and very much needed in the field of complex traits. The paper is well written and is a novel approximation to identify genes implicated in bone accrual during youth and osteoporosis susceptibility later in life.

Nevertheless, I have concerns which are presented below:

Main
1.     The work (and potentially the findings) of the authors challenge the current contention that the vast majority of genetic associations identified with bone mineral density along the life course correspond to bone accrual (peak BMD) associations. BMD variation is mostly explained (epidemiologically) by peak bone mass and much less by BMD change thereafter (bone loss representing a small fraction of the variation of BMD at a given timepoint). While it is true that longitudinal modeling has not been extensively applied (in contrast to cross-sectional analysis), one would expect that genetic variation influencing peak bone mass density would be seen associated also a later time points after peak bone mass acquisition, and this is not the case. Therefore, it is highly unexpected that so few of the associations are captures by extremely powered cross-sectional GWAS. If such associations are true, they are also very specific to an early life period and would require to have a very strong effect to be identified within the power setting of the current study (n=11,000) even if repeated measurements can increase the power.  The reported effect sizes are not consistent with this contention.
2.     Related to the previous point, the authors do not consider any correction for multiple testing, If I understand correctly the authors performed longitudinal GWAS for BMD and BMC; across three different models (size, timing and velocity) and six skeletal sites (without considering those done for sex and ethnic groups). Most of the reported associations are just surpassing the P<5E10-8 threshold and are unlikely to endure correction for multiple testing.
3.     The authors pursue replication in the ALSPAC cohort on TB-Less head BMD measurements. The analytical approach used in ALSPAC is unconventional and there should be some benefit of including ALSPAC investigators to run those analysis. It calls attention that the authors relaxed the replication framework to include proxy SNPs (r2>0.8). Here the authors should report also the replication of the sentinel SNPs. Justification about the required specificity assumption for associations across distinct skeletal sites and how including proxies can compensate for the lack of measurements is very much welcome. The authors could be more straight forward and state that at most 5 of the 40 loci replicated at a nominal significance level. This implies that the remaining 35 are still considered false positives until additional replication is provided.
4.     The fracture associations are also not fully convincing. Did the authors check the GEFOS any-type of fracture dataset (Trajanoska et al. BMJ)? Despite a lower sample size, fracture associations were better validated. It is interesting that for the PhebWEB browser exploration multiple test correction is considered but not for the other analyses. The P-value for association with fracture is P= 0.0890 (n=426824) which is in contrast with the 8.9E-05 described by the authors on page 10.  What was the definition of locus used along the paper?
5.     The authors could provide biological explanations on how they expect, that measurements with very high phenotypic and genetic correlation like that observed for BMD and ultrasound across distinct skeletal sites, hold such level of specificity. Associations are scattered across skeletal traits and models, more consistent with patchy random variation explaining the dispersion of the associations rather than biological underpinnings. Since the authors already estimated SNP-based heritability, it would be very informative to provide both the phenotypic and the genetic correlations between the longitudinal traits. The phenotypic and genetic correlations are solely described in the methods but not included in the results section.
6.     The authors "criticize" using physical proximity to annotate gene involvement across GWAS and use this as the rationale to move to TAD prioritization. While using TADs brings functionality into the equation, long range interactions are rather the exception and as shown before the closest gene  (luckily) has a high probability of being the underlying gene  (see Ref 1 below whose first author should be Morris J instead of 23andMe or see DOI: 10.1016/j.cels.2016.10.014 which are some examples in the bone field, followed by many more studies from other fields..
7.     The authors should discuss the bias underlying the expansion of candidate annotation (319 protein coding genes) to then weigh-in evidence on known implication in skeletal biology. Note that GWAS is hypothesis free and while identifying variants from genes implicated in bone biology can be used as a robust proof of concept, a large number of the associations arise from genes not previously known to be involved in bone biology. Therefore, this specific analysis is from my perspective weak.
8.     On the other hand, the "variant to gene mapping" is a more robust approach, as shown by the authors in their previous publication (Chesi et al., Nat Comm). Interestingly, the authors show some of the prioritized variants end up being previously identified variants from large cross-sectional GWAS.  Nevertheless, can the authors provide a biological explanation reconciling how the approach ends up linking a few GWAS signals of the longitudinal analysis in children with genes identified by the cross-sectional analysis? Can this not just be the result of chance?
9.     Can the authors provide a better description of the functional work? How are the genes to be taken forward for functional workup chosen? Is a flow diagram describing the selection procedures an idea?
10.     In the introduction the authors use the term aBMD to reference multiple papers, many of which do not use aBMD but rather "estimated" BMD from heel ultrasound measurements (eBMD). Also, throughout the paper statements are generalized for BMD measurements at distinct skeletal sites. Please be more accurate.
11.     The authors depict the longitudinal GWAS as a novel component of their approach. Nevertheless, work from the same group has been published earlier using a longitudinal approach and this should be straightforward described in the introduction see DOI: 10.1002/jbmr.3362 or for other growth traits, see DOI: 10.1126/sciadv.aaw3095

**Reviewer 2**

Cousimer, Wagley, Pippin and colleagues present an impressive analysis from GWAS to function for longitudinal pediatric bone accrual in 1885 children and adolescents.  This well-written manuscript reports trait heritability and performed GWAS analyses for 36 trait-parameter-site combinations and identified 27 loci at traditional significance thresholds, and 40 when including suggestive loci. Replication analyses in ALSPAC and associations with bone traits in adults support several of the associations. Multiple analyses are used to connect association signals to effector genes. At three loci, siRNA knockdown was used to test some candidate genes for function, and at one locus, genome editing showed that the SNP region affects PRPF38A expression.  These analyses identify candidate genes at the GWAS loci.

The large number of loci make a substantial contribution to the field of bone biology. Most of the loci show marginal evidence of association, not validated, and would not be considered significant if strictly accounting for the number of tests performed. While such a correction would be overly conservative, some may be false positive associations. Nonetheless, reporting these associations is valuable and may help identify genes for bone accrual.

The broadest interest to other genome biologists may be the variant-to-gene analyses. These are very challenging, and the approach presented here is a useful contribution.  The functional analyses provide support for some candidate genes, but are not definitive.

Major comments:

1.     The abstract describes identification of 40 loci, but the manuscript then reports that the definition of locus includes associations that do not meet traditional thresholds for genome-wide significance and does not consider potential false positives from performing 36 (correlated) genome-wide analyses. The abstract should include the p-value threshold used to define loci. The results should acknowledge the potential false positives from multiple tests, that 'support' may be from a highly correlated trait, and that variants in moderate pairwise LD may not reflect the same signal.
2.     The definition of 'novel loci' needs to be clarified. Are the 35 new compared to the >1000 listed in the introduction? What sources, traits, and life stages were used to define known loci (only the three papers named or other papers also)? A definition of novel should also be included in the text Table 1 footnote.
3.     The loci validated by ALSPAC should be indicated in Table 1, perhaps using an additional footnote. The text or table needs to clarify which loci could not be tested for validation and which were not validated.  Similarly, loci also associated with later-life fracture could be reported in Table 1.
4.     Did any loci include protein-coding variants? If so, in what genes?
5.     The GWAS-eQTL analysis should consider colocalization, as many results reported may represent different signals. At least look up the sentinel eQTL variant for each identified gene and report when LD between the sentinel GWAS variant and sentinel eQTL variant exceeds a threshold consistent with the variants representing the same signal.  
6.     The choice of a liberal r2>=.5 value for accessible SNP to gene interactions should be justified.  This liberal choice seems likely to lead to false positive interactions.  It appears that European LD is used even though many individuals are African American.  Could fine-mapping be performed across ancestries?  This choice affects the gene implicated at signal 17.
7.     Several variant-to-gene-to-function analyses are performed (literature function, location in TAD, promoter interaction, eQTL, functional study) and some apparently show discrepant results. Do any of the 22 genes from 6 loci from promoter interaction overlap with the 28 genes from the eQTL analysis? Which variant-to-function analyses identified the genes that show functional effects in Figures 3-7? In addition, which assays in Figures 3-7 showed consistent results for a gene and which are inconsistent?  Further interpretation of the inconsistent results would be useful.
8.     The genes tested using knockdown were selected by variant-to-gene mapping and eQTL, however, only four genes were tested per locus.  It's striking that the variant implicated in PRPF38A function is located at the promoter of CC2D1B, but CC2D1B was not tested. Could CC2D1B be a better candidate gene?  If this gene was considered and excluded from testing, the reasons could strengthen evidence for PRPF38A.  The implications of testing only loci based on chromatin interactions and eQTL should be discussed.
9.     Figure 3 should better show the genes and variants proposed to have functional effects. In Figure 3 BCD GWAS plots, indicate the variant involved in the interaction with promoters as shown in EFG. What is the evidence of association for these variants? This question is especially relevant at signal 17, where Figure 3F implicates rs8051713, which appears to have association evidence of only P=1E-3 in Figure 3C, not even marginally significant.  In Figure 3 EFG, all proxy variants should be shown, and genes tested for function should be indicated (highlighted?).

Minor comments:
1.     The numbers of the individuals in ancestry- and sex-specific analyses should be included in the main text. The 11,000 number of scans doesn't reflect the sampling variability of the study.  
2.     BMC should be defined at first use
3.     The abstract results could replace the vague 'some' with the number of loci also associated with later-life fracture risk
4.     Table 1 footnote, what is the LD r2 value for the two signals that 'are not in LD'? Below what threshold?
5.     Should signal S13 on Table 1 include footnote d?
6.     Table 1 could be condensed to report results for a single trait per locus, with a column indicating other traits that were also identified. Full results could still be reported in the supplement.
7.     The text reports African American and non-African American, while the figures report black and non-black. Which term was used when ethnicity was collected?
8.     The text includes lengthy descriptions of genes reported previously in the literature, which are also shown in Table 2.  The text could be condensed or the table could be moved to the supplement.
9.     Page 12, line 30, what is an 'open proxy SNP'?
10.     Page 14, lines 50-53, sentence repeats after knockdown of PRPF38A and KARS.
11.     Page 17, lines 18-19 reword 'provided implicated' leads?
12.     The Figure 3 legend should explain the overview cartoons in part A
13.     Figure 4 line breaks in x- and y-axes make the figure hard to read

**Authors’ response to reviewers**

Reviewer reports:

Reviewer #1: Cousminer et al. have modelled longitudinal BMD (and BMC) trajectories in a pediatric population across distinct skeletal sites, followed by GWAS per model and skeletal site. The authors claim the identification of 40 loci of which 5 are novel. Replication in another pediatric cohort provided some evidence of replication for four of the novel loci and 1 of the known loci. Evidence for association with fracture risk later in life was sought on a recent UKBioBank publication (Morris et al. Nature Genetics) and an online resource (PheWeb), providing evidence for 5 and 8 loci, respectively. Functional follow-up was performed in-silico and on cell assays, implicating three novel genes in distinct loci.

I congratulate the authors for the large amount of work and effort placed on producing this publication. The idea is original and the longitudinal analysis scarce in the literature. Combining GWAS findings and functional work is outstanding and very much needed in the field of complex traits. The paper is well written and is a novel approximation to identify genes implicated in bone accrual during youth and osteoporosis susceptibility later in life.

*Response: We thank the reviewer for the positive assessment of our work. However, we must correct one point above, namely that of the 40 loci identified, the majority were actually novel and just 5 were known.*

Nevertheless, I have concerns which are presented below:

Main
1. The work (and potentially the findings) of the authors challenge the current contention that the vast majority of genetic associations identified with bone mineral density along the life course correspond to bone accrual (peak BMD) associations. BMD variation is mostly explained (epidemiologically) by peak bone mass and much less by BMD change thereafter (bone loss representing a small fraction of the variation of BMD at a given timepoint). While it is true that longitudinal modeling has not been extensively applied (in contrast to cross-sectional analysis), one would expect that genetic variation influencing peak bone mass density would be seen associated also a later time points after peak bone mass acquisition, and this is not the case. Therefore, it is highly unexpected that so few of the associations are captures by extremely powered cross-sectional GWAS. If such associations are true, they are also very specific to an early life period and would require to have a
very strong effect to be identified within the power setting of the current study (n=11,000) even if repeated measurements can increase the power. The reported effect sizes are not consistent with this contention.

*Response: The effect sizes presented here reflect SITAR random effects and cannot be easily translated back to g/cm2. For example, a positive beta for the a-size parameter means that the per-allele effect of that SNP increases the a-size SITAR parameter relative to the population mean. Therefore it is difficult to make any inferences about how these effects compare to peak bone accrual or adult effect sizes.*

2. Related to the previous point, the authors do not consider any correction for multiple testing, If I understand correctly the authors performed longitudinal GWAS for BMD and BMC; across three different models (size, timing and velocity) and six skeletal sites (without considering those done for sex and ethnic groups). Most of the reported associations are just surpassing the P<5E10-8 threshold and are unlikely to endure correction for multiple testing.


*Response: We apologize that this point was unclear in the manuscript. In fact, we used PhenoSpD (https://pubmed.ncbi.nlm.nih.gov/30165448/) to determine the number of independent tests, given the high degree of correlation between our phenotypes. PhenoSpD determined that there were 16 independent phenotypes, and we discuss in the paper that one locus would survive this stringent multiple test correction. However, given the difficulty in performing adequate replication analyses due to the scarcity of cohorts with dense DXA measurements across childhood, we instead opted to include loci with a more relaxed P-value threshold and provide orthogonal evidence in support of the observed loci as true positives. We believe this balance is correct, given we lay out the level of support at each locus, and this largely aligns with Reviewer 2’s point that “While such a correction would be overly conservative, some may be false positive associations. Nonetheless, reporting these associations is valuable and may help identify genes for bone accrual”

However, in response to Reviewer 1’s point, we have tempered the language in the Abstract as follows:

“We identified 40 loci (35 novel) with various degrees of supportive evidence (associated at P < 5x10-8 or P < 1x10-6 with more than one skeletal site/phenotype), half residing in topological associated domains harboring known bone genes.”

Additionally, we edited the Results as follows on p.8:

“Acknowledging the large number of statistical tests performed, we used several strategies to prioritize loci for further analyses. First, given the high correlation between aBMD and BMC and among different skeletal sites (Supplementary Figure 3; Supplementary Tables 6 and 7), we used PhenoSpD19,20 to calculate the number of independent tests. This revealed an equivalent of 16 independent tests, resulting in a corrected significance threshold of P < 3.1x10-9, which yielded one locus (signal 26, rs201392388, nearest gene FGF16) surpassing the corrected genome-wide significance threshold accounting for multiple testing.”

Finally, in the Discussion, we have added that additional replication by other studies is warranted.*

3. The authors pursue replication in the ALSPAC cohort on TB-Less head BMD measurements. The analytical approach used in ALSPAC is unconventional and there should be some benefit of including ALSPAC investigators to run those analysis. It calls attention that the authors relaxed the replication framework to include proxy SNPs (r2>0.8). Here the authors should report also the replication of the sentinel SNPs. Justification about the required specificity assumption for associations across distinct skeletal sites and how including proxies can compensate for the lack of measurements is very much welcome. The authors could be more straightforward and state that at most 5 of the 40 loci replicated at a nominal significance level. This implies that the remaining 35 are still considered false positives until additional replication is provided.

*Response: Association values for the sentinel SNPs were presented in Supplementary Table 9, highlighted in bold. We have also edited our presentation of the replication results to be more straightforward:*

“Thus, five of our loci replicated at a nominal significance level (Supplementary Table 9), one of which also showed suggestive support in the heel eBMD lookup (signal S11, rs2564086, nearest gene SOX11).”

4. The fracture associations are also not fully convincing. Did the authors check the GEFOS any-type of fracture dataset (Trajanoska et al. BMJ)? Despite a lower sample size, fracture associations were better validated. It is interesting that for the PhebWEB browser exploration multiple test correction is considered but not for the other analyses. The P-value for association with fracture is P= 0.0890 (n=426824) which is in contrast with the 8.9E-05 described by the authors on page 10. What was the definition of locus used along the paper?

*Response: We have performed a candidate lookup in the Trajanoska et al dataset, and noted that the three lookup approaches (UKBB GWAS for any fracture (Morris et al), PheWEB for specific fracture types, and Trajanoska et al) converged on support for signal S13, previously significantly associated with heel eBMD and here suggestively associated with total hip BMC a-size. We have revised the Abstract and Results to reflect the consistent fracture results for this locus. However, we also note that there is typically very little agreement between loci associated with BMD and fracture, as fracture is a measure of bone quality while BMD measures bone strength.*


5. The authors could provide biological explanations on how they expect, that measurements with very high phenotypic and genetic correlation like that observed for BMD and ultrasound across distinct skeletal sites, hold such level of specificity. Associations are scattered across skeletal traits and models, more consistent with patchy random variation explaining the dispersion of the associations rather than biological underpinnings. Since the authors already estimated SNP-based heritability, it would be very informative to provide both the phenotypic and the genetic correlations between the longitudinal traits. The phenotypic and genetic correlations are solely described in the methods but not included in the results section.

*Response: We originally omitted an in-depth description of the phenotypic and genetic correlation results due to word count limitations, but have added this into the Results in a new section entitled “Phenotypic and genetic correlation among skeletal sites”.*

*While these correlations suggest that some loci should act on bone density more broadly across the skeleton, particularly with sharing between TBLH, femoral neck, and total hip, our correlation results show that some sites are likely to be quite different (e.g. the skull and radius). Indeed, our genetic results are consistent with these findings. To clarify that our genetic association results are in line with expectations based on the correlation results, we have added a new Supplementary Figure 4, showing that the majority of loci were associated with total hip, femoral neck, and TBLH, with 10 loci associated with more than one of these three phenotypes. Of the 5 radius loci, only one shared an association with femoral neck, and the skull association was completely distinct. Additionally, some loci in previous work have been shown to have differing or even discordant effects at different skeletal sites (e.g. in PMID: 29240982), particularly at the radius. Furthermore, the majority of loci associated with only one SITAR parameter, in line with the clustering of a-size, b-timing, and c-velocity phenotypes mostly with themselves in Supplementary Figure 3C.*

*These observations likely reflect that the TBLH measure encompasses the total hip, femoral neck, and spine measures, and that total hip also includes the femoral neck. The hip and spine are weight-bearing, composed of a mix of cortical and trabecular bone. In contrast, the radius is not weight-bearing and is made up of cortical bone, whereas the skull also has a distinct bone structure and nearly 80% of skull size is attained by age 5, being more or less stable until puberty.*

*The following text was added to p.10:*

*“In line with expectations based on the phenotypic and genetic correlation results, we observed the highest number of associated loci for the total hip, femoral neck, and TBLH (Supplementary Figure 4A), followed by the spine. Additionally, the majority of shared associations were observed across more than one of these three traits. Also in line with expectations based on the clustering of genetic correlation results within a-size, b-timing, and c-velocity for different skeletal sites, the majority of loci were associated with only one of these longitudinal parameters (Supplementary Figure 4B).”

We also added the following to the Discussion on p. 19:

“We noted differences in heritability among skeletal sites, and both phenotypic and genetic correlation estimates were in line with expectations based on bone composition, degree of load bearing, and timing of development. For instance, the total hip and femoral neck, followed by the spine, were the most correlated with each other and resulted in the most shared genetic associations, together with the TBLH. Indeed, these three skeletal sites are weight-bearing and are made up of a mix of trabecular and cortical bone (Clarke, 2008). Additionally, they collectively make up a large proportion of the TBLH measure, and the total hip includes the femoral neck. In contrast, the 1/3 distal radius is less load-bearing and is composed nearly entirely of cortical bone, and the skull is distinct both in its bone structure and timing of growth; the majority of skull growth is completed by age 5 and is relatively stable until puberty (Hahn et al., 1984).“*

6. The authors "criticize" using physical proximity to annotate gene involvement across GWAS and use this as the rationale to move to TAD prioritization. While using TADs brings functionality into the equation, long range interactions are rather the exception and as shown before the closest gene (luckily) has a high probability of being the underlying gene (see Ref 1 below whose first author should be Morris J instead of 23andMe or see DOI: 10.1016/j.cels.2016.10.014 which are some examples in the bone field, followed by many more studies from other fields.

*Response: Ref 1 has been corrected.

There are examples in the literature of GWAS loci being resolved to both the nearest and more distal genes. For example, the GTEx Consortium has shown that often a more distal is the implicated causal gene (PMID: 29022597 – the primary GTEx paper in Nature: “only 40% of GWAS signals co-localize with their nearest expressed gene, a finding that has important implications for the functional characterization of GWAS results”). Indeed, the TAD-based approach has also been shown to identify disease-relevant genes and pathways more often than simply taking the nearest gene (e.g. PMID: 28792001 and https://www.biorxiv.org/content/10.1101/2019.12.20.885426v1). Thus, we opted to take a broader view and include all genes within the chromatin interaction unit of the TAD so as not to miss any key candidate genes.*

7. The authors should discuss the bias underlying the expansion of candidate annotation (319 protein coding genes) to then weigh-in evidence on known implication in skeletal biology. Note that GWAS is hypothesis free and while identifying variants from genes implicated in bone biology can be used as a robust proof of concept, a large number of the associations arise from genes not previously known to be involved in bone biology. Therefore, this specific analysis is from my perspective weak.

*Response: Despite not having been seen before in a GWAS setting, many genes near our signals have evidence for involvement in bone biology from mouse studies, GO annotations and pathways, and monogenic diseases. We do, however, acknowledge that despite these genes having known biology related to osteoblast or osteoblast development or skeletal development, many of the association signals have not been reported from GWAS efforts before. We believe this is due to (1) the inclusion of 6 distinct skeletal sites, more than typically assessed in BMD GWAS previously, and (2) the use of the longitudinal SITAR modelling, which has not previously been applied to bone accrual in GWAS efforts.

We recognize that we are highlighting known biology on many of these loci, but we believe this aids the paper in two ways. First, we are openly guiding the reader on what is known in the vicinity of the locus, as defined by TAD boundaries. Second, by identifying the loci without any indication of known biology, we set a high bar for ourselves by pursuing those functionally, which turned out to be relatively fruitful in revealing new biological insights. We have added wording to this Discussion to reflect this point.*


8. On the other hand, the "variant to gene mapping" is a more robust approach, as shown by the authors in their previous publication (Chesi et al., Nat Comm). Interestingly, the authors show some of the prioritized variants end up being previously identified variants from large cross-sectional GWAS. Nevertheless, can the authors provide a biological explanation reconciling how the approach ends up linking a few GWAS signals of the longitudinal analysis in children with genes identified by the cross-sectional analysis? Can this not just be the result of chance?

*Response: We thank the reviewer in acknowledging the robustness of our variant-to-gene mapping approach. Although our approach is somewhat different from what has gone before in the BMD genetics field, we would have been surprised if there had been absolutely no overlap with previous signals; indeed these true positives lend support to our overall approach and results. Our studies with the Early Growth Genetics (EGG) consortium revealed a blend of shared and distinct loci when treating pediatric BMI either dichotomously (childhood obesity) or quantitatively – so clearly trait definitions influence the loci that are yielded from such efforts. Furthermore, there was degree of overlap between pediatric BMI loci and adult BMI loci. The exact biological reasons behind such observations warrants further investigation, but there is growing evidence that many loci operate very early in life, while a few others confer their effects more apparently a little later in life due to processes that still require full elucidation (see, for example, PMID 31840077 and 31575865).*

9. Can the authors provide a better description of the functional work? How are the genes to be taken forward for functional workup chosen? Is a flow diagram describing the selection procedures an idea?

*Response: Given our focus on implicating novel genes at our loci, we first limited the number of loci for consideration to the six which had variant-to-gene promoter loops present in our hMSC osteoblast dataset as well as pan-tissue eQTL support in GTEx. Three of these loci pointed only to genes with no prior evidence of osteogenic effects, so we opted to perform siRNA knockdowns at these three loci specifically.

To clarify, we have added to p.13:
“About half of our prioritized loci lacked clear functional candidate effector genes, with three signals showing evidence of target gene promoter interactions in our hMSC-derived osteoblasts.”

Additionally, we have added a flow chart as new Supplementary Figure 5.*

10. In the introduction the authors use the term aBMD to reference multiple papers, many of which do not use aBMD but rather "estimated" BMD from heel ultrasound measurements (eBMD). Also, throughout the paper statements are generalized for BMD measurements at distinct skeletal sites. Please be more accurate.

*Response: We have gone through the paper and checked each instance of “aBMD” for accuracy.*

11. The authors depict the longitudinal GWAS as a novel component of their approach. Nevertheless, work from the same group has been published earlier using a longitudinal approach and this should be straightforward described in the introduction see DOI: 10.1002/jbmr.3362 or for other growth traits, see DOI: 10.1126/sciadv.aaw3095

*Response: We have edited the text as follows:

Abstract:

“genetic discovery has been primarily limited to cross-sectional study designs”
“Using a new longitudinal approach”

Discussion, p.17:

“To complement cross-sectional genetic studies of bone trait measurements and our previous work implementing a linear mixed model to integrate longitudinal measures (Mitchell 2015)”*


Reviewer #2: Cousimer, Wagley, Pippin and colleagues present an impressive analysis from GWAS to function for longitudinal pediatric bone accrual in 1885 children and adolescents. This well-written manuscript reports trait heritability and performed GWAS analyses for 36 trait-parameter-site combinations and identified 27 loci at traditional significance thresholds, and 40 when including suggestive loci. Replication analyses in ALSPAC and associations with bone traits in adults support several of the associations. Multiple analyses are used to connect association signals to effector genes. At three loci, siRNA knockdown was used to test some candidate genes for function, and at one locus, genome editing showed that the SNP region affects PRPF38A expression. These analyses identify candidate genes at the GWAS loci.

The large number of loci make a substantial contribution to the field of bone biology. Most of the loci show marginal evidence of association, not validated, and would not be considered significant if strictly accounting for the number of tests performed. While such a correction would be overly conservative, some may be false positive associations. Nonetheless, reporting these associations is valuable and may help identify genes for bone accrual.

The broadest interest to other genome biologists may be the variant-to-gene analyses. These are very challenging, and the approach presented here is a useful contribution. The functional analyses provide support for some candidate genes, but are not definitive.

*Response: We thank the reviewer for an overall positive assessment of our work.*


Major comments:

1. The abstract describes identification of 40 loci, but the manuscript then reports that the definition of locus includes associations that do not meet traditional thresholds for genome-wide significance and does not consider potential false positives from performing 36 (correlated) genome-wide analyses. The abstract should include the p-value threshold used to define loci. The results should acknowledge the potential false positives from multiple tests, that 'support' may be from a highly correlated trait, and that variants in moderate pairwise LD may not reflect the same signal.

*Response: We thank the reviewer for this key point, and agree that the reader needs further guidance in this regard. We have added the P-value threshold to the abstract. We have also added the following to the Discussion to reflect the points raised by the reviewer: “Still, there is potential for false positives among our results, and there is a possibility that variants in moderate pairwise LD do not reflect the same underlying signal. Therefore, additional replication by other studies is required.”*

2. The definition of 'novel loci' needs to be clarified. Are the 35 new compared to the >1000 listed in the introduction? What sources, traits, and life stages were used to define known loci (only the three papers named or other papers also)? A definition of novel should also be included in the text Table 1 footnote.

*Response: Here, “novel locus” means “not previously reported in a GWAS study of BMD”—either in children or in adults. To address this point, we changed “novel” in the abstract to read “not previously reported in GWAS of bone density.” We also added this to the footnote of Table 1.*

3. The loci validated by ALSPAC should be indicated in Table 1, perhaps using an additional footnote. The text or table needs to clarify which loci could not be tested for validation and which were not validated. Similarly, loci also associated with later-life fracture could be reported in Table 1.

*Response: The ALSPAC data have been added to Table 1. However, we have opted to highlight two loci with support for involvement in fracture risk from multiple lookup approaches rather than the total number with more tenuous support.*

4. Did any loci include protein-coding variants? If so, in what genes?

*Response: Three genes contained nonsynonymous protein-coding variants in high LD with our sentinel signals (r2>0.8)--CC2D1B, KTI12, and ZFYVE9, all at signal 1. The following has been added the Results section, p.15:

“Although additional candidate genes are present at the signal 1 locus, including three with nonsynonymous variants in LD (r2>0.8) with the sentinel SNP (the nearest gene CC2D1B, ZFYVE9, and KTI12), we opted to target genes with both Capture C and eQTL evidence.”*

5. The GWAS-eQTL analysis should consider colocalization, as many results reported may represent different signals. At least look up the sentinel eQTL variant for each identified gene and report when LD between the sentinel GWAS variant and sentinel eQTL variant exceeds a threshold consistent with the variants representing the same signal.

*Response: Since there is no bone data in GTEx, we opted to view the eQTL results as simply one line of supportive evidence, among many, for the target genes. It is not clear in which tissue context we would perform coloc analysis.*

6. The choice of a liberal r2>=.5 value for accessible SNP to gene interactions should be justified. This liberal choice seems likely to lead to false positive interactions. It appears that European LD is used even though many individuals are African American. Could fine-mapping be performed across ancestries? This choice affects the gene implicated at signal 17.

*Response: We chose to begin with a modest r2 value to “rule in” rather than “rule out” variants i.e. to comprehensively appraise SNPs in the region. These are subsequently filtered by insisting on each fulfilling the two strict criteria of (1) residing in open chromatin AND (2) being in physical contact with a gene promoter(s). These are up to the reader to interpret the findings of our approach and may include some false positives, but we have confidence in the presented results given the key additional constraints applied. In regards to fine-mapping, the African American subset of samples was rather small (25% of the overall cohort), so we lack the power to do detailed fine-mapping. Finally, we have added LocusZoom plots with African American LD to new Supplementary Figure 7C.*

7. Several variant-to-gene-to-function analyses are performed (literature function, location in TAD, promoter interaction, eQTL, functional study) and some apparently show discrepant results. Do any of the 22 genes from 6 loci from promoter interaction overlap with the 28 genes from the eQTL analysis? Which variant-to-function analyses identified the genes that show functional effects in Figures 3-7? In addition, which assays in Figures 3-7 showed consistent results for a gene and which are inconsistent? Further interpretation of the inconsistent results would be useful.

*Response: Five genes overlap between the pan-tissue eQTL and variant-to-gene mapping approaches (ADAT1, GPX7, ORC1, PRPF38A, and ZCCHC11). Notably, the gene for which we present additional follow-up experiments and highlight in the paper, PRPF38A, is among these. Please also see the response to Reviewer 1, question 9. In general, we used a “rule in” philosophy, i.e. we’re not ruling out other genes at a given locus, rather we have confidence in the leads we see in our particular setting, to settle on this gene as being supported by all approaches for in-depth analysis in this paper.*

8. The genes tested using knockdown were selected by variant-to-gene mapping and eQTL, however, only four genes were tested per locus. It's striking that the variant implicated in PRPF38A function is located at the promoter of CC2D1B, but CC2D1B was not tested. Could CC2D1B be a better candidate gene? If this gene was considered and excluded from testing, the reasons could strengthen evidence for PRPF38A. The implications of testing only loci based on chromatin interactions and eQTL should be discussed.

*Response: We did not test CC2D1B for several reasons. First, our associated variants did not contact this gene in hMSC-derived osteoblasts. Furthermore, there is no literature supporting CC2D1B as a plausible candidate in bone, although we are not ruling out that it could also have an impact on osteogenesis. Here, we simply present our findings that PRPF38A DOES appear to have an effect on bone biology. Additionally, previous work has shown that promoter-promoter contacts are prevalent, and that promoters can act as enhancers for other genes (PMID: 31501517 and PMID: 28581502).

We have added the following text to the discussion:

“Additionally, we have tested selected candidate genes to “rule in” their effects on bone biology; this does not exclude other potential target genes as functionally relevant.”*


9. Figure 3 should better show the genes and variants proposed to have functional effects. In Figure 3 BCD GWAS plots, indicate the variant involved in the interaction with promoters as shown in EFG. What is the evidence of association for these variants? This question is especially relevant at signal 17, where Figure 3F implicates rs8051713, which appears to have association evidence of only P=1E-3 in Figure 3C, not even marginally significant. In Figure 3 EFG, all proxy variants should be shown, and genes tested for function should be indicated (highlighted?).

*Response: This figure has been redrawn to improve clarity and reflect the Reviewer’s suggestions.*

Minor comments:
1. The numbers of the individuals in ancestry- and sex-specific analyses should be included in the main text. The 11,000 number of scans doesn't reflect the sampling variability of the study.

*Response: The sample numbers have been added in the main text.*

2. BMC should be defined at first use

*Response: This change has been made*

3. The abstract results could replace the vague 'some' with the number of loci also associated with later-life fracture risk

*Response: Changes to the abstract have been made to highlight the convergent evidence for signal S13 rather than the total number of loci with suggestive evidence.*

4. Table 1 footnote, what is the LD r2 value for the two signals that 'are not in LD'? Below what threshold?

*Response: The r2 between the two variants at signals 22 and 23 is 0.05. This has been added to the table footnote.*

5. Should signal S13 on Table 1 include footnote d?

*Response: We thank the reviewer for their careful attention to detail and have made this correction.*


6. Table 1 could be condensed to report results for a single trait per locus, with a column indicating other traits that were also identified. Full results could still be reported in the supplement.

*Response: We believe it is best to show the complete data in the main table, but will make this adjustment if the Editors believe it would be beneficial.*

7. The text reports African American and non-African American, while the figures report black and non-black. Which term was used when ethnicity was collected?

*Response: The question asked on the demographic questionnaire was “Race: Black or African American; Yes, No, Unknown.” We have updated the text and figures to reflect the wording used on the questionnaire.*

8. The text includes lengthy descriptions of genes reported previously in the literature, which are also shown in Table 2. The text could be condensed or the table could be moved to the supplement.

*Response: We believe the table is useful as a quick overview of the candidate genes found, with more in-depth description found in the text. We will make this change if the Editors believe it would be beneficial.*

9. Page 12, line 30, what is an 'open proxy SNP'?

*Response: To clarify, we have changed the wording to: “accessible proxy SNP (a SNP falling in an open ATAC-seq peak in high LD with the sentinel variant)”*

10. Page 14, lines 50-53, sentence repeats after knockdown of PRPF38A and KARS.

*Response: This sentence was revised for clarity and the repetition removed.*

11. Page 17, lines 18-19 reword 'provided implicated' leads?

*Response: This sentence was revised for clarity.*

12. The Figure 3 legend should explain the overview cartoons in part A

*Response: We have added a description of this panel to the figure legend as follows:*

*“We first identify all SNPs in high LD with our sentinel associated variant. These are then filtered by residing in open chromatin as assessed by ATAC-seq in hMSC-derived osteoblasts. The open chromatin variants are subsequently filtered by being in direct physical contact with gene promoter baits. Finally, siRNA knockdown experiments are performed for a subset of these contacted genes to assess the impact on osteogenesis.”*

13. Figure 4 line breaks in x- and y-axes make the figure hard to read

*Response: We have redrawn this figure for clarity.*

**Second round of review**

**Reviewer 1**

I commend the authors for the exhaustive job addressing reviewers' comments and apologise wholeheartedly for the belated submission of my review (as I lost track of the repeated journal's requests in my spam folder).

The revised version addresses my comments. Only, the authors state in the rebuttal: "However, we also note that there is typically very little agreement between loci associated with BMD and fracture, as fracture is a measure of bone quality while BMD measures bone strength."

This is completely inaccurate as ALL fracture loci discovered to date are in fact, BMD loci. Bone quality is more an umbrella term to describe a set of characteristics of bone tissue that, in addition to density, BOTH contribute to bone strength. This contention was not taken forward in the manuscript (only in the reply to the reviewer) so is not a problem.

**Reviewer 2**

The authors have addressed many comments and improved the manuscript.  However, concerns remain.

1.     (Major) I am not satisfied by the response to major comment 5.  The authors respond that it is not clear which tissue to use to further characterize the eQTL results, but Supplementary Table 17 clearly lists tissues corresponding to each result. As presented, the results indicate only that these genes are expressed in a tissue evaluated by GTEx.  The analyses are insufficient to call these results (page 14, line 46) “evidence linking our loci to effector genes” and then to subsequently name genes “with eQTL evidence” on page 15.  At least for the genes reported as having eQTL evidence, ensure that the sentinel GWAS variant (or it’s closest proxy; weaker proxies do not provide supporting evidence) could feasibly be responsible for that gene’s eQTL association in at least one tissue.  

Alternatively, the eQTL results could be removed from the manuscript.

2.     (Minor) In response to major comment 9, edits to Figure 3 removed several figure panels corresponding to two loci (now in Supplementary Figure 7), but the legend to Figure 3 still describes three loci with parts A through G and does not describe the forest plots or SITAR curves by genotype.

**Authors’ response to reviewers**

We thank the reviewers for the overall positive critique of our manuscript. Please find point-by-point responses below.

Reviewer #2: The authors have addressed many comments and improved the manuscript. However, concerns remain.

1. (Major) I am not satisfied by the response to major comment 5. The authors respond that it is not clear which tissue to use to further characterize the eQTL results, but Supplementary Table 17 clearly lists tissues corresponding to each result. As presented, the results indicate only that these genes are expressed in a tissue evaluated by GTEx. The analyses are insufficient to call these results (page 14, line 46) “evidence linking our loci to effector genes” and then to subsequently name genes “with eQTL evidence” on page 15. At least for the genes reported as having eQTL evidence, ensure that the sentinel GWAS variant (or it’s closest proxy; weaker proxies do not provide supporting evidence) could feasibly be responsible for that gene’s eQTL association in at least one tissue.

Alternatively, the eQTL results could be removed from the manuscript.

*Response: While we agree with the reviewer that colocalization analysis is key to proving a link between our GWAS signals and target gene eQTLs in specific tissues, here we simply used eQTLs as another line of evidence to prioritize further functional follow-up of three loci with Promoter CaptureC variant-to-gene evidence. This played a role in our decision-making (see Supplementary Figure 6), so we believe its removal would make the choice of loci for functional follow-up difficult for the reader to follow. Indeed, this was an area in which we were critiqued during the first round of review, and in response, we added Supplementary Figure 6 and justifications in the text as to the reasons the three loci were chosen for functional work.
Therefore, we have edited the text to refer to eQTLs as “LD-eQTLs” and highlight that these were used as a further prioritization step, but not as definitive colocalization. Indeed, our in vitro functional follow-up experiments in themselves provide the needed evidence that our GWAS signals are physically connected to target genes in relevant bone cell types that are unavailable in GTEx. As such, we have downplayed the eQTL evidence throughout the text, and highlight that this approach was simply used as an additional filter to prioritize loci for in vitro functional analysis.
We have edited the eQTL text throughout, and added the following text to p.14:
“Colocalization evidence would be needed to definitively link our GWAS signals with these genes in the developing skeleton, but due to the lack of relevant eQTL datasets in growing bone, we instead used this LD-eQTL pan-tissue evidence to help prioritize loci for functional in vitro follow-up.”*

2. (Minor) In response to major comment 9, edits to Figure 3 removed several figure panels corresponding to two loci (now in Supplementary Figure 7), but the legend to Figure 3 still describes three loci with parts A through G and does not describe the forest plots or SITAR curves by genotype.

*Response: We have now edited the figure legend to better reflect the contents of the revised figure.*

**Third round of review**

**Reviewer 2**

The authors have addressed my concerns
